# Substrate-Based Design of Cytosolic Nucleotidase IIIB Inhibitors and Structural Insights into Inhibition Mechanism

**DOI:** 10.3390/ph15050554

**Published:** 2022-04-29

**Authors:** Dorota Kubacka, Mateusz Kozarski, Marek R. Baranowski, Radoslaw Wojcik, Joanna Panecka-Hofman, Dominika Strzelecka, Jerome Basquin, Jacek Jemielity, Joanna Kowalska

**Affiliations:** 1 Division of Biophysics, Institute of Experimental Physics, Faculty of Physics, University of Warsaw, Pasteura 5, 02-093 Warsaw, Poland; dkubacka1@gmail.com (D.K.); mateusz.kozarski@fuw.edu.pl (M.K.); marek.baranowski@fuw.edu.pl (M.R.B.); joanna.panecka@uw.edu.pl (J.P.-H.); dominika.strzelecka@student.uw.edu.pl (D.S.); 2 Centre of New Technologies, University of Warsaw, Banacha 2c, 02-097 Warsaw, Poland; r.wojcik7@student.uw.edu.pl; 3 Department of Structural Cell Biology, Max-Planck-Institute of Biochemistry, Am Klopferspitz 18, D-82152 Martinsried, Germany; basquin@biochem.mpg.de

**Keywords:** nucleotidase, cN-IIIB, NT5C3B, nucleotide-based inhibitor, 7-methylguanosine

## Abstract

Cytosolic nucleotidases (cNs) catalyze dephosphorylation of nucleoside 5’-monophosphates and thereby contribute to the regulation of nucleotide levels in cells. cNs have also been shown to dephosphorylate several therapeutically relevant nucleotide analogues. cN-IIIB has shown in vitro a distinctive activity towards 7-mehtylguanosine monophosphate (m^7^GMP), which is one key metabolites of mRNA cap. Consequently, it has been proposed that cN-IIIB participates in mRNA cap turnover and prevents undesired accumulation and salvage of m^7^GMP. Here, we sought to develop molecular tools enabling more advanced studies on the cellular role of cN-IIIB. To that end, we performed substrate and inhibitor property profiling using a library of 41 substrate analogs. The most potent hit compounds (identified among m^7^GMP analogs) were used as a starting point for structure–activity relationship studies. As a result, we identified several 7-benzylguanosine 5’-monophosphate (Bn^7^GMP) derivatives as potent, unhydrolyzable cN-IIIB inhibitors. The mechanism of inhibition was elucidated using X-ray crystallography and molecular docking. Finally, we showed that compounds that potently inhibit recombinant cN-IIIB have the ability to inhibit m^7^GMP decay in cell lysates.

## 1. Introduction

Cellular pools of nucleotides and nucleosides are regulated by cytosolic 5’-nucleotidases (cNs) [1,2,3]. In humans, so far, eight 5’-nucleotidases have been identified. These enzymes catalyze hydrolytic 5′-dephosphorylation of non-cyclic ribo- and deoxyribonucleoside monophosphates [4,5]. cNs differ in substrate specificity, cellular localization, and tissue-specific expression [1]. Together with kinases, which are responsible for phosphorylation of nucleosides and nucleotides, 5′-nucleotidases regulate the metabolism of purine and pyrimidine nucleotides, maintaining the balance between their synthesis and degradation in cells [2]. All 5’-nuclotidases have broad substrate specificity, and consequently, in addition to their physiological activity, can also dephosphorylate 5’-phosphate esters of therapeutically relevant nucleoside analogs [1,2,6]. This activity negatively affects pharmacological efficacy of nucleoside analogs and limits their clinical use [7,8,9]. 5’-Cytosolic nucleotidase IIIB (cN-IIIB) is one of the most recently discovered 5’-nucleotidases and has several unique structural and functional features. It has been characterized in human and *Drosophila*. The first report revealing hydrolytic activity of cN-IIIB towards m^7^GMP appeared in 2013, and a year later, this property has been shown also for a homologous enzyme–human cytosolic 5′ nucleotidase IIIA (cN-IIIA) [5,10]. Despite high sequence identity between cN-IIIA and cN-IIIB (identity at the level of 56%, and similarity at 80%), the enzymes demonstrate slightly different substrate specificity. cN-IIIA primarily hydrolyzes pyrimidine nucleotides; so far, the high activity of this protein has been shown towards CMP, UMP, dCMP, dUMP, and dTMP, and lower but also specific activity was observed for m^7^GMP [10]. In contrast to cN-IIIA, cN-IIIB is characterized by wider substrate specificity. cN-IIIB has shown a strong preference for the pyrimidine nucleotides CMP and UMP, but it dephosphorylates purine nucleotides AMP and GMP, albeit much less efficiently [5]. However, one of the most preferred cN-IIIB substrates is m^7^GMP, for which the activity of dephosphorylation is characterized by one order of magnitude lower K_M_ constant compared to the other preferred substrates, whereas *k*_cat_/*K*_M_ is comparable [5]. Interestingly, a study on cN-IIIB enzyme from *Drosophila* revealed that the enzyme hydrolyses not only m^7^GMP but also m^7^GDP [5], featuring an additional pyrophosphatase activity. The activity of 5′ nucleotidase III depends on the presence of Mg^2+^ ions, and the optimum pH is 7.5. Additionally, for cN-IIIA, phosphotransferase activity was also observed, i.e., the enzyme can catalyze phosphate group transfer from donor nucleotide to a nucleoside acceptor [11]. The cellular function of cN-IIIA enzyme has been characterized very well. It was first identified in red blood cells, where it plays a crucial role in the degradation of RNA metabolites formed during erythrocyte maturation [11,12,13]. The lack of cN-IIIA activity in red blood cells is correlated with hemolytic anemia due to accumulation of pyrimidine nucleotides [13]. The cN-IIIA enzyme has been also involved in deactivation of certain nucleoside-derived drugs, such as AraC or AZT [11,14,15]. In contrast to cN-IIIA, little has been revealed so far about the actual cellular function of cN-IIIB. Due to the unique preference of cN-IIIB nucleotidase towards m^7^GMP, which is the product of mRNA decapping [16,17], it has been speculated that the enzyme might also be involved in degradation of mRNA metabolites. 

Herein, we aimed to designed specific cN-IIIB inhibitors suitable for probing the function and regulating activity of cN-IIIB in cells. To ensure the high specificity of the inhibitors, we chose the m^7^GMP molecule as a structural template, since it is a unique substrate differentiating cN-III nucleotidases from cN-I and cN-II, which prefer AMP and GMP/IMP, respectively, as substrates [1,18,19].

In this study, we applied a structure-based approach to discover specific inhibitors of the human cN-IIIB enzyme. To that end, we screened an in-house library of nucleotide analogs, which were mostly derivatives of unique cN-IIIB substrates (m^7^GMP and m^7^GDP) or its first reported inhibitor (m^7^G-triazol-P; **S14**) [20]. By modifying the initial hits, we finetuned the inhibitory properties and selectivity towards cN-IIIB, which afforded compounds with micromolar potency and good selectivity. To get deeper insight into the inhibition mechanism, we performed crystallographic and molecular dynamics studies of cN-IIIB in complex with identified inhibitors. Finally, we tested if the in vitro potency of the identified inhibitors correlates with their ability to inhibit m^7^GMP dephosphorylation in human cell extracts. 

## 2. Results and Discussion

### 2.1. Substrate and Inhibitor Properties Screening

**Figure 1:**
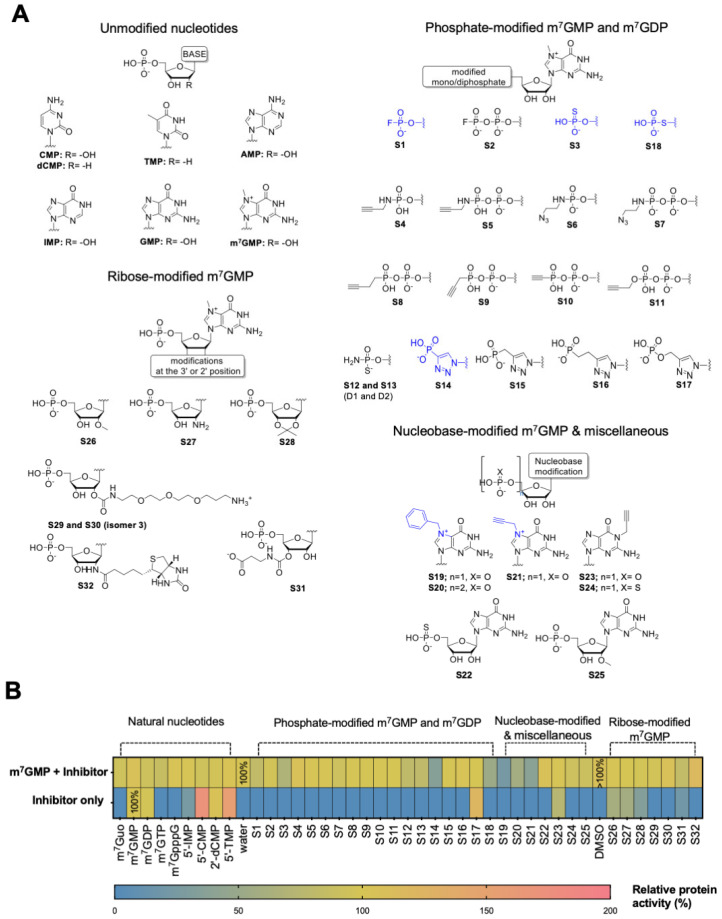
Evaluation of the first-generation library of compounds as cN-IIIB substrates and inhibitors. (**A**) Chemical structures of all four groups of nucleotides from the library. (**B**) Heat map of inhibitory potency and hydrolysis of 41 compounds of library I. Inhibitory potency was measured as the % of substrate dephosphorylation, m^7^GMP, by cN-IIIB enzyme in the presence of the inhibitor. Depending on the solubility of compounds, their stock solution was dissolved in water or DMSO, and the graph presents the control reaction for both solvents. The data represent mean values ± SD from duplicate.

To gain insight into the molecular recognition requirements of human cN-IIIB, we collected a library of 41 nucleotides and evaluated them as substrates and inhibitors of the enzyme. The library consisted of natural nucleotides and nucleotide analogs, mostly m^7^GMP and m^7^GDP derivatives carrying different nucleobase, phosphate, and ribose modifications (Figure 1A). The natural nucleotides and triazole-containing m^7^GMP analogs **S14–S17** have been previously studied in the context of substrate or inhibitor properties towards cN-IIIB [5,10,20], whereas all other compounds were studied for the first time in this context. The susceptibility to hydrolysis by cN-IIIB and inhibitory potency were tested independently using an HPLC-based assay and malachite green phosphate (MGP) assay. To determine the susceptibility to enzymatic hydrolysis, the compounds (100 μM) were incubated in the presence of cN-IIIB (80 nM) for 45 min followed by MGP assay. m^7^GMP was dephosphorylated under these conditions in about 40%. The dephosphorylation efficiency for all other tested nucleotides was reported relative to efficiency of m^7^GMP (which was set to 100%; Figure 1B, Table S3). As expected, the unmodified (natural) pyrimidine 5’-monophospates (CMP, dCMP, TMP) were dephosphorylated with comparable or higher efficiency than m^7^GMP (100–160% relative activity) [5], whereas IMP was a less efficient substrate (25% relative activity). m^7^GDP was also dephosphorylated by cN-IIIB under these conditions, whereas m^7^GTP and m^7^GpppG were not. Nearly all m^7^GMP and m^7^GDP analogs modified within the phosphate group (**S1–S18**) were not susceptible to hydrolysis by cN-IIIB. The only exception was compound **S17**, which possessed phosphoester triazole moiety and was previously confirmed as a cN-IIIB substrate [20]. The bulky ribose modifications (**S29**, **S30**, and **S32**) and the substitution of N7-methyl group for benzyl or propargyl (**S19–S21**) also prevented hydrolysis by cN-IIIB. Only the compounds with small ribose modifications (**S26–S28** and **S31**) and compound with carrying N1-propargylguanosine (**S23**) were susceptible to hydrolysis. 

To preliminarily evaluate the inhibitory potency of the compounds, we tested their influence on the rate of m^7^GMP hydrolysis by cN-IIIB. To that end, m^7^GMP (100 μM) was incubated in the presence of the tested inhibitor (100 μM) and cN-IIIB (80 nM) for 45 minutes. The amount of 7-methylguanosine (m^7^G) released during this reaction was determined using HPLC-based assay and normalized to the amount of m^7^G released in the absence of any inhibitor. Among all m^7^GMP and m^7^GDP analogs, only seven compounds notably inhibited cN-IIIB activity. The most potent inhibitory effect was observed for the substitution of the N7-methyl for benzyl or propargyl moiety (compounds **S19**, **S20**, and **S21**). Coincidently, these compounds were also resistant to hydrolysis by cN-IIIB, which made them excellent candidates for further studies. In contrast, most of the compounds containing various phosphate modifications, despite their resistance to hydrolysis, were not potent inhibitors. Only compounds with fluorophosphate (**S1**), thiophosphate (**S3** and **S18**), and phosphotriazole moiety (**S14**) inhibited cN-IIIB to some extent. None of the natural or ribose-modified nucleotides showed inhibitory activity. 

**Figure 2:**
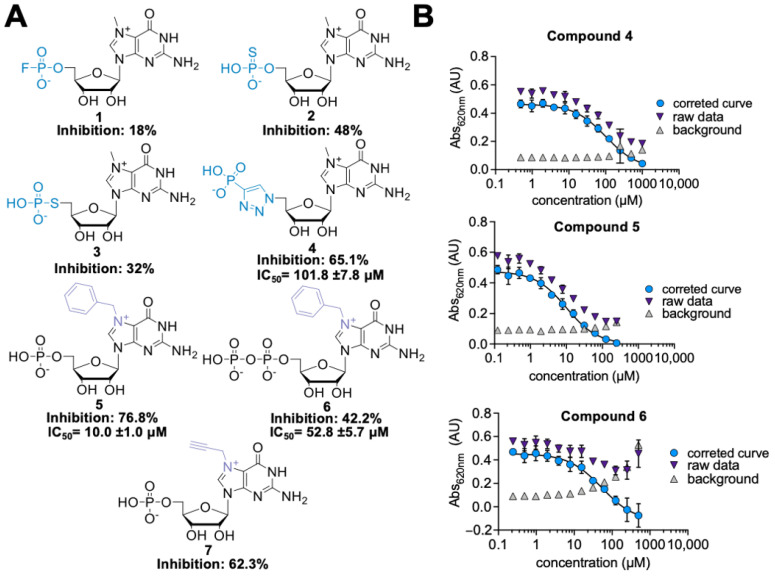
(**A**) Structures of the most potent hits identified in the first library of cN-IIIB inhibitors. (**B**) IC_50_ curves for compounds **4**, **5**, and **6**. The IC_50_ values were determined using MGP assay. The data present mean values ± SD from triple independent experiments. To determine the IC_50_ values, a standard dose-response equation was fitted to the data corrected for the background absorbance of the inhibitor alone.

Seven the most potent hits from the first-generation inhibitors are shown in Figure 2 (compounds **1–7**), together with their relative percentage of inhibition values. Among phosphate modifications, the previously identified 5’-triazole moiety conferred the highest inhibitory potency and was characterized by high chemical stability (which was an issue for compounds **2** and **3**, data not shown). For the most potent and stable cN-IIIB inhibitors (**4**, **5**, and **6**), we determined the IC_50_ values using MGP assay (Figure 2B) (Table 1). Comparing the IC_50_ values, we found that compound **5** (7-benzylguanosine 5’-monophosphate; Bn^7^GMP) was the strongest inhibitor of cN-IIIB, being 10-fold more potent than the previously identified triazole-based compound **4** [20]. 

### 2.2. Design of the Second Library of Potential Inhibitors

**Figure 3:**
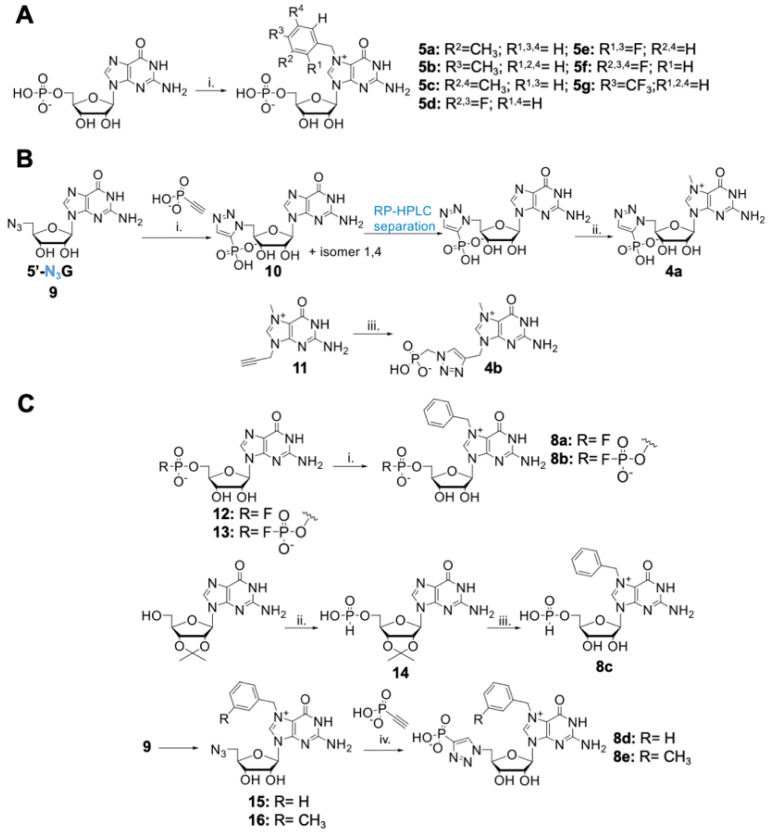
Synthesis of second-generation inhibitors. (**A**) Synthesis of Bn^7^GMP derivates: (i) appropriate substituted benzyl bromide; 8–10 equivalents, DMSO, 45 °C, 24 h. (**B**) Synthesis of triazole-modified m^7^GMP analogs: (i) DMF, 90 °C, 72 h; (ii) CH_3_I-8 equivalents, DMSO, 3 h, 37 °C; (iii) 3-(methyl)-benzyl bromide 1 equivalent or benzyl bromide 6 equivalents, DMF, 24 or 6 h, respectively; (iv) C_2_HPO_3_-2 or 2.5 equivalents, CuSO_4_ 0.2 equivalents, sodium ascorbate 1.2 equivalents, DMSO/water, 2 h, rt. (**C**) Synthesis of 5’-mono- and difluorophosphate and 5’-H-phosphonate 7-benzylguanosine analogs: (i) benzyl bromide, 8–10 equivalents, DMSO (0.5 mL/15 mg of nucleotide), 24 h, 45 °C; (ii) diphenyl phosphite 2.75 equivalents, pyridine, 48 h; (iii) benzyl bromide-6 equivalents, DMF, 50 °C, 2.5 h, then 35% HCl, 72 h, rt.

Three compounds characterized by the highest inhibition values (**4**, **5**, and **6**) were further evaluated and used as templates for structure-guided design of the second-generation inhibitors. The first group of novel potential inhibitors consisted of Bn^7^GMP (**5**) derivatives carrying different substituents at the benzyl group. The second group consisted of triazole-based analogs of m^7^GMP, including 1,5-isomer of compound **4** (**4a**) and ribose-subtracted analog (**4b**). In the last group of inhibitors, we took as templates Bn^7^GMP or closely related compounds and combined these structures with different phosphate modifications: 5’-fluromono- and 5’-fluorodiphosphate, 5’-H-phosphonate, and 5’-phosphotriazole (**8a**–**e**) (Figure 3, Table S2). 

### 2.3. Chemical Synthesis of Second-Generation Inhibitors

**Figure 4:**
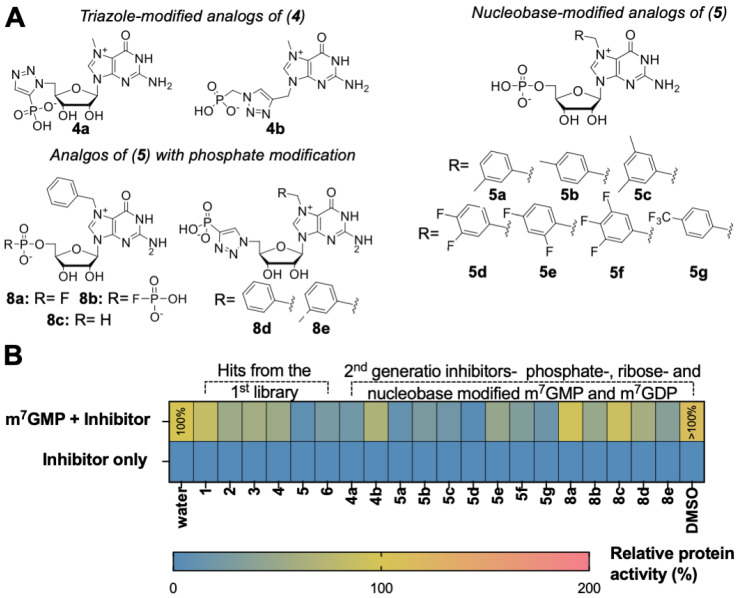
Evaluation of the second-generation inhibitors. (**A**) Structures of the potential second- generation inhibitors of cN-IIIB enzyme. (**B**) Inhibitory potency and hydrolysis of 14 compounds of the second library, which includes 6 leading compounds from the first library. Inhibitory potency was introduced as the % of m^7^GMP—substrate dephosphorylation by cN-IIIB enzyme in the presence of inhibitor. Depending on the solubility of compounds, their stock solution was dissolved in water or DMSO, and the graph presents the control reaction for both solvents. The data represent mean values ± SD from triplicate.

All designed compounds were obtained by chemical synthesis starting from commercially available or previously reported building blocks. The first group of potential inhibitors, carrying different benzyl derivatives, were synthesized by alkylation of N7-position of guanosine using substituted benzyl bromides (R-BnBr; compounds **5a**–**g**; Figure 3A). The second group, consisting of triazole-modified analogs, was obtained by azide-alkyne cycloaddition as the key step. To obtain compound **10**, 5’-azido-5’-deoxyguanosine (**9**) [21] was reacted at elevated temperature with ethynyl phosphonate to obtain a mixture of 1,4- and 1,5-isomers, which were next separated using RP HPLC. The 1,5-isomer, **10**, was then methylated at the N7-position with CH_3_I to yield the target compound **4a** (Figure 3B). Compound **4b** was obtained by reacting 7-mehtyl-9-propargyl guanine (**11**) with azidomethyl phosphonate under CuAAC conditions. Finally, compounds **8a–c**, which combined the presence of N7-benzyl with phosphate moiety modifications, were synthesized by benzylation of N7-postion of corresponding phosphate-modified GMP analogs: guanosine 5’-mono- and 5’-difluorophosphate (**12** and **13)**, and guanosine 5’-H-phosphonate (**14**) (Figure 3C). Compounds **8d** and **8e**, in turn, were synthesized using previously optimized CuAAC reaction. To obtain compounds **8d** and **8e,** N7-benzyl and N7-(3-methyl)benzyl-substituted 5’-azido-5’-deoxyguanosine, derivatives **15** and **16**, were synthesized. The resulting azido-derivatives were then reacted with ethynyl phosphonate^22^ [22] under CuAAC conditions to produce compounds **8d** and **8e,** respectively (Figure 3C). Overall, we obtained a set of 14 novel m^7^GMP analogs, which were examined closely as substrates and inhibitors of cN-IIIB enzyme (Figure 4A, Table S2). The structures of all isolated compounds were determined by HRMS and NMR analysis.

### 2.4. Substrate and Inhibitory Properties of Second-Generation Inhibitors

We first tested if the second-generation compounds are susceptible to cN-IIIB under assay conditions analogous as applied for first library testing. As expected, none of the m^7^GMP analogs were susceptible to enzymatic hydrolysis by cN-IIIB (Figure 4B, Table S4). Next, we verified the inhibitory properties of the compounds. Since they were all resistant to hydrolysis, the inhibitory activity was reliably evaluated using MGP assay, which was more straightforward than HPLC analysis. The preliminary screening revealed that most of the designed compounds inhibited cN-IIIB with varying potency (Figure 4B, Table S4). Following preliminary screening, we determined IC_50_ values for second-generation inhibitors (Table 1, Figure S1). To this end, m^7^GMP (100 μM) was incubated with cN-IIIB (80 nM) and 12-points half dilution of an inhibitor starting from 1000 (**8a**, **8b**, **8d,** and **8e**) or 500 (**4b**, **5b**, **5e**, **5f,** and **8c**) or 250 μM (**5a**, **5c**, **5d,** and **5g**) for 45 min. 

The results confirmed that substitution of guanosine at the N7-position with benzyl derivatives carried the greatest inhibitory potential among all modifications. The most potent inhibitors were compounds **5a** and **5d**, carrying 3-(methyl)-benzyl and 3,4-(difluoro)-benzyl moieties at the N7-position (IC_50_ values of 2.3 ± 0.3 and 2.5 ± 0.2 μM, respectively). The combination of replacement of the N7-benzyl with either fluorophosphate (compound **8a**), fluorodiphosphate (**8b**), or H-phosphonate (**8c**) groups did not enhance the inhibitory properties (IC_50_ values of >**500** and **62.8 ± 16.8** and >**500** μM, respectively). This suggests that the negative charge located at the 5’-phosphate group is crucial for tight binding with cN-IIIB. The combination of triazole-containing analogs with benzyl or 3-(methyl)-benzyl moiety at the N7-position slightly increased inhibitory properties of those compounds (**8d** and **8e)** compared to compound **4** but did not produce inhibitors more potent than Bn^7^GMP (**5**). Interestingly, configuration change of the triazole ring significantly enhanced inhibitory potency. Compound **4a**, which carries the 1,5-substituted 1,2,3-triazole ring, inhibited m^7^GMP dephosphorylation approximately three-fold more efficiently than 1,4-substituted compound **4** (Table 1 and Table S2).

**Table 1:** The IC_50_ values for second-generation inhibitors of cN-IIIB enzyme and parent compounds **4, 5,** and **6**.

Compound	IC_50_ ± SEM (µM)—cN-IIIB
**4**	101.8 ± 7.8
**5**	10.0 ± 1.0
**6**	52.8 ±5.7
**4a**	35 ± 15
**4b**	>500
**5a**	2.3 ± 0.3
**5b**	14.3 ± 1.8
**5c**	13.9 ± 2.0
**5d**	2.5 ± 0.2
**5e**	27.6 ± 2.6
**5f**	12.3 ± 2.6
**5g**	7.3 ± 0.9
**8a**	>500
**8b**	63 ± 17
**8c**	>500
**8d**	89 ± 27
**8e**	144 ± 23

### 2.5. Selectivity of Second-Generation Inhibitors Relative to Cap-Dependent Proteins (eIF4E) and Cytosolic 5’-Nucletidases IIIA 

To verify the selectivity of the identified most potent cN-IIIB inhibitors (**5**, **5a**, 5**d,** and **5g**), we investigated them in the context of two other known proteins capable of recognizing m^7^GMP, eukaryotic translation initiation factor 4E (eIF4E) [23,24] and cN-IIIA. To assess the affinity of cN-IIIB inhibitors towards eIF4E, we applied a competition binding assay based on a pyrene-labelled fluorescent probe [25]. In the assay, we used mouse eIF4E (400 nM), a pyrene-labelled probe (75 nM), and cN-IIIB inhibitor (10-point half log dilution series starting at 100 μM). The fluorescence of pyrene-labelled probe in complex with eIF4E was quenched due to interaction with the protein. The increasing concentration of the tested inhibitors caused displacement of probe from the binding site and an increase in fluorescence. On the basis of the obtained dose-dependent curves, we calculated EC_50_ values of the inhibitors, which reflect their relative binding affinities for eIF4E (Table 2, Figure S2). The determined EC_50_ values were compared to m^7^GMP (EC_50_ 8.4 ± 1.7 μM), which is a known ligand with affinity for eIF4E (*K*_D_ ~ 1 µM) [26]. To better compare the relative properties of the studied inhibitors, we also calculated the selectivity index (SI), which we defined as the ratio of IC_50_ for cN-IIIB to EC_50_ to eIF4E (i.e., the lower the SI, the higher selectivity for cN-IIIB). The data demonstrated that compounds **5**, **5a**, **5d,** and **5g** had at least two-fold lower affinity to eIF4E compared to m^7^GMP. Compound **5g** had the lowest affinity for eIF4E (EC_50_ > 50 μM), making the SI low, but its inhibitory potency towards cN-IIIB was also relatively weak. Among the most potent cN-IIIB inhibitors (**5**, **5a**, **5d,** and **5g**) compound **5d** was characterized by the highest affinity for cN-IIIB combined with low SI. Thus, **5d** displayed the highest selectivity for cN-IIIB over eIF4E. 

Next, we determined the inhibitory potency of the same inhibitor set towards cN-IIIA enzyme. cN-IIIA is encoded by the same gene as cN-IIIB and has 80% similarity in the sequence [5,10]. cN-IIIA is known to prefer pyrimidine nucleotides as substrates [10], but also hydrolyses m^7^GMP, albeit with lower efficiency. To determine the inhibitory potency towards cN-IIIA, compounds were evaluated using the MGP assay in an analogous way as described above for cN-IIIB. The determined IC_50_ values and SI for **5**, **5a,** and **5d** were comparable to each other, and all indicated good selectivity for cN-IIIB (Table 2, Figure S5). The most potent inhibitor was compound **5g** (IC_50_ 42.5 ± 8.3 μM). Compound **5g** had slightly better inhibitory properties towards cN-IIIA, which resulted in lower SI. Overall, the data obtained for eIF4E and cN-IIIA suggested that compounds **5a** (7-(3methyl)- benzyl guanosine 5’-monophophate) and **5d** (7-(3,4-difluoro)-benzyl guanosine 5’-monophosphate) were characterized by very good selectivity for cN-IIIB.

### 2.6. Structural Insight into Human cN-IIIB

**Figure 5:**
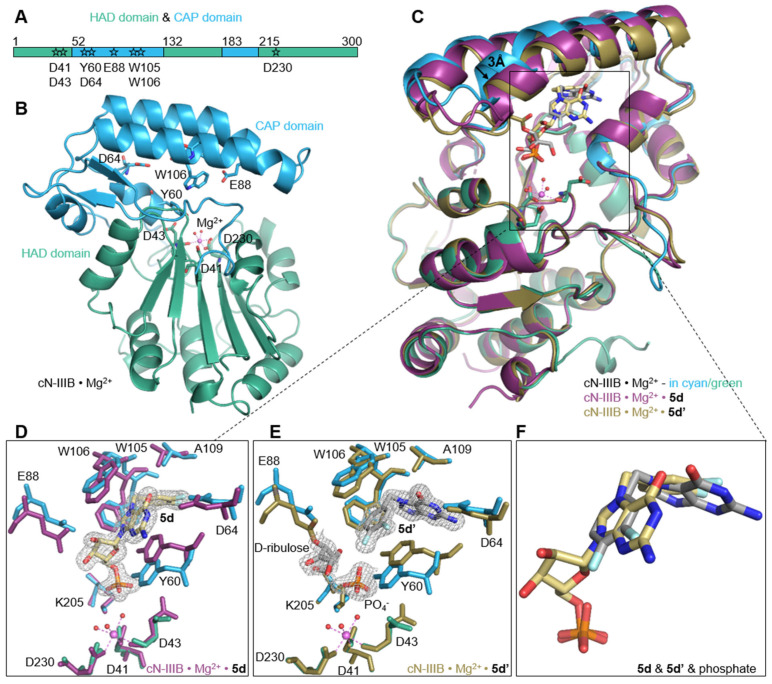
Structural rearrangements of the human cN-IIIB enzyme upon ligand binding. (**A**,**B**) Schematic representation of the domain structure of the enzyme and the cartoon representation of the crystal structure of cN-IIIB • Mg^2+^ complex. CAP and HAD domain are colored in blue and green, respectively. The residues responsible for cap recognition are located in the CAP domain and the aspartic acids, which coordinate magnesium ion (magenta sphere) and form the catalytic center, are located in the HAD domain. The residues are shown as sticks in the structure and are marked as stars on the domain scheme. (**C**) Structural alignments showing the movement of the CAP domain under substrate binding (cN-IIIB • Mg^2+^• **5d** obtained by soaking aligned to cN-IIIB • Mg^2+^ and cN-IIIB • Mg^2+^• **5d**’ obtained by cocrystalization with **5d** aligned to cN-IIIB • Mg^2+^). (**D**,**E**) The rearrangements of active site residues upon inhibitor binding and (**F**) alignment of inhibitors **5d** and **5d’**.

To gain better insight into the molecular mechanism of hydrolytic resistance of Bn^7^GMP and derivatives to cN-IIIB and their ability to potently inhibit the enzyme, we attempted to crystalize the compounds in the complex with cN-IIIB. Our initial trials of cocrystalization revealed the tendency of the compounds to decompose with the cleavage of N-glyosidic bond, as some the initially obtained crystal structures showed three separate electron densities, one corresponding to the modified nucleobase, a second corresponding to ribose moiety, and a third resembling the tetrahedral geometry of orthophosphate (Figure 5). After extensive optimization studies including other crystallization methods such as soaking and microseeding, we were able to solve three crystal structures at high resolution: the apo form of nucleotidase in complex with magnesium ion: cN-IIIB • Mg_2_^+^ (1.36 Å; PDB id: 7ZEE), and two structures of nucleotidase in complex with ligands and magnesium ion: cN-IIIB • Mg^2+^ • **5d’** (3,4-diF-Bn^7^Guanine; 1.56 Å; PDB id: 7ZEG) and cN-IIIB • Mg_2_^+^ • **5d** (3,4-diF-Bn^7^GMP; 1.5 Å; PDB id: 7ZEH) (Figure 5). 

To date, these are the first coordinates obtained for human cytosolic nucleotidase IIIB. The crystal structure of cN-IIIB consists of two distinct domains: the globular core domain (residues 1–51, 132–182, and 215–300) and the smaller CAP domain (52–131 and 183–214; Figure 5A,B). The core domain is a haloacid dehalogenase domain (HAD). It has three-layer architecture α/β/α and harbors a portion of the catalytic site including residues that coordinate the magnesium ion. The second domain mainly consists of a bundle of helices that are responsible for recognizing the ligand. The C-terminal part of the protein (residues 291–300) and one of a loop connecting domains (residues 211–220) were not defined in the electron density map, and thus are most likely disordered. 

### 2.7. Interactions Highlighting Selectivity of Inhibitor Binding

**Figure 6:**
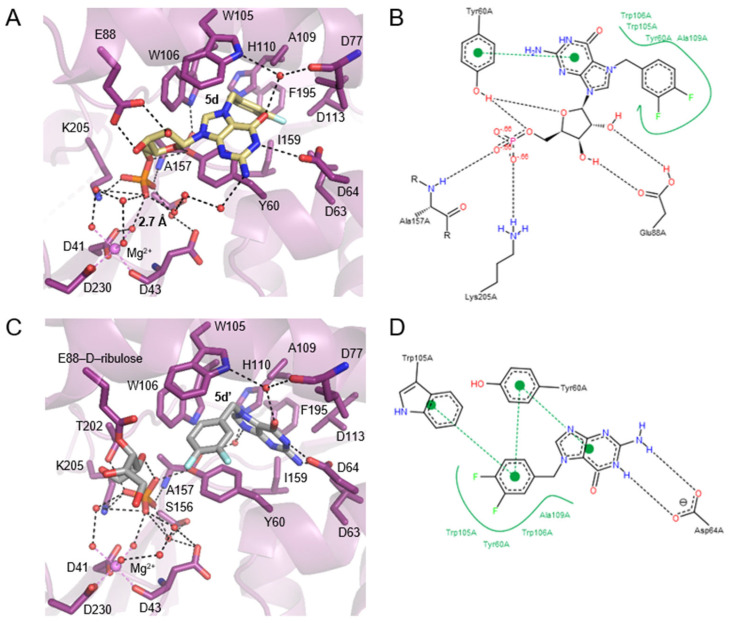
Structural insight into cN-IIIB inhibitor binding and close up view of interactions between **5d/5d’** and human cN-IIIB. (**A**) The structure of the enzyme in complex with compound **5d**, shown in yellow carbon, obtained by soaking, while the complex structure presented in (**C**) was obtained by cocrystallization of compound **5d** with the enzyme. The inhibitor **5d** in (**C**) decomposed to **5d’**-*N7*-(3,4-diflorobenzyl)guanine, D-ribulose, both presented in gray carbon, and phosphate. cN-IIIB is shown in a purple cartoon representation, and relevant residues are shown in sticks, where dark blue is nitrogen, red—oxygen, orange—phosphate, and light blue—fluoride. Hydrogen bonds are shown as a dashed black line, and magnesium and its coordinated bonds are marked in violet. (**B**,**D**). Pose view representations of interactions between ligands and cN-IIIB, with hydrophobic interactions shown as a green line and hydrogen bonds shown as black dashed lines.

In the structure of cN-IIIB in complex with 3,4-diF-Bn^7^GMP (**5d**), each moiety of the inhibitor is stabilized by direct contact with various protein residues. The 3,4-difluorobenzyl group of the modified base is accommodated in a hydrophobic cavity formed by sidechains of Trp105, Trp106, Tyr 60, Ala109, Leu63, Leu77, Leu113, and Ile159 (Figure 5C,D and Figure 6A,B). Then, the guanine base intercalates between the off-centered π-ring system of Tyr 60 and Trp105 separated by typical stacking distances of 3.6 Å between the side chains and the base. Tyr60 is located on the short movable α-helix and can easily adjust to form centered cation–π interactions with N7-modified guanine ring, while Trp105, located on one of the long helices of the CAP domain, can rotate only around its Cα–Cβ bond to engage in stacking interactions. Thus, the aromatic ring of Trp105 is in parallel-displaced geometry towards the base ring (Figure 5C,D and Figure 6A,B). Additionally, the sidechain of Trp106, which is in an edge-to-face geometry against guanine base (3.7 Å distance to C8 of guanine), participates in stabilizing interactions and shields the ligand from the surrounding. The hydroxyl group of Tyr 60 is additionally engaged in interacting with the ribose ring, which adopts *O-4*’-endo conformation. Tyr 60 forms a hydrogen bond with the O4’ atom, while the 2’- and 3’-oxygens of the ribose are hydrogen bonded with the carboxylic group of highly conserved Glu 88. The phosphate group is placed in a binding cavity in the vicinity of amine group of Lys205 and amide group of Ala157, which are in direct contact with phosphate oxygens. Additional hydrogen bonds via structural water molecule with hydroxyl residue of Ser156 and hydrogen bond of Tyr60 with 5’-*O*-oxygen support the stabilizing contact of phosphate group with nucleotidase and simultaneously distance it from the catalytic center by 2.7 Å (Figure 6A). The observed shift of the phosphate center away from the catalytic site may explain why the compound does not undergo dephosphorylation despite tight binding to the protein. Although the hydrophobic interactions are generally weak, the complementarity of the 3,4-difluorobenzyl group to the shape of hydrophobic side pocket contributes significantly to the stabilizing contacts of the complex. Those additional interactions of the substituted benzyl group with the enzyme as well as the direct stabilizing contacts with polar amino acid side- chains of the enzyme make 3,4-F_2_Bn^7^GMP an efficient competitor of binding site against m^7^GMP substrate.

Human cN-IIIB also efficiently accommodates *N7*-(3,4-diflorobenzyl)guanine (**5d’**) in its hydrophobic binding pocket. Compound **5d’** is a decomposition product of 3,4-diFBn^7^GMP readily formed under crystallization conditions. The crystal structure revealed that cN-IIIB binds **5d’** using the same hydrophobic moieties as in the case of **5d**; however, the aromatic rings of **5d’** are reversely oriented compared to **5d** (Figure 5C,E,F and Figure 6C,D). For **5d’**, the guanine ring is accommodated in the hydrophobic cavity, where it is stabilized by hydrogen bonds between N1 and N2 and carboxylate group of Asp64. The guanine moiety participates in additional water-mediated interactions with a side chain of His 100 and backbone carbonyl of Ala157. The 3,4-difluorobenzyl moiety stacks in centered-parallel orientation between Trp105 and Tyr60, while the guanine ring is in edge-to-face stacking contact with Tyr60. This structure suggested that *N7*-(3,4-diflorobenzyl)guanine may be a compound structurally tailored for cN-IIIB, making it a potential candidate for inhibitor. 

In the complex structure of cN-IIIB • Mg_2_^+^ with **5d’** solved with 1.56 Å resolution, two additional electron densities were observed. The first one with tetrahedral shape belonged to the phosphate group (Figure 5E), while an extensive analysis of the shape of second electron density revealed the structure of D-ribulose, which is likely formed during decomposition of **5d**. The distance of < 1.5 Å between D-ribulose and oxygen of Glu88 carboxylic group indicates a covalent linkage between the molecules (Figure 5E and Figure 6C).

### 2.8. The Comparison of cN-IIIB Crystal Structures

The differences between solved structures: the holoenzyme of cN-IIIB • Mg^2+^, and the enzyme in complex with inhibitors: **5d** or **5d’**, concern mainly two helices of the CAP domain, which move slightly down and left towards HAD domain by about 3 Å (Figure 5C). This domain movement likely allows for accommodating the compounds in the 7-methylguanine-biding pocket, despite their relative bulkiness. Ligand-binding triggers substantial changes of Tyr60 and Trp105 sidechain conformations to form a system of aromatic rings that stabilize the complex. Significant movement under ligand binding is also visible for the sidechain of Glu88, which forms hydrogen bonds to ribose moiety in **5d** or covalent link to D-ribulose in the case of **5d’** (Figure 5D,E and Figure 6A,C). The phosphate group found in cN-IIIB • Mg^2+^ • **5d’** complex adopts the same binding site as phosphate of **5d** in the complex with cN-IIIB (Figure 5F). Additionally, we compared the human cN-IIIB with the crystal structure of *Drosophila* cN-IIIB • Mg^2+^ nucleotidase in complex with 7-methylguanosine (PDB id: 4NV0), which was solved in the presence of MgF_3_^-^. The MgF_3_^-^ mimics the pentavalent phosphate transition state in the nucleotidase reaction cycle^10^. The structural alignment of *Drosophila* and human cN-IIIB shows that the phosphate group of **5d** occupies a different binding site than MgF_3_^-^, which is likely placed at the catalytic center. Comparisons CAP of domains revealed that human cN-IIIB stays in a more open conformation than the domain of *Drosophila* cN-IIIB. All the above suggest that ligand **5d** freezes the protein conformation in an open state of the enzyme. A similar effect was observed for the structure with **5d’**; the root mean square deviation for all Cα atoms of cN-IIIB with **5d’** and **5d** was only 0.42 Å. Of note, the ligand binding does not induce any changes in the HAD domain. In all three crystal structures, the magnesium ion was octahedrally coordinated by the carboxyl side chains of catalytic moieties of Asp 41 and Asp 230 and the backbone carbonyl of Asp 43 and by three water molecules. The lack of significant changes in the arrangement of catalytic site suggested that the major reason for resistance of **5d** to dephosphorylation stems from locking the enzyme in the open conformation. 

### 2.9. Molecular Docking Provides an Explanation for the Inhibition Mode

To gain more insight into inhibition modes and verify if compound **5d** and the other N7-benzyl derivatives indeed lock the enzyme in an open state, we employed molecular docking experiments. Comparison of the obtained human cN-IIIB and homologous crystal structures (Figure 5 and Figure S7) suggested that the human cN-IIIB enzyme may significantly change its conformation, especially the C1-type cap domain, holding the ligands bound in the active site. Due to the high large-scale mobility of the enzyme near the active site, we decided to dock the compounds to both the open and closed enzyme conformations, the former, crystallized in this work, and the latter, obtained through homology modeling (Figure S6). To further improve the docking results, the induced-fit approach was used.

**Figure 7:**
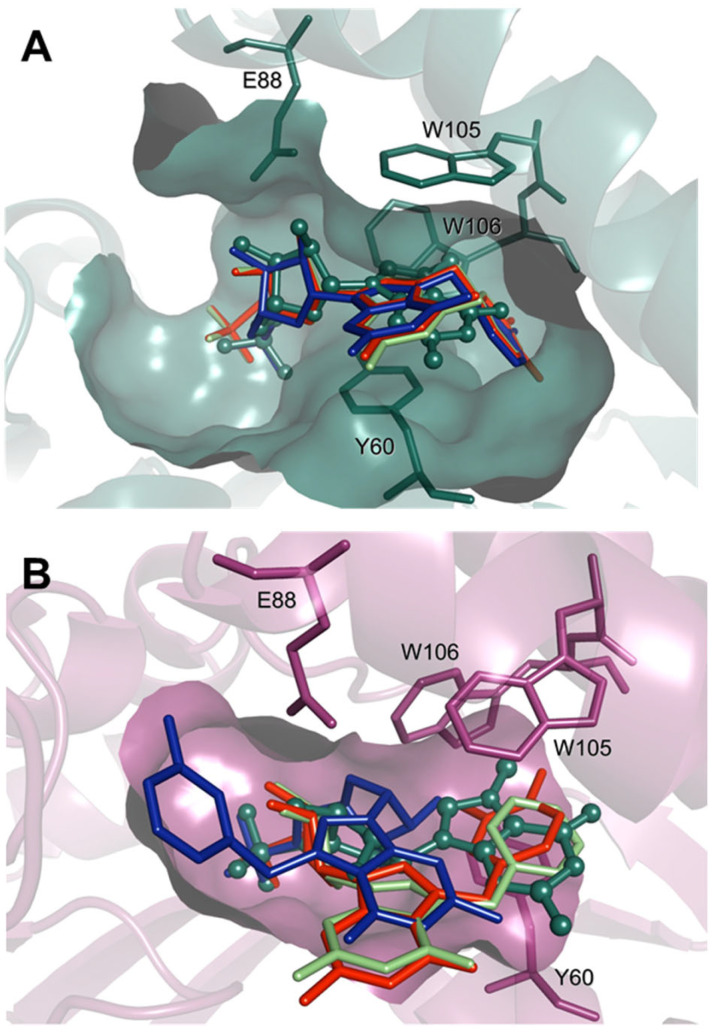
Comparison of the top docking poses of compounds **5** (lime), **5a** (blue), and **5d** (red) in the open (**A**) and closed (**B**) enzyme conformations. The substrate, m^7^GMP, is shown as a reference in ball-and-stick representation (green). For clarity, only heavy atoms and selected pocket residues are shown.

Initial docking simulations, performed for natural m^7^G-derived substrates and products (Figure S7), revealed that the poses fairly accurately reproduce the expected binding modes, observed in the crystal structures of *Drosophila* cN-IIIB (with 7-methyl-guanosine, PDB code: 4NV0) and *M. musculus* cN-IIIA (with uridine-5’-monophosphate, PDB code: 4FE3). Moreover, we found that the main analyzed enzyme substrate, m^7^GMP, binds more favorably to the closed enzyme conformation (Table S6), which is thus likely the catalytically active one. Then, we performed docking simulations of compound **5** and its analogues. These simulations revealed that the compounds with N7-benzyl substituents can only bind in the substrate-like conformation to the open form of the enzyme (Figure 7A), since the binding site in the closed conformation is too compact to accommodate N7-substituted nucleobases, bulkier than m^7^Gua (Figure 7B and Figure S6). Compound **5** and derivatives poorly fit in the pocket of the closed receptor, and in many poses, the 7-benzyl substituent, too large to bind more deeply in the pocket, is stacked between Trp 105 and Tyr 61, i.e., occupying the pocket of m^7^Gua in m^7^GMP (Figure 7B). Since the compounds of this series are active and are predicted to bind in the substrate-like conformation only to the open enzyme form, we might again hypothesize that they block the enzyme in the open form. As for the ranking of 7-substituted derivatives, scoring did not seem to correlate well with activities. This may be related to the problem with accurate modelling the induced fit of this highly dynamic enzyme. Secondly, the lack of correlation between score and activity is a known pitfall of classical docking methods. Considering more than one enzyme conformation and using the induced-fit approach helped in better understanding of the inhibitor binding modes in the likely dynamic cN-IIIB enzyme but does not really ensure that if a reasonable binding mode (in terms of interactions) was not observed (as for 7-benzyl derivatives), it is not possible for another enzyme conformation that was not modelled. Because the enzyme movements are large scale, classical molecular dynamics would also not improve the results, and the only way to perform a more in-depth study of the dynamics of the ligand–enzyme interactions would be using enhanced sampling techniques, which could be applied in the future to better explore this aspect of the cN-IIIB enzyme functioning.

### 2.10. Synthesis and Investigation of Inhibitory Properties of N7-Substituted Guanine Analogs

We discovered that inhibitor **5d** decomposed to *N7*-[3,4-(difluoro)-benzyl]-guanine **5d’** and D- ribulose under crystallographic conditions. Compound **5d’** was bound by cN-IIIB in the same place as **5d**, but in reverse orientation; was well stabilized by the hydrogen bond network; and it was perfectly aligned in the CAP-binding domain of cN-IIIB. This fact prompted us to evaluate N7-benzyl-substituted guanine derivatives as cN-IIIB inhibitors. To that end, we synthesized four novel guanine derivatives, carrying benzyl (**5’**), 3-methylbenzyl (**5a’**) or 3,4-difluorobenzyl (**5d’)** at the N7-position by a modification of a previous procedure (Figure S8) [27]. Additionally, we obtained *N7, N9- bis*[3,4-difluorobenzyl]guanine **5d’’**.

**Figure 8:**
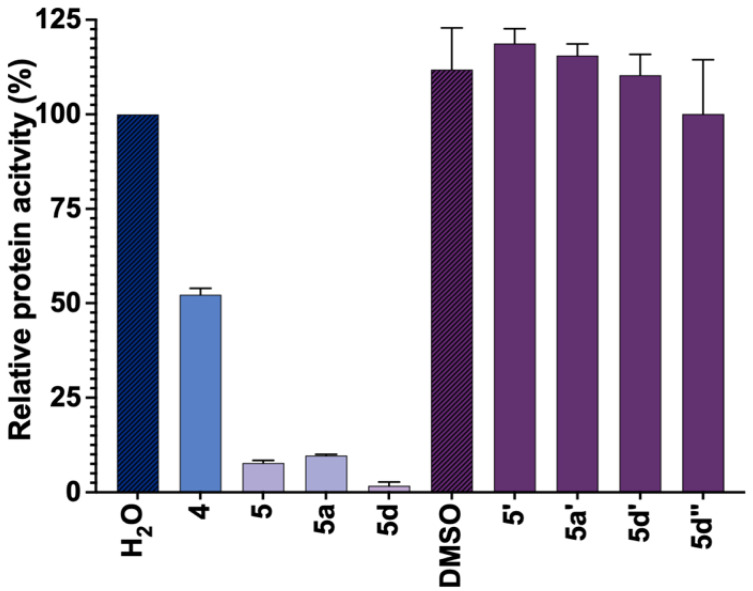
Screening of the third library of inhibitors. Inhibitory potency of compounds **5′**, **5a′**, **5d′**, and **5d″** as compared to their nucleotide counterparts (**5**, **5a,** and **5d**) (each at 100 µM). Compound **4** was used as a positive control. Depending on the solubility of compounds, their stock solution was prepared in water (blue) or DMSO (purple), and hence the control reactions for both solvents are shown. The data represents mean value ± SD from duplicate experiment.

To examine the inhibitory potency towards cN-IIIB, compounds **5’**, **5a’**, **5d’,** and **5d’’** were evaluated at a single concentration (100 μM) using MGP assay. The results for **5’**, **5a’**, **5d’,** and **5d’’** were compared to their nucleotide counterparts (Figure 8). The data revealed that **5’**, **5a’**, **5d’,** and **5d’’** did not inhibit cN-IIIB under these conditions. Hence, despite the observation of N7-benzylguanine analogs in the crystallographic structures, the N7 benzyl guanine analogs did not inhibit cN-IIIB enzyme at concentrations up to 100 µM, which suggests that the depurination may have occurred only after nucleotide–ligand binding. 

### 2.11. Activity of cN-IIIB Inhibitors in HEK 293T Cell Lysate

**Figure 9:**
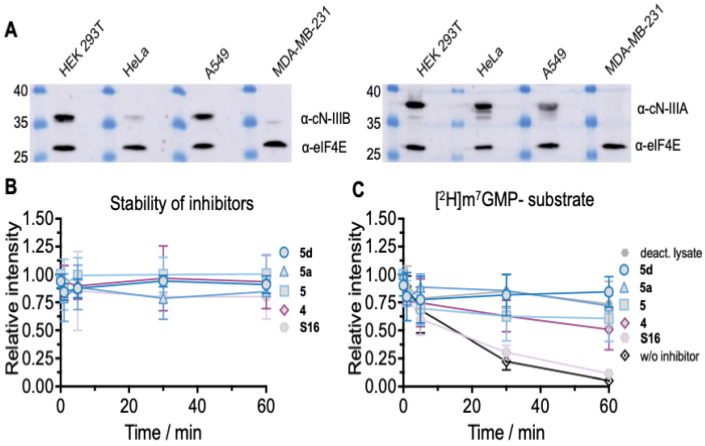
(**A**) Protein expression profile of endogenous level of cN-IIIB and cN-IIIA in different cell lines. As a reference, the expression of eIF4E protein was shown. (**B**) Stability of indicated compounds in HEK 293T cell lysate determined by LC–MS/MS. (**C**) m^7^GMP decay in HEK 293T cell extract in the presence of compounds with different inhibitory potencies. Data points represent mean values from triplicate experiments ± S.D.

Finally, to preliminary evaluate the ability of most potent compounds to inhibit endogenous cN-IIIB, we evaluated select compounds as inhibitors of m^7^GMP dephosphorylation in cytoplasmic HEK 293T cell extracts using LC–MS/MS. The expression of cN-IIIB was verified by Western blot and was the most abundant in HEK 293T among four tested cell lines (Figure 9A). The cN-IIIA isoform was likewise abundantly expressed in the HEK cell line. The stability and activity of the inhibitors were tested in HEK 293T cell extracts using mass spectrometry analysis. The evaluated set of compounds contained some of the identified cN-IIIB inhibitors: **4**, **5**, **5a,** and **5d** of varying inhibitory potencies as well as compound **S16** with no inhibitory potency (negative control). Initially, we verified the stability of the compounds in the extracts. The LC–MS/MS analyses confirmed that all analyzed compounds were stable in the lysate for at least 60 min (Figure 9B). Then, we monitored the time-dependent degradation of heavy m^7^GMP, deuterated within the methyl group ([^2^H]-m^7^GMP]) in the extracts, in the presence or absence of the evaluated compounds (100 µM each, Figure 9C). In the absence of any inhibitor or in the presence of negative control (**S16**), almost complete decay of m^7^GMP was observed within the first 50 min. m^7^GMP decay was notably inhibited by compounds **5**, **5a,** and **5d**, and to a lesser extent by **4**. The observed ability to inhibit m^7^GMP decay in the lysate correlated well with the inhibitory potency of compounds measured for recombinant cN-IIIB (**5d** ≈ **5a** < **5** < **4 << S16**, starting from the most potent). This correlation strongly suggests that cN-IIIB is the major activity responsible for m^7^GMP decay in HEK 293T cells and confirms that the inhibitors developed here may be useful as research tools for studying cN-IIIB activity in more complex biological settings.

## 3. Conclusions

In this study, we set out to develop novel potent and selective inhibitors of cN-IIIB, one of the most recently identified cytosolic nucleotidases. As a starting point for the design of putative inhibitors, we chose m^7^GMP, which is the most unique substrate for cN-IIIB. By screening a library of differently modified m^7^GMP analogs and related compounds, we identified structural features that conferred resistance to enzymatic hydrolysis and inhibitory potency. Among modifications decreasing susceptibility to cN-IIIB were phosphate modifications, bulky ribose modifications, and bulky substitutions of the N7-methyl group attached to nucleobase (guanosine) moiety. Among those, the nucleobase modifications provided compounds with highest inhibitory potency, wherein Bn^7^GMP (**5**) was the most potent compound. Further structure–activity relationship studies enabled us to optimize the structure of the lead compound yielding two compounds (3-MeBn^7^GMP—**5a,** and 3,4-F_2_Bn^7^GMP—**5d**) with low micromolar IC_50_ for cN-IIIB and high selectivity with regards to other proteins capable of recognizing m^7^GMP (eIF4E and cN-IIIA). Crystallographic and molecular docking studies performed for some of the most potent compounds suggested that the inhibition mechanism relies on blocking the enzyme in a catalytically inactive, open conformation. Finally, we have also shown that the developed compounds inhibit dephosphorylation of m^7^GMP in HEK cell lysates, and thus are potentially applicable for modulating the activity of endogenous cN-IIIB. However, further studies in cell culture models are required to uncover the full scope and limitations for the use of the compounds. In particular, it is yet to be established if cellular permeability of the compounds will be sufficient to use them as research tools or if it will be necessary to convert them into phosphate-masked prodrugs. 

## 4. Material and Methods

### 4.1. General

All reagents used for the synthesis were obtained from commercial sources and used without further purification or drying. Guanosine was purchased from Carbosynth. All benzyl bromides were purchased from Sigma-Aldrich.

### 4.2. Analytical and Semi-Preparative Chromatography

Analytical chromatography was performed on HPLC system Agilent Tech. Series 1200 using Supelcosil LC-18-T HPLC column (4.6 × 250 mm, flow rate 1.3 mL/min) or Gemini® 3 μm NX-C18 110 Å (4.6 × 150 mm, flow rate 1 mL/min) with linear gradients of methanol in 0.05 M ammonium acetate buffer and UV detection at 254 nm and fluorescent detection (ex. 260 em. 370 nm). Semi-preparative chromatography was performed on the same HPLC system Agilent Tech. Series 1200 using a Discovery RP-Amide C16 column (21.2 × 250 mm; 5 μm; flow rate 5.0 mL/min) column (A) or Hi Chrom Vydac Denali 5 μm, C18 120 Å (150 × 10 mm) (B), or Gemini 5 μm, NX-C18 110 Å (150 × 10 mm) (C) with linear gradient of acetonitrile in 0.05 M ammonium acetate buffer and UV detection at 254 nm.

### 4.3. Ion-Exchange Chromatography

Ion-exchange chromatography was performed on DEAE-Sephadex A-25 resin with linear gradient of 0–0.6M triethylammonium bicarbonate in water with UV–VIS detection at 260 nm using a UV 1800 Shimadzu apparatus. The final concertation of purified compounds was determined in 0.1 M phosphate buffer pH 6 (for N7-substitued analogs) or pH 7 (for GMP analogs). For calculations, molar extinction coefficients were used: ε_260_ = 11,400 (M^−1^ cm^−1^) for N7-substitued analogs and ε_260_ = 12,080 (M^−1^ cm^−1^) for GMP analogs. 

### 4.4. NMR Spectroscopy

The NMR spectra of intermediate and final compounds were recorded using a Bruker AVANCE III HD 500 MHz spectrometer with a 5 mm Z119470_0188 (PA BBO 500S1 BBF-H-D-05 Z SP) probe at 500.24 MHz for ^1^H NMR and 202.49 MHz for ^31^P NMR or Varian 400 MHz with OneNMR_W016 probe at 399.90 MHz (^1^H NMR), 161.89 MHz (^31^P NMR), and 376.25 (^19^F NMR). The chemical shifts (δ_H,_ δ_P,_ δ_C_ and δ_F_) were reported in ppm scale and referenced to internal standard sodium 3-(trimethylsilyl)-2,2’, 3,3’ tetradeuteropropionate (TSP) (^1^H NMR; ^13^C NMR, 0 ppm) and external standard: 20% phosphoric acid in D_2_O (^31^P NMR; 0 ppm) and sodium fluoride in D_2_O (^19^F NMR; −121.5 ppm). Coupling constant values (J) were reported in Hz. All spectra were recorded at 25.2 °C in deuterated solvents purchased from Sigma-Aldrich. 

### 4.5. Procedures for the Synthesis of Nucleotides

#### 4.5.1. Compound **10 **

5’-Azido-5’-deoxyguanosine (100 mg, 0.3 mmol, 1 equiv.) was dissolved in dry DMF 600 μL and mixed with ethynyl phosphonate TEAH^+^ salt (124 mg, 0.6 mmol, 2 eq.) suspended in DMF 300 μL. Then, the reaction was heated up to 90 °C and stirred for 72 h. After 72 h, 5’-azido-5’-deoxyguanosine was converted into two regioisomers of the product (1,4 and 1,5). The reaction mixture was diluted with water and purified by ion-exchange chromatography DEAE Sephadex A-25. Obtained regioisomers were separated by RP HPLC with semi-preparative column (C). Compound **10** (isomer, 1,5, 16.2 mg, 0.04 mmol) was obtained as ammonium salt with 13.3% yield.

^1^H NMR δ_H_ (399.90 MHz, D_2_O, TSP) 7.88 (1H, s, H_triazol_), 7.81 (1H, s, H8), 5.88 (1H, d, J = 4.0 Hz, H1’), 5.05 (1H, dd, J = 15.0, 4.6 Hz, H5’), 4.98 (1H, dd, J = 15.0, 5.0 Hz, H5’’), 4.81 (1H, overlapped with water, H2’), 4.60 (1H, dd, J = 10.4, 5.0 Hz, H4’), 4.50–4.47 (1H, m, H3’); ^31^P NMR δ_P_ (161.89 MHz, D_2_O, phosphoric acid) −3.84 (1P, s).

#### 4.5.2. Compound **4a** (7-Methylguanosine-triazol-p (1,5 Isomer))

Compound **10** (8 mg, 0.02 mmol, 1 equiv.) was dissolved in DMSO 200 μL. Next, iodomethane (21 mg, 0.16 mmol, 8 equiv., 9.5 μL) was added. The reaction mixture was heated up to 37 °C and stirred for 3 hours. After 3 hours, the reaction mixture was diluted with water and washed with ethyl acetate. The aqueous phase was purified using RP HPLC with semi preparative column (C). Compound **4a** (4.2 mg, 0.01 mmol) was obtained with 50% yield.

^1^H NMR *δ*_H_ (399.90 MHz, D_2_O, TSP) 8.97 (1H, s, H8), 7.87 (1H, s, H_triazol_), 5.97 (1H, d, J = 2.5 Hz, H1’), 5.10 (1H, dd, J = 15.1, 4.9 Hz, H5’), 5.04 (1H, dd, J = 15.1, 3.9 Hz, H5’’), 4.77 (1H, overlapped with water, H2’), 4.65–4.60 (1H, m, H4’), 4.37 (1H, dd, J = 7.2, 5.3 Hz, H3’), 4.10 (3H, s, CH_3_); ^31^P NMR δ_P_ (161.89 MHz, D_2_O, phosphoric acid) −3.90 (1P, s) HR MS ESI [M–H]^-^ *m*/*z* found: 427.08875 (calculated for C_13_H_16_N_8_O_7_P^−^, 427.08850).

#### 4.5.3. Compound 16 (7-(3-Methylbenzyl)-5’-azido-5’-Deoxyguanosine)

5’-Azido-5’-deoxyguanosine (100 mg, 0.32 mmol, 1 equiv.) was dissolved in DMF 800 μL. Then, 3-(methyl)-benzyl bromide (59.8 mg, 0.32 mmol, 1 equiv., 43.7 μL) was added. The reaction mixture was incubated for 24 hours at 30 °C. The progress of the reaction was monitored using a TLC plate. After 24 h, the reaction mixture was diluted with water (800 μL) and washed 4 times with ethyl acetate. The aqueous fractions were combined and freeze-dried, giving compound **16** (42.2 mg, 0.1 mmol) with 32% yield. 

^1^H NMR δ_H_ (500.24 MHz, D_2_O, TSP) 7.35–7.20 (5H, m, aromatic), 6.01 (1H, d, J = 3.2 Hz, H1’), 5.59 (2H, d, J = 3.5 Hz, CH_2_), 4.39 (1H, dd, J = 6.2, 5.0 Hz, H3’), 4.33-4.30 (1H, m, H4’), 3.83 (1H, dd, J= 13.8, 3.2 Hz, H5’), 3.74 (1H, dd, J= 13.8, 4.6 Hz, H5’’). Peak from H2’ was overlapped with water.

^13^C NMR (125.80 MHz, D_2_O) δ_c_ 158.18, 157.27, 152.41, 142.24, 136.39, 135.30, 132.74, 132.43, 131.84, 131.81, 131.78, 131.66, 128.31, 128.13, 124.50, 122.59, 121.60, 110.68, 92.90, 85.65, 76.29, 72.39, 55.30, 53.51, 37.26, 23.08, 23.02, 10.99.

#### 4.5.4. Compound **8e** (7-(3-Methylbenzyl)guanosine-Triazol-p) 

Compound **16** (5 mg, 0.0125 mmol, 1 equiv.) was dissolved in 250 μL of water and mixed with aqueous solution of ethynyl phosphonate (5 mg, 0.025 mmol, 2.0 equiv., TEA salt) of 30 μL. Then, aqueous solution of copper sulfate (0.625 mg, 0.0025 mmol, 0.2 equiv., 7 μL) was added, followed by addition of aqueous solution of sodium ascorbate (3 mg, 0.015 mmol, 1.2 equiv., 30 μL). The reaction mixture was stirred at room temperature for 2 h. After 2 h, the reaction mixture was quenched by adding Na_2_EDTA (2.8 mg, 0.0075 mmol, 0.6 equiv.) dissolved in 50 μL of water. The final compound **8e** was isolated from the reaction mixture using ion-exchange chromatography DEAE Sephadex A-25 and RP HPLC with semi-preparative column (B). Compound **8e** was obtained as ammonium salt (2.2 mg, 0.004 mmol) with 32% yield. 

^1^H NMR δ_H_ (500.24 MHz, D_2_O, TSP) 8.08 (1H, s, H-triazol), 7.42-7.22 (4H, m, H-aromatic), 5.96 (1H, d, J = 3.95 Hz, H1’), 5.65 (2H, s, CH_2_), 4.94 (2H, d, J = 4.90 Hz, H5’, H5’’), 4.74 (1H, dd, J = 5.11, 3.95 Hz, H2’), 4.60–4.53 (1H, m, H4’), 4.50–4.44 (1H, m, H3’), 2.35 (3H, s, CH_3_); ^31^P NMR δ_P_ (202.49 MHz, D_2_O, phosphoric acid) 0.52 (1P, s).

^13^C NMR (125.80 MHz, D_2_O) δ_c_ 158.38, 152.51, 142.17, 135.68, 132.51, 131.77, 131.37, 127.90, 93.31, 85.02, 75.37, 72.82, 55.24, 22.98.

HR MS ESI [M-H]^-^ *m*/*z* found: 517.13572 (calculated for C_20_H_22_N_8_O_7_P^-^, 517.13546).

#### 4.5.5. Compound **15** (7-Benzyl-5’-azido-5’-Deoxyguanosine)

5’-Azido-5’-deoxyguanosine (230 mg, 0.75 mmol, 1 equiv.) was dissolved in 2 mL of DMF. Then, benzyl bromide (766 mg, 4.5 mmol, 6 equiv., 533 μL) was added. The reaction mixture was stirred at room temperature for 6 hours. After 6 hours, the reaction mixture was diluted with 5 mL of water, followed by washing with ethyl acetate. The aqueous phase was freeze-dried, yielding (159 mg, 0.4 mmol) **15** with 53% yield. 

^1^H NMR δ_H_ (500.24 MHz, D_2_O, TSP) 7.47 (6H, s, aromatic), 6.02 (1H, d, J = 3.5 Hz, H1’), 5.66 (2H, s, CH_2_), 4.40 (1H, dd, J = 6.3, 4.7 Hz, H3’), 4.34–4.30 (1H, m, H4’), 3.83 (1H, dd, J = 13.4, 2.9 Hz, H5’), 3.74 (1H, dd, J = 13.4, 4.6 Hz, H5’’). Peak from H2’ was overlapped with water. ^13^C NMR (101 MHz, D_2_O) δ_c_ 129.31, 129.21, 128.41, 82.84, 73.47, 69.66, 50.69.

#### 4.5.6. Compound **8d** (7-Benzylguanosine-Triazol-p)

Compound **15** (101.5 mg, 0.25 mmol, 1 equiv.) was dissolved in 3 mL of water and mixed with aqueous solution of ethynyl phosphonate (128 mg, 0.625 mmol, 2.5 equiv., 0.3 mL, TEA salt). Then, aqueous solution of copper sulfate (12.5 mg, 0.05 mmol, 0.2 equiv., 0.07 mL) was added, followed by the addition of aqueous solution of sodium ascorbate (59.4 mg, 0.3 mmol, 1.2 equiv., 0.2 mL). The reaction mixture was stirred at room temperature for 2 hours. After 2 hours, the reaction mixture was quenched by adding Na_2_EDTA (56 mg, 0.15 mmol, 0.6 equiv.) dissolved in 0.5 mL of water. The final compound **8d** was isolated from the reaction mixture using ion-exchange chromatography DEAD Sephadex and RP HPLC with semi-preparative column (B). Compound **8d** was obtained as ammonium salt (54 mg, 0.1 mmol) with 40% yield. ^1^H NMR δ_H_ (500.24 MHz, D_2_O, TSP) 8.06 (1H, s), 7.51–7.41 (5H, m), 5.96 (1H, d, J = 3.96 Hz), 5.67 (2H, s), 4.92 (2H, d, J = 5.01 Hz), 4.70 (1H, dd, J = 3.96, 5.28 Hz), 4.59–4.54 (1H, m), 4.46–4.41 (1H, m); ^31^P NMR δ_P_ (202.49 MHz, D_2_O, phosphoric acid) 0.37 (1P, s). HR MS ESI [M–H]^−^ *m*/*z* found: 503.11937 (calculated for C_19_H_20_N_8_O_7_P^-^, 503.11981).

#### 4.5.7. Compound **4b** (7-methylguanosine-9-CH_2_-Triazol-CH_2_-p)

Compound **11** (2 mg, 0.01 mmol, 1 equiv.) and azidomethyl phosphonate (5 mg, 0.02 mmol, 2 equiv.) were dissolved in DMF 80 μL. Then, aqueous solution of copper sulfate (0.5 mg, 0.002 mmol, 0.2 equiv., 7 μL) was added, followed by the addition of aqueous solution of sodium ascorbate (2 mg, 0.01 mmol, equiv., 20 μL). The reaction mixture was stirred for 18 hours at room temperature. After 18 hours, the reaction mixture was quenched by aqueous solution of Na_2_EDTA (2.8 mg, 0.0075 mmol, 0.6 equiv., 50 μL). The final compound **4b** was isolated from the reaction mixture using both ion-exchange chromatography DEAE Sephadex A-25 and RP HPLC with semi preparative column (B). After freeze-drying, compound **4b** (1 mg, 0.003 mmol) was obtained with 29% yield. 

^1^H NMR δ_H_ (500.24 MHz, D_2_O, TSP) 8.22 (1H, s, H8), 5.55 (2H, s, CH_2_), 4.59 (2H, d, J = 11.83 Hz, CH_2_), 4.08 (3H, s, CH_3_); ^31^P NMR δ_P_ (202.49 MHz, D_2_O, phosphoric acid) 9.42 (1P, s). [M–H]^-^ *m*/*z* found: 339.07260 (calculated for C_10_H_12_N_8_O_4_P^-^, 339.07246).

#### 4.5.8. General Procedure A for N7-benzyl Substituted GMP Analogs

The N7-benzyl-substituted GMP analogs were synthesized according to a procedure previously described [28]. Triethylammonium salt of guanosine 5’-monophosphate (1 equiv.) was suspended in DMSO (0.5 mL/15 mg of nucleotide), and appropriate benzyl bromide was added (8–10 equiv.). The mixture was shaken vigorously at 45 °C for 24 h. The progress of the reaction was monitored by RP HPLC. Then, the reaction was diluted with water (approx. 10-time excess) and extracted with diethyl ether (3 × 10 mL). Organic fraction was rejected, and residuals of organic solvent from water phase were evaporated by rotary evaporator under reduced pressure. Solution in total volume of 10–15 mL was applied onto DEAE-Sephadex A-25 resin. The final product was isolated as triethylammonium salt, and structure was confirmed by high-resolution mass spectrometry (HR MS). 

#### 4.5.9. Compound **5 (S19)** (7-Benzylguanosine 5’-Monophosphate)

**S19 (5)** was prepared according to GP A starting from GMP (150 mg, 3684 mOD, 0.32 mmol), benzyl bromide (442 mg, 307 μL, 2,58 mmol), and DMSO (10 mL), yielding 127 mg of **5** triethylammonium salt (2281 mOD, 0.211 mmol, 66%). HR MS ESI [M–H]^-^ *m*/*z* found: 452.09823 (calculated for C_17_H_19_N_5_O_8_P^-^, 452.09767).

#### 4.5.10. Compound **5a** (7-(3-Methylbenzyl)guanosine 5’-Monophosphate)

**5a** was prepared according to GP A starting from GMP (100 mg, 2260 mOD, 0.21 mmol), 3-(methyl)-benzyl bromide (318 mg, 232 μL, 1,72 mmol), and DMSO (7 mL), yielding 63 mg of **5a** triethylammonium salt (1042 mOD, 0.103 mmol, 49%). HR MS ESI [M–H]^−^ *m*/*z* found: 466.11380 (calculated for C1_8_H_21_N_5_O_8_P^−^, 466.11332).

#### 4.5.11. Compound **5b** (7-(4-Methylbenzyl)guanosine 5’-Monophosphate)

**5b** was prepared according to GP A starting from GMP (150 mg, 3684 mOD, 0.32 mmol), 4-(methyl)-benzyl bromide (478 mg, 2,58 mmol), and DMSO (10 mL), yielding 126 mg of **5b** triethylammonium salt (2215 mOD, 0.205 mmol, 64%). HR MS ESI [M–H]^−^ *m*/*z* found: 466.11362 (calculated for C1_8_H_21_N_5_O_8_P^−^, 466.11332). 

#### 4.5.12. Compound **5c** (7-(3,5-Dimethylbenzyl)guanosine 5’-Monophosphate)

**5c** was prepared according to GP A starting from GMP (100 mg, 2260 mOD, 0.21 mmol), 3,5-(dimethyl)-benzyl bromide (343 mg, 1,72 mmol), and DMSO (7 mL), yielding 47 mg of **5c** triethylammonium salt (1004 mOD, 0.099 mmol, 47%). HR MS ESI [M–H]^−^ *m*/*z* found: 480.12968 (calculated for C_19_H_23_N_5_O_8_P^−^, 480.12879). 

#### 4.5.13. Compound **5d** (7-(3,4-Difluorobenzyl)guanosine 5’-Monophosphate)

**5d** was prepared according to GP A starting from GMP (100 mg, 2260 mOD, 0.21 mmol), 3,4-(difluoro)-benzyl bromide (356 mg, 221 μL, 1,72 mmol), and DMSO (7 mL), yielding 69 mg of **5d** triethylammonium salt (1104 mOD, 0.109 mmol, 52%). HR MS ESI [M–H]^−^ *m*/*z* found: 488.07962 (calculated for C_17_H_17_F_2_N_5_O_8_P^−^, 488.07883). 

#### 4.5.14. Compound **5e** (7-(2,4-difluorobenzyl)guanosine 5’-monophosphate)

**5e** was prepared according to GP A starting from GMP (100 mg, 2260 mOD, 0.21 mmol), 2,4-(difluoro)-benzyl bromide (356 mg, 221 μL, 1,72 mmol), and DMSO (7 mL), yielding 69 mg of **5e** triethylammonium salt (1112 mOD, 0.109 mmol, 52%). HR MS ESI [M–H]^−^ *m*/*z* found: 488.07924 (calculated for C_17_H_17_F_2_N_5_O_8_P^−^, 488.07883). 

#### 4.5.15. Compound **5f** (7-(3,4,5-Trifluorobenzyl)guanosine 5’-Monophosphate)

Compound **5f** was prepared according to GP A. 

HR MS ESI [M-H]^-^ *m*/*z* found: 506.06991 (calculated for C_17_H_16_F_3_N_5_O_8_P^−^, 506.06941). 

#### 4.5.16. Compound **5g** (7-(4-Trifluoromethylbenzyl)guanosine 5’-Monophosphate)

**5g** was prepared according to GP A starting from GMP (100 mg, 2260 mOD, 0.21 mmol), 4-(trifluoromethyl)- benzyl bromide (411 mg, 266 μL, 1,72 mmol), and DMSO (7 mL), yielding 43 mg of **5g** triethylammonium salt (919 mOD, 0.088 mmol, 42%). HR MS ESI [M–H]^−^ *m*/*z* found: 520.08567 (calculated for C_18_H_18_F_3_N_5_O_8_P^−^, 520.08506). 

#### 4.5.17. Compound **8a** (7-Benzylguanosine 5’-Fluoromonophosphate)

Compound **8a** was prepared according to GP A starting from guanosine 5’-fluoromonophosphate (230 mg, 1506 mOD, 0.125 mmol), benzyl bromide (615 mg, 430 μL, 3.6 mmol), and DMSO (6 mL), yielding 73 mg of **8a** triethylammonium salt (582 mOD, 0.051 mmol, 41%). [M–H]^−^ *m*/*z* found: 454.09427 (calculated for C_17_H_18_FN_5_O_7_P^−^_,_ 454.09334).

#### 4.5.18. Compound **8b** (7-Benzylguanosine 5’-Fluorodiphosphate)

Compound **8b** was prepared according to GP A, except that guanosine 5’-fluorodiphosphate was used as a starting material. [M–H]^−^ *m*/*z* found: 534.06042 (calculated for C_17_H_19_FN_5_O_10_P_2_^−^_,_ 534.05967).

#### 4.5.19. Compound **8c** (7-benzylguanosine 5’-H-phosphonate)

Compound **14** (200 mg, 0.51 mmol, 1 equiv.) was dissolved in DMF (6 mL). Then, benzyl bromide (520 mg, 3.1 mmol, 6 equiv., 370 μL) was added to the mixture. The reaction mixture was heated up to 50 °C and mixed for 2.5 h. After 2.5 h, the reaction mixture was diluted twice with water, 35% hydrochloride acid was added to adjust the pH to 1, and stirring was continued for 72 h. After 72 h, the reaction mixture was washed with diethyl ether. The final compound was purified by ion-exchange chromatography DEAE Sephadex A-25, yielding 102 mg (0.23 mmol) of compound **14** with 45% yield.

^1^H NMR δ_H_ (500.24 MHz, D_2_O, TSP) 7.44 (5H, m, H-aromatic), 6.70 (1H, d, J = 640.4, PH), 6.05 (1H, d, J = 3.78, H1’), 5.65 (2H, s, CH_2_), 4.73 (1H, dd, J = 5.03, 3.78 Hz, H2’), 4.46 (1H, t, 5.08, H3’), 4.42–4.39 (1H, m, H4’), 4.22 (1H, ddd, J = 12.05, 6.03, 2.54 Hz, H5’), 4.13 (1H, ddd, J = 12.05, 7.0, 2.80 Hz, H5’’); ^31^P NMR δ_P_ (202.49 MHz, D_2_O, phosphoric acid) 6.50 (1P, dt, J = 640.4, 6.5 Hz). ^13^C NMR δ_C_ (125.80 MHz, D_2_O, TSP) 155.67, 157.81, 152.66, 136.03, 131.80, 130.76, 110.58, 92.73, 86.87, 86.80, 77.01, 72.12, 64.68, 55.25

[M–H]^−^ *m*/*z* found: 436.10296 (calculated for C_17_H_19_N_5_O_7_P^−^, 436.10276).

#### 4.5.20. General Procedure B for N7 Benzyl-Substituted Guanine Analogs 

Compounds **5’**, **5a’,** and **5d’** were synthetized according to a previously described procedure with minor modifications [27]. Guanosine (1 equiv.) was suspended in DMSO 400 mg/mL. The resulting solution was stirred for 20 minutes at room temperature. Then, appropriate benzyl bromide was added (1.1 equiv.). The reaction mixture was stirred for 24 h at room temperature. After 24 h, the reaction mixture was heated to 70 °C. Then, an aqueous solution of 10% HCl was added (227 mg/mL), and the reaction mixture was stirred for an additional 2 h. Next, the reaction mixture was cooled, and the precipitate was isolated by filtration. The precipitate was washed with cold water, suspended in water, and neutralized with 6 M NaOH. The final product was filtered, washed with cold water, and dried in vacuum without further purification.

#### 4.5.21. Compound **5’** (7-Benzylguanine)

Compound **5’** was prepared according to GP B starting from guanosine (283 mg, 1 mmol, 1 equiv.), benzyl bromide (188.1 mg, 1.1 mmol, 1.1 equiv.), and DMSO 0.75 mL, yielding 16% (38.3 mg, 0.16 mmol). 

^1^H NMR δ_H_ (399.90 MHz; DMSO-*d_6_*; TSP) 10.71 (s, 1H, NH), 8.07 (s, 1H, C8), 7.36–7.26 (m, 4H, aromatic), 6.12 (s, 2H, NH_2_), 5.41 (s, 2H, CH_2_).

#### 4.5.22. Compound **5a**’ (7-(3-Methylbenzyl)guanine) 

Compound **5a’** was prepared according to GP B, starting from guanosine (283 mg, 1 mmol, 1 equiv.), 3-(methyl)-benzyl bromide (203.5 mg, 1.1 mmol, 1.1 equiv.), and DMSO 0.75 ml, yielding 21% (53 mg, 0.21 mmol).

^1^H NMR δ_H_ (399.90 MHz; DMSO-*d_6_*; TSP) 10.70 (s, 1H, NH), 8.04 (s, 1H, C8), 7.26–7.06 (m, 4H, aromatic), 6.11 (s, 2H, NH_2_), 5.37 (s, 2H, CH_2_), 2.26 (s, 3H, CH_3_).

#### 4.5.23. Compound **5d’** (*N7*-(3,4-Difluorobenzyl)guanine) 

Compound **5d’** was prepared according to GP B, starting from guanosine (283 mg, 1 mmol, 1.1 equiv.), 3,4-(difluoro)-benzyl bromide (261.8 mg, 1.1 mmol, 1.1 equiv.), and DMSO 0.75 ml, yielding 36% (98.7 mg, 0.36 mmol). 

^1^H NMR δ_H_ (399.90 MHz; DMSO-*d_6_*; TSP) 10.76 (s, 1H, NH), 8.09 (s, 1H, C8), 7.49–7.17 (m, 3H, aromatic), 6.15 (s, 2H, NH_2_), 5.38 (s, 2H, CH_2_); ^19^F NMR δ_F_ (376.25 MHz; DMSO-*d_6_*) −138.22- (−138.34) (m, 1F), −139.88- (−140.00) (m, 1F)

#### 4.5.24. Compound **5d’’** (*N*7,9- *bis*-(3,4-Difluorobenzyl)guanine)

Guanine (50 mg, 0.33 mmol, 1 equiv.) was suspended in DMSO 0.2 mL. Then, 3,4-(difluoro)-benzyl bromide (471 mg, 1.98 mmol, 6 equiv.) was added. The reaction mixture was stirred for 24 hours at 50 ^o^C. During the reaction, three products were obtained (N7, N9, and N7,9-substituted). After 24 h, the obtained produces were precipitated by addition of 1 mL of water. The precipitate was filtered and washed with diethyl ether. The mixture of isomers was separated by liquid chromatography using silica gel resin. The products were eluted from the silica gel column using DCM/CH_3_OH (from 2 to 20% of CH_3_OH). Combined fractions were evaporated under reduced pressure and dried in vacuum to give a white powder (10.1 mg,0.025 mmol). The **5d’’** was obtained with 7.6% yield.

^1^H NMR δ_H_ (399.90 MHz; DMSO-*d_6_*; TSP) 9.48 (s, 1H, C8), 7.64–7.27 (m, 7H, aromatic), 5.58 (s, 2H, CH_2_), 5.35 (s, 2H, CH_2_); ^19^F NMR δ_F_ (376.25 MHz; DMSO-*d_6_*) −137.77-(−138.00) (m, 2F), −138.65-(−138.77) (m, 1F), and -138.88-(-139.00) (m, 1F). 

### 4.6. Protein Expression and Purification of cN-III Enzymes for Enzymatic Assays

Recombinant human cN-IIIB (gene names: NT5C3B, NT5C3L) was expressed as a construct of full-length protein (residues 1–300) tagged with ubiquitin-like Smt protein and His_6_ at the N term. Conversely, human cN-IIIA (gene names: NT5C3, P5N1, UMPH1) was expressed as shortened, stable protein (residues 58–336) also tagged with ubiquitin-like Smt protein and His_6_ at the N term. Both enzymes were expressed and purified according to a protocol previously described for cN-IIIB^20^. Shortly, each of the constructs were purified on HisTrap FF (GE Healthcare) affinity column and cleaved with His6_Ulp1 protease, and the enzyme was separated from digestion mix on HisTrap FF and purified to homogeneity on a HiLoad 16/600 Superdex 75 pg (GE Healthcare) gel filtration column in Tris buffer (20 mM Tris-HCl (pH 7.5), 150 mM NaCl, 5 mM MgCl_2_, 1 mM DTT). Finally, both human enzymes cN-IIIA (residues 58 -336) and full-length cN-IIIB were aliquoted and stored at −80 °C in the presence of 10% glycerol. Protein purity was assessed by SDS-PAGE electrophoresis, and protein concentration was determined spectrophotometrically using the extinction coefficient calculated from amino acid composition: ε_280_ = 27,390 (M^−1^ cm^−1^) for cN-IIIB and ε_280_ = 23,380 (M^−1^ cm^−1^) for cN-IIIA.

### 4.7. Biological Characterization of Compounds 

#### 4.7.1. Hydrolysis Assay

Susceptibility of library compounds to hydrolysis by human cN-IIIB or cN-IIIA was tested in 96-well, clear, non-binding assay plates (Greiner). The compounds at the concentration of 100 µM were incubated with enzyme, at 80 nM in case of cN-IIIB or 40 nM in case of cN-IIIA, in 20 mM HEPES buffer containing 50 mM KCl and 5 mM MgCl_2_ at pH 7.5 at 30 °C and with shaking at 300 rpm. The reactions progress was quantified using a Malachite Green Phosphate (MGP) Assay Kit (Sigma-Aldrich) according to the manufacturer’s instructions. A total of 20 µL of working reagent containing MG and molybdate were added to 80 µL of reaction mixture to quench the reactions after 45 min for cN-IIIB or 25 min for cN-IIIA. Then, the mixture was incubated for 25 min at room temperature, and this was followed by the determination of absorbance at 620 nm using a microplate reader (Synergy H1 Multi-Mode Reader, BioTek). In the assay the amount of phosphate released in enzymatic reaction was quantified. For most of the compounds, hydrolytic activity of compounds was also verified by the HPLC method with detection at 254 nm. 

#### 4.7.2. Inhibition Assay

Inhibition assays were performed in 96-well, clear, non-binding assay plates (Greiner). Each reaction mixture contained enzyme, substrate (m^7^GMP), and the tested inhibitor dissolved in 80 µL of 20 mM HEPES buffer (pH 7.5) containing 5 mM MgCl_2_ and 50 mM KCl. The reaction components were preincubated for 15 min at 30 °C with shaking at 300 rpm, followed by the addition of the enzyme. The first library screening was performed for (substrate/inhibitor/cN-IIIB) concentrations equal to (40 µM/40 µM/30 nM), and reactions were run for 45 min at 30 °C with shaking at 300 rpm. The reactions were quenched by the addition of formic acid at a final concentration of 4.5%. HPLC analysis was performed using a Supelcosil LC-18-T column (4.6 × 250 mm, 5 mm: flow rate 1.3 mL/min) with a linear gradient of methanol in 0.1 M phosphate buffer (pH 6). In HPLC analysis, the substrate (m^7^GMP), the inhibitor, and the product (7-methylguanosine) were detected at 260 nm. The second library screening was performed for (substrate/inhibitor/enzyme (cN-IIIB/cN-IIIA)) concentrations equal to (100 µM/100 µM/(80 nM/40 nM)) at the same buffer conditions as the first library screening. Enzymatic reactions were run for 45 min in the case of cN-IIIB and for 25 min in the case of cN-IIIA. The reaction progress was analyzed by colorimetric Malachite Green Phosphate Assay described in detail in the hydrolysis section above. All experiments were performed in triplicate. The IC_50_ experiments were performed under the same conditions as the second library screening, but instead of a single concentration of inhibitor, a 12-point half dilution series starting at 0.25 mM, 0.5 mM, or 1 mM was tested. To determine the IC_50_ values, a standard dose–response equation was fitted to the experimental data:

(1) $$y=A_{1}+\frac{A_{2}-{\;A}_{1}}{1+\frac{x}{\mathrm{IC}50}}$$

where A_1_ is the bottom asymptote, A_2_ is the top asymptote, x is inhibitor concentration, and y is the adequate response.

#### 4.7.3. Competition Assay (Probe-eIF4E-Ligand)

To assess the specificity of cN-IIIB inhibitors towards eIF4E protein, we applied the binding assay developed by Kasprzyk et al.[25]. In the assay, we used mouse eIF4E, which was obtained as previously described [25]. As a probe, we applied a pyrene-labeled probe (Figure S9). The reactions were performed in 96-well, black, non-binding assay plates (Grainer). Each well contained an inhibitor (10-point half log dilution series starting at 100 μM), a 400 nM eIF4E protein, and a 75 nM probe. Reaction components were dissolved in 50 mM HEPES buffer (pH 7.2) containing 100 mM KCl and 0.5 mM EDTA. The buffer solution was degassed before each experiment. All reaction components, together with eIF4E protein, were preincubated at 30 °C for 30 minutes with 300 rpm mixing. Then, keeping the same conditions, the fluorescent measurements (ex. 345 nm and em. 378 nm) were performed using a Microplate Reader (Synergy H1 Multi-Mode Reader, BioTek). The data were collected for 1 hour and averaged to further calculations. The EC_50_ values were determined by fitting a standard dose–response curve (equation presented above) to the obtained data.

#### 4.7.4. Reactions in HEK 293 Cell Lysates

A typical reaction contained 1 µL–40 µM (final concentrations 4 µM) of [^2^H]m^7^GMP and 9 µL cell extracts. Inhibitors of cN-IIIB were added to the reaction mixtures from concentrated stock solutions to a final concentration of 100 µM. The samples were incubated at 37 °C in three replicates. One sample of each set contained a nucleotide with deactivated extract. The rest of the samples were collected at the following time points: immediately after adding enzyme (1 min) and after 5, 30, and 60 min. Samples were deactivated by heating for 5 min at 95 °C, centrifuged, and frozen in liquid nitrogen. 

#### 4.7.5. LC–MS/MS Analysis 

Chromatographic separation of nucleotides and their metabolites was achieved on a ZORBAX Eclipse Plus C18 analytical column (3.5 μm, 4.6 mm × 100 mm, Agilent) equipped with Eclipse Plus-C18 analytical guard column (5.0 μm, 4.6 × 12.5 mm). The gradient mobile phase contained the DMH ion pair reagent [29] and comprised two eluents: eluent A, which was 20 mM aqueous DMH adjusted to pH 4.8 with formic acid, and eluent B, which was a 1:1 (*v*/*v*) mixture of acetonitrile and aqueous 20 mM DMH adjusted to pH 4.8 with formic acid. The elution was performed at room temperature (RT) and at a flow rate of 700 μL/min. The gradient was optimized to obtain sufficient chromatographic separation of all analytes of interest. The optimal gradient was 0–50% eluent B in 15 min then 50–100% B in 20 min. Then, the analytes in the HPLC eluate were monitored by MS in multiple reaction monitoring (MRM) mode. MS conditions for individual analytes were optimized directly from the syringe pump. The optimization was performed using 400 μM solutions of individual nucleotides. Optimizations and analyses have been performed in negative ionization mode to maximize the number of fragmentation reactions [30]. The general ion source conditions used in all experiments were as follows: turbo ion-spray voltage: −4500 V, temperature: 250 °C, curtain gas: 30 psi, ion source gas 1: 30 psi, and ion source gas 2: 25 psi.

### 4.8. Western Blotting

HEK293T, HeLa, A549, and MDA-MB-231 cells were grown in DMEM medium (Gibco) supplemented with 10% FBS (Sigma), GlutaMAX (Gibco), and 1% penicillin/streptomycin (Gibco) at 5% CO_2_ and 37 °C. Firstly, cells were washed with ice-cold PBS and collected in lysis buffer (50 mM Tris (pH 8.0), 150 mM NaCl, 1% Triton X-100, 0.5% sodium deoxycholate, 0.1% SDS, and 1x complete EDTA-free protease inhibitor (Roche)). Then, the lysate was clarified by centrifugation 10 min/10,000× *g*, and protein concentration was determined by Bradford assay. A total of 30 μg protein lysate per lane was loaded, and proteins were separated by SDS-PAGE and transferred to a nitrocellulose membrane by wet transfer using a Mini Trans-Blot® cell. Membranes were blocked with 5% milk and probed with primary antibody against the desired protein at RT for 1h, washed in TBST buffer (Tris-buffered saline, 0.1% Tween 20), and again probed with secondary antibody conjugated with horseradish peroxidase. Western blots were subsequently detected by enhanced chemiluminescence (Cytiva). Primary and secondary antibodies and their dilutions were used as follows: NT5C3L (A-9) (sc-398604; 1:200), NT5C3 (E-8) (sc-390782; 1:200), eIF4E (P-2) (sc-9976; 1:500), and mouse –IgGκ BP-HRP (sc-516102; 1:10,000), all purchased from Santa Cruz. PageRuler™ Prestained Protein Ladder (#26616, Thermo Scientific) was used as the molecular weight marker.

### 4.9. Expression and Purification of Human cN-IIIB for Crystallization

For the crystallization experiment, the full-length protein of human cN-IIIB was expressed in BL21 Star pRARE strain in TB medium at 37 °C as a N-terminal His6_Sumo tagged protein. Overexpression was induced at 18 ⁰C with 0.4 mM IPTG. Cells were lysed by sonication in 50 mM Na_2_HPO_4_ (pH 7.5), 250 mM NaCl, and 30 mM imidazole, and the extracts were clarified by centrifugation (35,000× *g*, 30 min, 4 °C). Firstly, the protein was purified on a 5 mL HisTrap HP column (GE Healthcare). Next, in order to remove the His6_Sumo N-terminal protein, it was incubated overnight at 4 °C with Senp2 protease, and the digestion mix was passed back over on the HisTrap nickel column and the flow through was collected. Finally, the untagged cN-IIIB was polished to homogeneity by size exclusion chromatography (SEC) in a buffer of 20 mM Tris (pH 7.5), 150 mM NaCl, 5 mM MgCl_2_, and 2 mM DTT, then concentrated to about 15 mg/mL, flash frozen, and submitted to crystallization trials.

### 4.10. Crystallization of cN-IIIB

The cN-IIIB protein was set up for crystallization at 30 mg/mL in SEC buffer by sitting-drop vapor diffusion in 0.4 μL drops obtained by a mixture of equal volumes of protein and crystallization solution. Crystals appeared in a few days at 4 °C after mixing protein with 0.1 M MES (pH 6.2) and 22–28% PEG 3350. To gain the complex cN-IIIB•Mg^2+^•**5d** structure, one of the crystals was soaked for 1h with 10 mM compound **5d**. Conversely, the complex cN-IIIB•Mg^2+^•**5d**’ structure was obtained in the co-crystallization process. Prior to crystallization setup, the protein was incubated with 10 mM compound **5d** at rt for 1h, and the crystals were gained by hanging drop vapor diffusion mixing equal volume (1 µL + 1µL drop, rt) of protein•ligand and crystallization solution containing 0.1 M MES (pH 5.6), 1.73 M ammonium sulfate, and 5% PEG 400. All crystals were cryoprotected in a reservoir solution containing 30% (*v*/*v*) ethylene glycol before flash freezing in liquid nitrogen.

### 4.11. Data Collection and Structure Determination

X-ray data collection was carried out at 100 K at the X10SA beamline at the Swiss Light Source in Villigen, Switzerland, and the data were processed using XDS [31]. Phases were determined using molecular replacement in Phaser-MR module of Phenix [32]. The structure of the mouse cN-IIIA (PDB id: 2BDU) was used as the search model for cN-IIIB•Mg^2+^ without ligand, and subsequently the solved cN-IIIB structure was used as a model. Ligand dictionaries/descriptions were generated using ProDrg and AceDrg [33], and the link description between Glu88 and D-ribulose was made using JLigand [34]. The models were improved by multiple rounds of manual building in Coot [35], followed by refinement using Phenix.refine [36]. Structure validation was performed using MolProbity [37]. Data collection and refinement statistics are summarized in Table S6. The figures of crystallographic models were prepared using PyMol (Molecular Graphic System, Version 2.0, Schrödinger, LLC), while 2D structure diagrams of protein–ligand complexes were generated with PoseView program [38,39]. 

### 4.12. Homology Modelling and Docking Simulations

The initial three-dimensional structures of compounds were prepared in the MOE software (using the QuickPrep tool) [40]. The two cN-IIIB structures were considered as docking receptors: (i) the open crystallographic structure with compound **5d** (obtained in this work) and (ii) the homology model of cN-IIIB built to obtain the closed conformation of the enzyme. The homology model of cN-IIIB (sequence: UniProt code Q969T7) was built using the SwissModel web server [41] on the basis of the crystallographic structure of murine cN-IIIA (PDB id: 4FE3, chain A) [13] as a template. The sequences of human cN-IIIB and mcN-IIIA binding sites (defined as a binding site residue within 5 Å of the compound **5d**’ heavy atoms) are about 77% similar (Figure S10). The resultant model was assessed by SwissModel and further validated with the WHAT_CHECK tool [42] (Figure S10, Table S8). The homology model then underwent further refinement, including fixing the selected residue rotamers. The system was parametrized with the default Amber10:EHT force field [40], and the active site with the m^7^GMP substrate (built on the basis of the original uridine 5’-monophosphate copied from the template) was minimized in MOE (8 Å from the ligand). Both receptors were then prepared with the MOE QuickPrep tool [40], prior to docking simulations. The binding pocket was defined on the basis of the sum of the residues within 5 Å of the ligand heavy atoms in the two receptors considered, with residues 44 and 231 added after visual inspection. Docking simulations were performed with the MOE software [40]. The Triangle Matcher ligand placement method was used together with the London dG scoring (60 poses per ligand were retained), and then the refinement was performed with the Induced-Fit protocol and the GBVI/WSA dG scoring [43], and the 10 output poses per ligand were returned. The Induced-Fit protocol was used, partly to account for the potential inaccuracies of the binding site in the homology model. If not otherwise noted, docking poses were selected on the basis of (i) similarity to the known binding mode (e.g., substrate-like; usually these were the top poses) or (ii) the lowest (most favorable) final docking score. The poses were sorted by docking score, so the "top" pose refers to the one with the lowest final docking score.

## Figures and Tables

**Table 2:** Selectivity of second-generation inhibitors. The selectivity index towards weak binders of eIF4E (SI eIF4E) is expressed as a ratio of the IC_50_ values for cN-IIIB to EC_50_ values for eIF4E, while selectivity index towards weak inhibitors of human cN-IIIA is expressed as ratio of the IC_50_ values for cN-IIIB to IC_50_ values for cN-IIIA.

Compound	EC_50_ µM—eIF4E	SI _(cN-IIIB/eIF4E)_^a^
**m^7^GMP**	8.4 ± 1.7	n.d.
**5**	15.8 ± 2.8	0.62
**5a**	22.6 ± 4.3	0.10
**5d**	39.9 ± 8.5	0.06
**5g**	117.5 ± 66.4	0.06
**Compound**	**IC_50_ µM—cN-IIIA**	**SI _(cN-IIIB/cN-IIIA)_^b^**
**5**	113.4 ± 38.5	0.09
**5a**	126.8 ± 35.6	0.02
**5d**	105.8 ± 26.6	0.02
**5g**	42.5 ± 8.3	0.17

^a^ the ratio of IC_50_ for cN-IIIB and EC_50_ for eIF4E; ^b^ the ratio of IC_50_ for cN-IIIB and IC_50_ for cN-IIIA.

## Data Availability

Not applicable.

## Supplementary Materials

The following supporting information can be downloaded at https://www.mdpi.com/article/10.3390/ph15050554/s1, Figures S1–S10. Tables S1–S8, additional information on the synthetic procedures, and spectroscopic data and HPLC profiles for newly obtained compounds. References [44,45,46,47,48,49,50,51,52,53,54,55,56] are cited in the supplementary materials.

## Accession Codes

The atomic coordinates and structural factors have been deposited in the Protein Data Bank under accession codes 7ZEE (cN-IIIB), 7ZEG (cN-IIIB-**5d**), and 7ZEH (cN-IIIB-**5d’**).
