# Supplementary material for: Substrate-Based Design of Cytosolic Nucleotidase IIIB Inhibitors and Structural Insights into Inhibition Mechanism
Source: Pharmaceuticals (Basel). 2022 Apr 29;15(5):554. doi: 10.3390/ph15050554 (PMC9144445; doi:10.3390/ph15050554)
Supplement: Supplementary file 1 [file pharmaceuticals-15-00554-s001.zip › pharmaceuticals-1685540 revised version for supplementary file (updated header and line number).pdf]

pharmaceuticals

MDPI

Article

Substrate-Based Design of Cytosolic Nucleotidase IIIB Inhibitors and Structural Insights into Inhibition Mechanism

Dorota Kubacka [1,†,‡], Mateusz Kozarski [1,2,†], Marek R. Baranowski [1], Radoslaw Wojcik [2], Joanna Panecka-Hofman [1], Dominika Strzelecka [1,§], Jerome Basquin [3], Jacek Jemielity [2,*] and Joanna Kowalska [1,*]

1 Division of Biophysics, Institute of Experimental Physics, Faculty of Physics, University of Warsaw,Pasteura 5, 02-093 Warsaw, Poland; dkubacka1@gmail.com (D.K.); mateusz.kozarski@fuw.edu.pl (M.K.); marek.baranowski@fuw.edu.pl (M.R.B.); joanna.panecka@uw.edu.pl (J.P.-H.); dominika.strzelecka@student.uw.edu.pl (D.S.)
2 Centre of New Technologies, University of Warsaw, Banacha 2c, 02-097 Warsaw, Poland; r.wojcik7@student.uw.edu.pl
3 Department of Structural Cell Biology, Max-Planck-Institute of Biochemistry, Am Klopferspitz 18, D-82152 Martinsried, Germany; basquin@biochem.mpg.de
* Correspondence: j.jemielity@cent.uw.edu.pl (J.J.); jkowalska@fuw.edu.pl (J.K.); Tel.: +4822-5543-774 (J.K.).
† These authors contributed equally to the work.
‡ Current Address: R&D Centre, Celon Pharma SA, Marymoncka 15 Street, 05-152 Kazu´n Nowy, Poland.
§ Current Address: Institut Européen de Chimie et Biologie, University of Bordeaux, CNRS, INSERM, ARNA, UMR 5320, U1212, IECB, F-33600 Pessac, France.

Supplementary information

Tables………………………………………………………………….. 2

Figures………………………………………………………………… 21

Procedures for the Synthesis of Nucleotides…………………………… 30

Spectroscopic data……………………………………………………… 30

Tables

Table S1. Structures of first-generation inhibitors

Name of compound	Structure
**Natural nucleotidase**	
m^7Guo	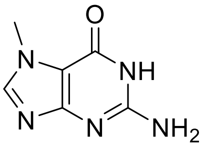
m^7GMP	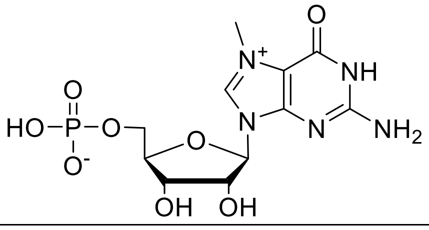
m^7GDP	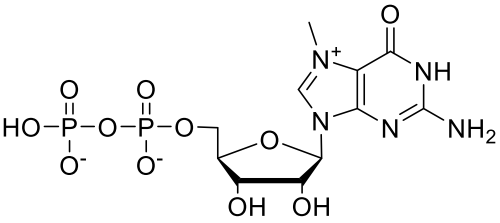
m^7GTP	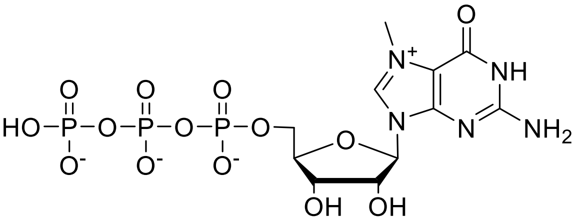
m^7GpppG	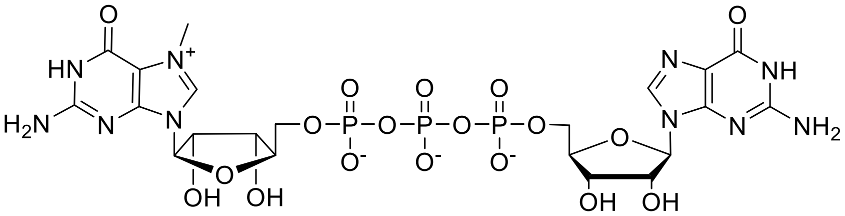
5’-IMP	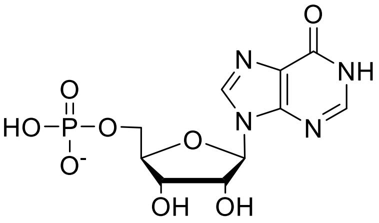
5’-CMP	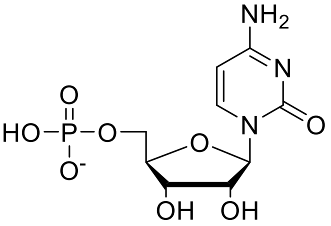

d-CMP	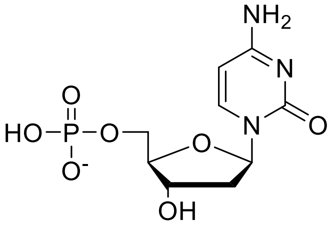
5'-TMP	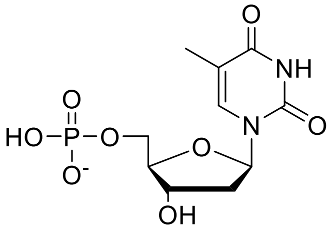
**Phosphate-modified m^{7}GMP and m^{7}GDP**	
m^{7}GMPF **(S1)**	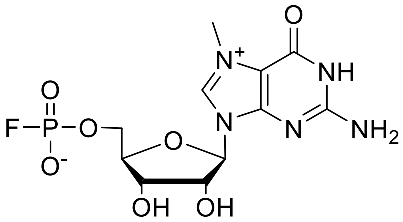
m^{7}GDPF **(S2)**	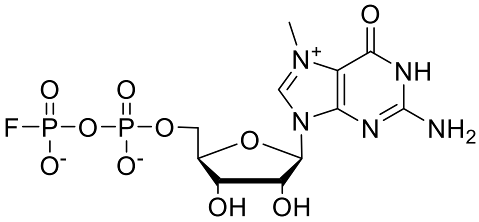
m^{7}GMPS **(S3)**	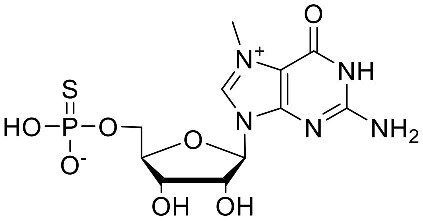
m^{7}GpNHC$_3$H$_3$ **(S4)**	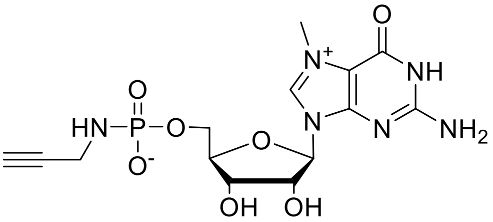
m^{7}GppNHC$_3$H$_3$ **(S5)**	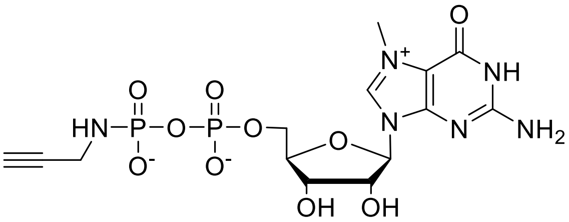

$m^7GpNHC_2H_4N_3$ **(S6)**	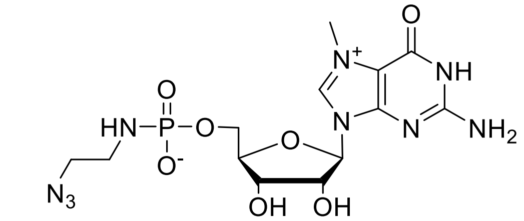
$m^7GppNHC_2H_4N_3$ **(S7)**	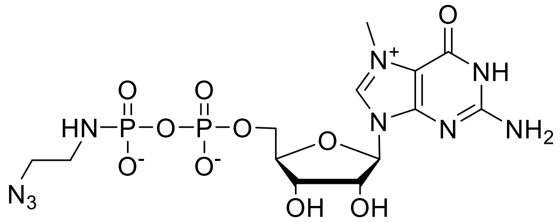
$m^7GppC_4H_5$ **(S8)**	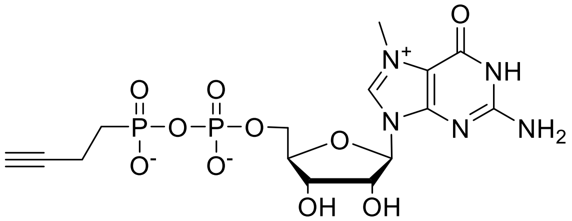
$m^7GppC_3H_3$ **(S9)**	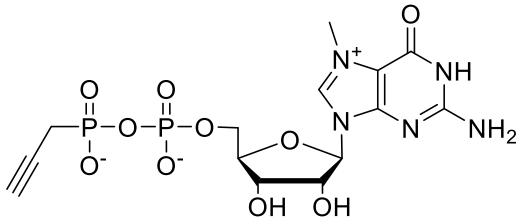
m^7GppC_2H **(S10)**	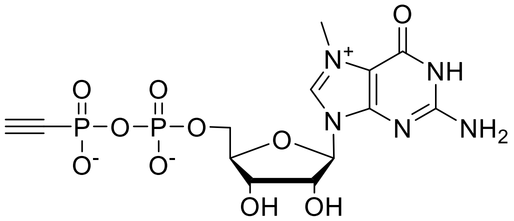
$m^7GppOC_3H_3$ **(S11)**	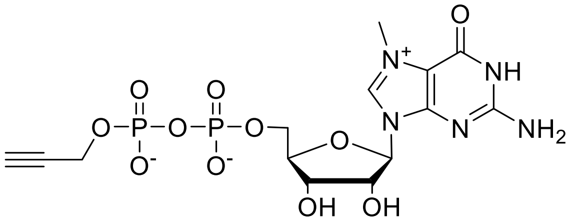
$m^7GMPSNH_2$ D1 **(S12)**	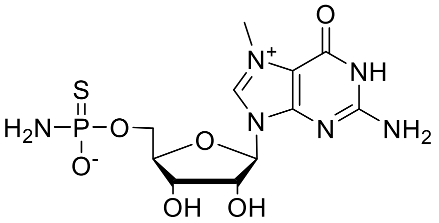
$m^7GMPSNH_2$ D2 **(S13)**	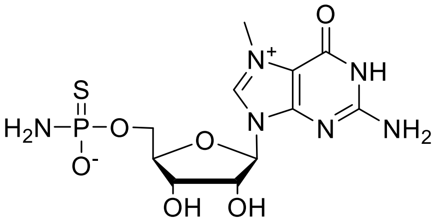

m^{7}G-triazol-P **(S14)**	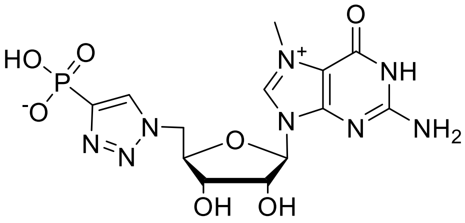
m^{7}G-triazol-CH_2P **(S15)**	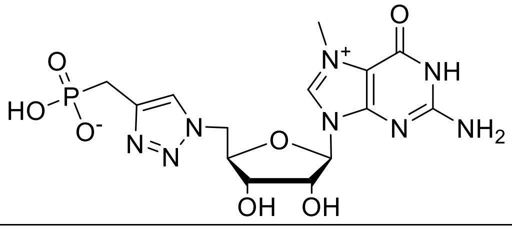
m^{7}G-triazol-C_2H_4P **(S16)**	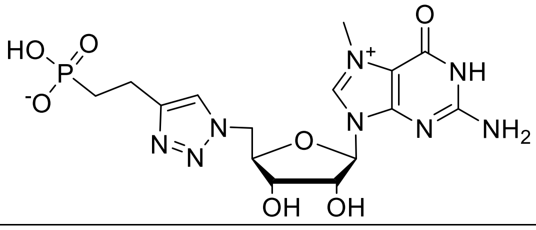
m^{7}G-triazol-OCH_2P **(S17)**	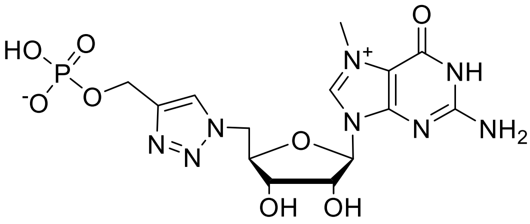
m^7-5'-S-GMP **(S18)**	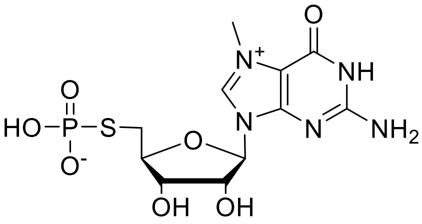
Nucleobase- modified m^{7}GMP & miscellaneous	
bn^{7}GMP **(S19)**	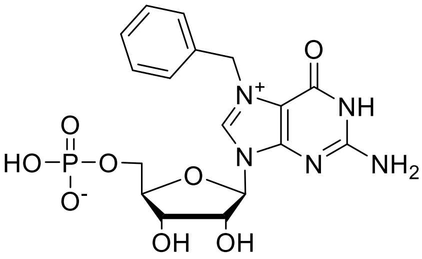
bn^{7}GDP **(S20)**	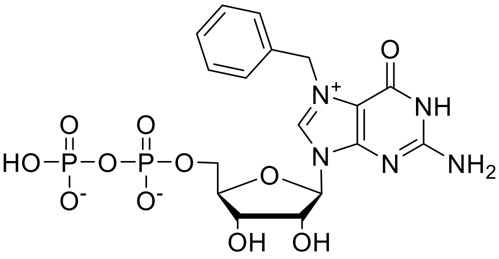

Pr-gyl^{7}GMP **(S21)**	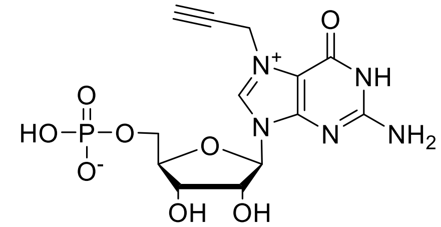
GMPS **(S22)**	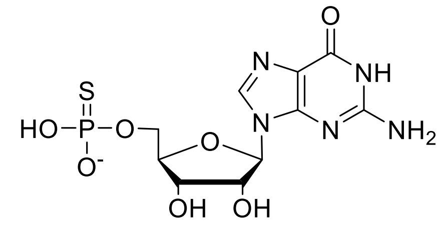
N1-Pr-GMP **(S23)**	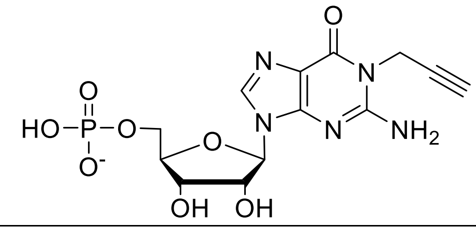
N1-Pr-GMPS **(S24)**	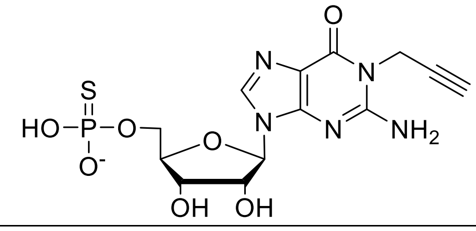
m$^{2'-O}$GMP **(S25)**	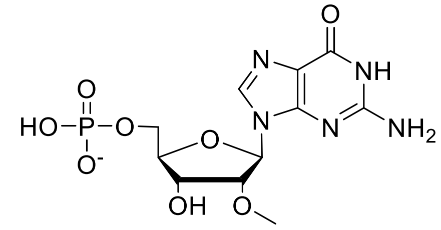
Ribose-modified m^{7}GMP	
m$^{7, 2'-O}$GMP **(S26)**	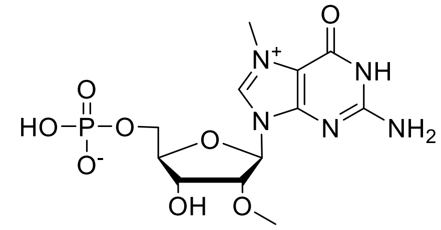
m$^{7,2'-NH2}$GMP **(S27)**	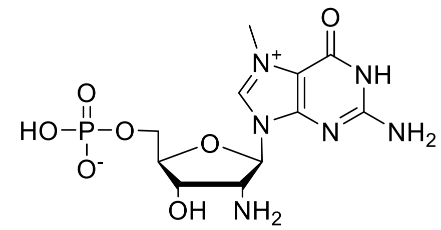

2’,3’-iPr- m^{7}GMP **(S28)**	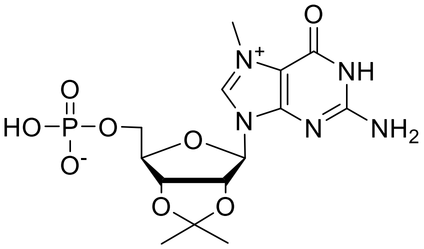
2’-O-L13N-m^{7}GMP **(S29)**	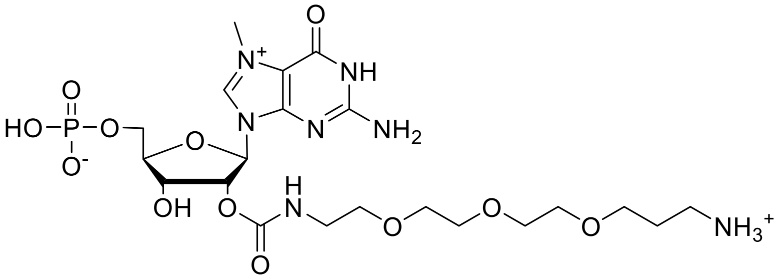
3’-O-L13N-m^{7}GMP **(S30)**	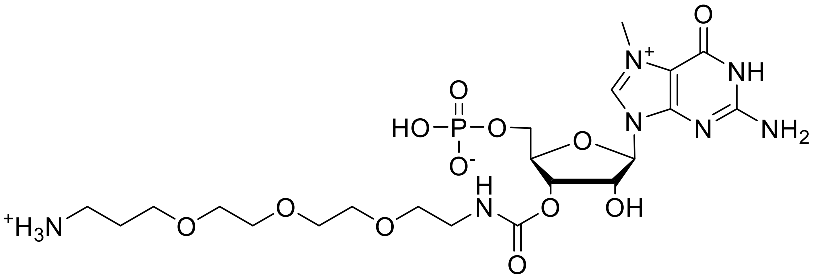
L3C-m^{7}GMP **(S31)**	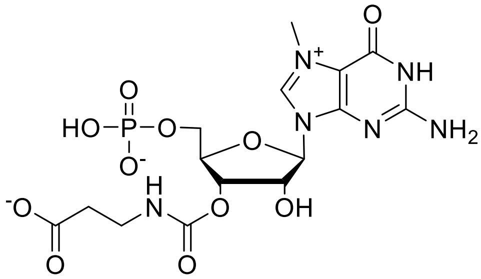
2’-NH-Biot-m^{7}GMP **(S32)**	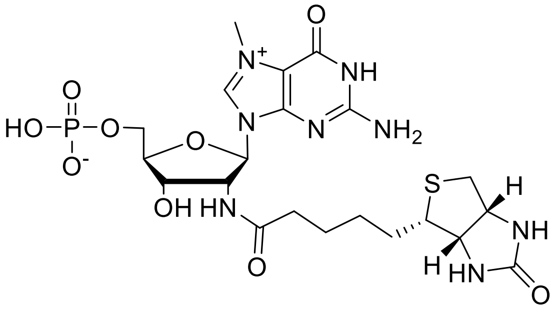

Table S2. Structures of second-generation inhibitors

**Type II- Analogs of compound 4**	
m[7]GtriazolP (isomer 1,5)<br>**4a**	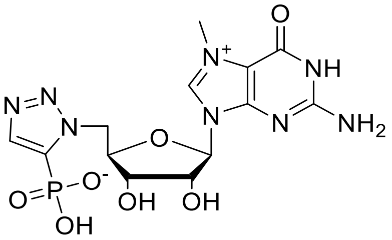
m[7]G-9-triazol-CH_2P<br>**4b**	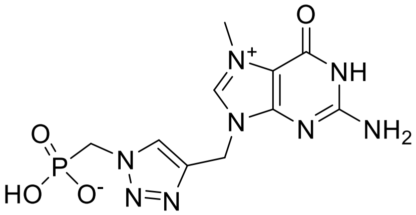

Name of compound	Structure
Type I- Analogs of compound **5**- benzyl derivatives	
3meBn[7]GMP<br>**5a**	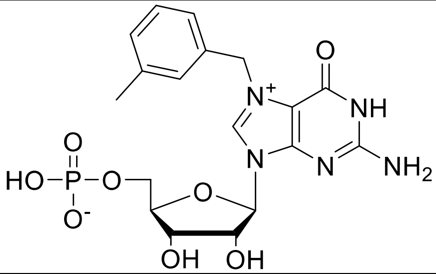
4meBn[7]GMP<br>**5b**	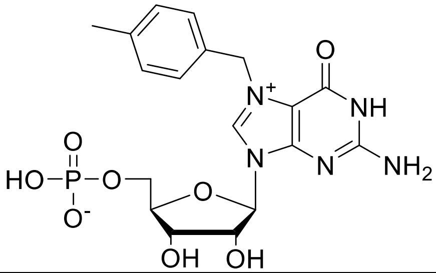
3,5meBn[7]GMP<br>**5c**	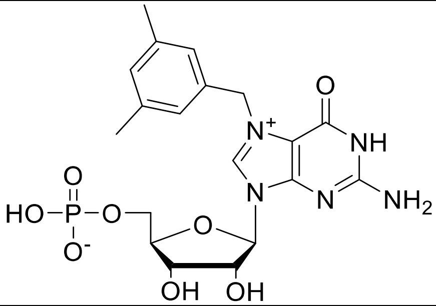
3,4F_2Bn[7]GMP<br>**5d**	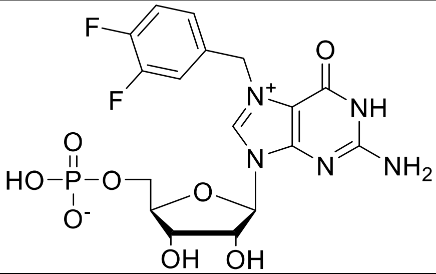

2,4F_2Bn^7GMP<br>**5e**	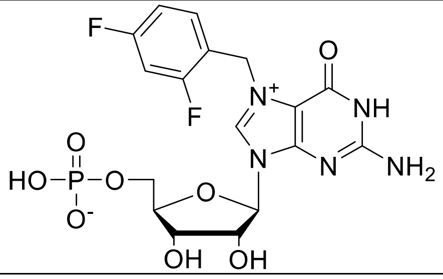
3,4,5F_3Bn^7GMP<br>**5f**	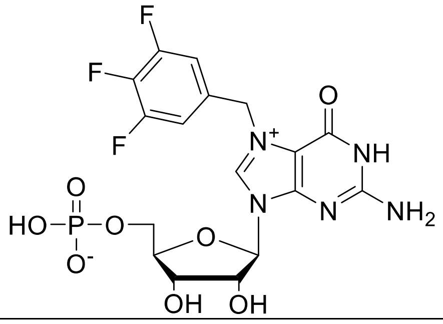
4CF_3Bn^7GMP<br>**5g**	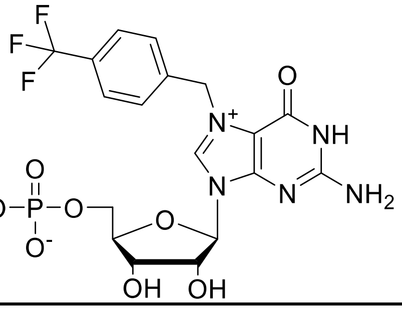

**Type III- Analogs of compound 5 with phosphate modification**	
Bn^7GMPF<br>**8a**	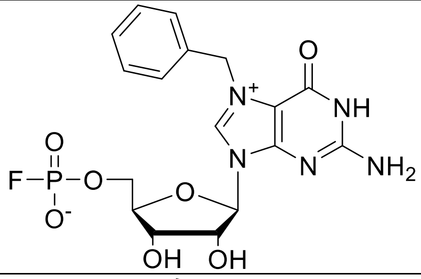
Bn^7GDPF<br>**8b**	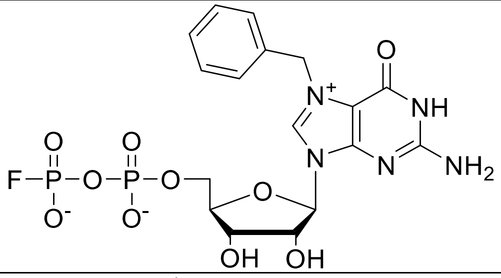
Bn^7GMPH<br>**8c**	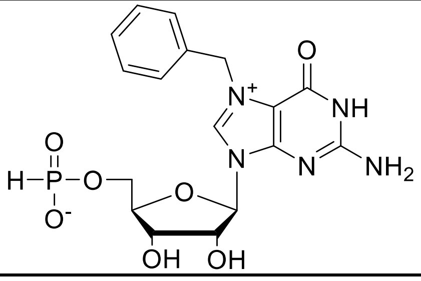

Bn[7]GtriazolP **8d**	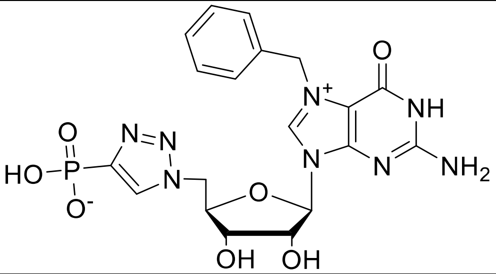
3meBn[7]GtriazolP **8e**	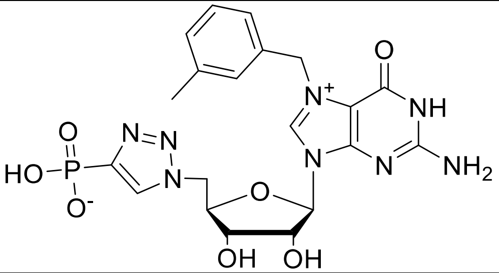

Table S3. The inhibitory potency and hydrolysis of 41 compound of library I.

	Compound	Susceptibility to hydrolysis (%)	Inhibition of m^7GMP hydrolysis (%)
Natural nucleotides	m7Guo	0.0	92.7
	m7GMP	100.0	105.8
	m7GDP	97.8	92.0
	m7GTP	0.0	86.5
	m7GpppG	0.0	101.3
	5'-IMP	24.4	100.2
	5'-CMP	168.9	93.2
	2'-dCMP	104.4	95.6
	5'-TMP	153.3	103.1
	water	0.0	100.0
Phosphate-modified m^7GMP and m^7GDP	S1	0.0	81.9
	S2	0.0	96.4
	S3	0.0	68.3
	S4	0.0	105.5
	S5	0.0	100.9
	S6	0.0	101.0
	S7	0.0	91.7
	S8	0.0	98.7
	S9	0.0	98.4
	S10	0.0	99.0
	S11	0.0	99.2
	S12	0.0	80.5
	S13	0.0	79.6
	S14	0.0	34.9
	S15	0.0	98.9
	S16	0.0	102.5
	S17	122.2	99.0
	S18	0.0	52.0
Nucleobase-modified & miscellaneous	S19	0.0	23.2
	S20	0.0	57.8
	S21	0.0	37.7
	S22	0.0	105.2
	S23	73.3	100.5
	S24	0.0	91.9
	S25	0.0	90.0
	DMSO	0.0	106.0
Ribose-modified m^7GP	S26	60.0	100.5
	S27	57.8	99.3
	S28	33.3	97.6
	S29	0.0	96.8
	S30	0.0	105.1
	S31	20.0	82.9
	S32	0.0	116.5

Table S4. The inhibitory potency and hydrolysis of compounds of library II.

	Compound	Susceptibility to hydrolysis (%)	Inhibition of m^7GMP hydrolysis (%)
Hits from the first screening	water	0.0	100.0
	1	0.0	84.8
	2	0.0	52.0
	3	0.0	57.6
	4	0.0	52.3
	5	0.0	7.8
	6	0.0	24.8
The second- generation inhibitors	4a	0.0	20.4
	4b	0.0	64.8
	DMSO	0.0	111.7
	5a	0.0	9.7
	5b	0.0	26.1
	5c	0.0	18.5
	5d	0.0	1.7
	5e	0.0	46.6
	5f	0.0	30.9
	5g	0.0	16.6
	8a	0.0	94.6
	8b	0.0	48.6
	8c	0.0	89.1
	8d	0.0	58.7
	8e	0.0	35.7

Table S5. Selectivity of second-generation inhibitors towards eIF4E and cN-IIIA

Compound	EC_{50} ± SEM (µM)	SI $_{(cN\text{-}IIIB/eIF4E)}$
	**Selectivity cN-IIIB/eIF4E**	
**m^7GMP**	8.4 ± 1.7	-
**5**	15.8 ± 2.8	0.62
**5a**	22.6 ± 4.3	0.10
**5d**	39.9 ± 8.5	0.06
**5g**	117.5± 66.4	0.06

Compound	IC_{50} ± SEM (µM)	SI $_{(cN\text{-}IIIB/cN\text{-}IIIA)}$
	**Selectivity cN-IIIB/cN-IIIA**	
**5**	113.4 ± 38.5	0.09
**5a**	126.8 ± 35.6	0.02
**5d**	105.8 ± 26.6	0.02
**5g**	42.5 ± 8.3	0.17

Table S6. Data collection and refinement statistics for crystal structures.

	cN-IIIB • Mg^{2+}	cN-IIIB • Mg^{2+} • 5d	cN-IIIB • Mg^{2+} • 5d'
PDB code	7ZEE	7ZEG	7ZEH
Wavelength (Å)	0.979	0.918	1
Resolution range (Å)	38.24 - 1.36<br>(1.41 - 1.36)	40.69 - 1.56<br>(1.61 - 1.56)	75.08 - 1.5<br>(1.55 - 1.5)
Space group	P 21 21 21	P 1 21 1	P 1
Cell dimensions	41.7 79.3 87.3<br>90 90 90	40.8 79.1 94.4<br>90 93.2 90	60.6 80.9 83.2<br>82.2 72.5 68.2
Total reflections	717512 (23860)	562734 (52791)	2135072 (172959)
Unique reflections	60466 (4228)	85124 (8109)	218378 (20240)
Multiplicity	11.9 (5.6)	6.6 (6.4)	9.8 (8.5)
Completeness (%)	95.2 (67.3)	99.2 (95.7)	97.5 (90.3)
Mean I/sigma(I)	25.8 (1.7)	10.7 (0.7)	15.4 (1.6)
Wilson B-factor ($Å^2$)	19.4	24.0	19.3
R-merge	0.052 (0.840)	0.091 (2.499)	0.083 (1.393)
R-meas	0.055 (0.919)	0.098 (2.720)	0.088 (1.481)
$CC_{1/2}$	0.999 (0.824)	0.999 (0.439)	0.999 (0.732)
CC*	1 (0.951)	1 (0.781)	1 (0.919)
Reflections used in refinement	60444 (4224)	84907 (8102)	218319 (20233)
Reflections used for R-free	3023 (212)	3129 (298)	10917 (1011)
R-work	0.159 (0.308)	0.240 (0.434)	0.188 (0.331)
R-free	0.197 (0.335)	0.273 (0.462)	0.220 (0.350)
CC (work)	0.955 (0.893)	0.934 (0.672)	0.947 (0.852)
CC (free)	0.936 (0.892)	0.919 (0.732)	0.929 (0.808)
Number of non-hydrogen atoms	2642	4846	10071
macromolecules	2282	4372	8821
ligands	65	88	196
solvent	295	386	1054
Protein residues	282	558	1104
RMS (bonds) (Å)	0.012	0.003	0.013
RMS (angles) (deg)	1.22	0.57	1.22
Ramachandran favored (%)	98.6	98.4	98.3
Ramachandran allowed (%)	1.4	1.6	1.8
Ramachandran outliers (%)	0	0	0
Rotamer outliers (%)	0	0	0
Clashscore	3.0	3.9	2.5
Average B-factor	28.6	37.9	28.7
macromolecules	26.4	37.6	27.3
ligands	48.9	32.9	43.6
solvent	40.8	42.2	38.1

Statistics for the highest-resolution shell are shown in parentheses.

Table S7. The docking results to the closed and open cN-IIIB receptor for the selected compounds discussed in the text: natural ligands and the m^7GMP N7-substituted derivatives. The poses are classified depending on the position of m^7guanosine in the pocket: “+” – substrate-like binding mode, “x” – the other binding mode. The data for the top docking pose (with the best score) and the pose with the substrate-like binding mode (if not the first one and if it is found among the 10 best docking solutions) are always shown. The poses are ranked by the docking score. The description of the closed and open receptors is provided in *Methods*.

	open receptor			closed receptor		
**Compound**	**Pose no.**	**Score**	**Pose type**	**Pose no.**	**Score**	**Pose type**
m^7GMP	1	-8.48	+	1	-13.91	+
m^7Gua	1	-7.33	x	1	-8.31	x
	6	-6.62	+	3	-8.16	+
m^7GDP	1	-9.48	+	1	-13.38	+
5	1	-9.05	+	1	-13.44	x
5a	1	-8.97	+	1	-13.86	x
5b	1	-7.87	x	1	-14.19	x
5c	1	-8.22	x	1	-14.08	x
5d	1	-9.12	+	1	-13.69	x
5e	1	-8.88	+	1	-13.37	x
5f	1	-9.78	+	1	-13.39	x
5g	1	-8.34	x	1	-13.90	x

Table S8. The WHAT_IF web server structure validation results for the cN-IIIB “closed” model and the template (PDB: 4FE3). Positive structure Z-scores are better than average, while optimal RMS Z-scores should be close to 1.0

**Structure Z-scores**	**Model**	**Template**
1st generation packing quality	0.198	0.688
2nd generation packing quality	0.045	-0.082
Ramachandran plot appearance	0.6	-0.572
Chi-1/Chi-2 rotamer normality	2.807	-0.815
Backbone conformation	0.488	0.794
RMS Z-scores		
Bond lengths	0.724	0.572 (tight)
Bond angles	1.134	0.683
Omega angle restraints	1.107	1.061
Side chain planarity	0.917	0.326 (tight)
Improper dihedral distribution	1.164	1.02
Inside/Outside distribution	0.993	0.983
B- factor distribution	0.367	1.197

Figures

Bn^{7}GMP derivatives

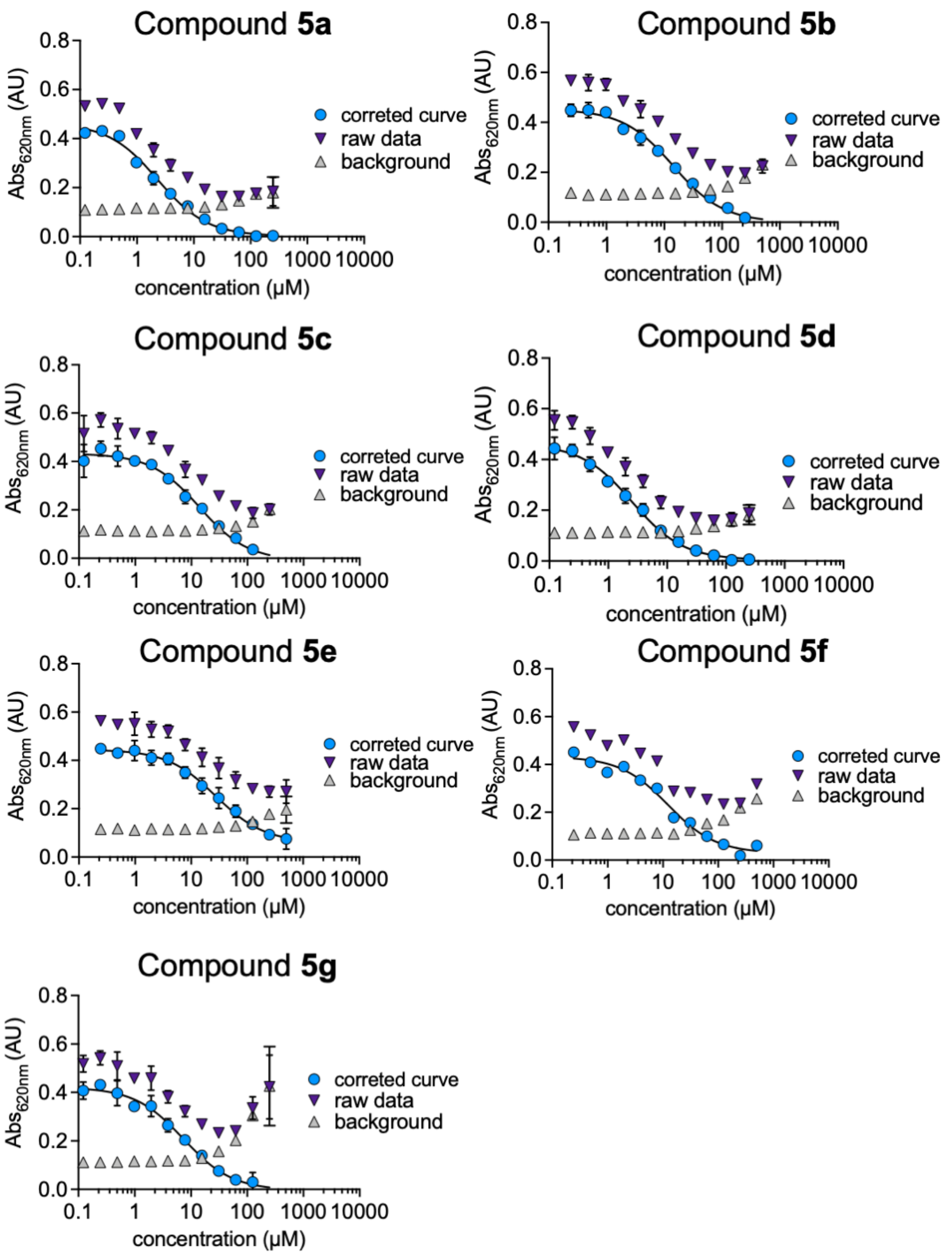

Analogs of compound **4**

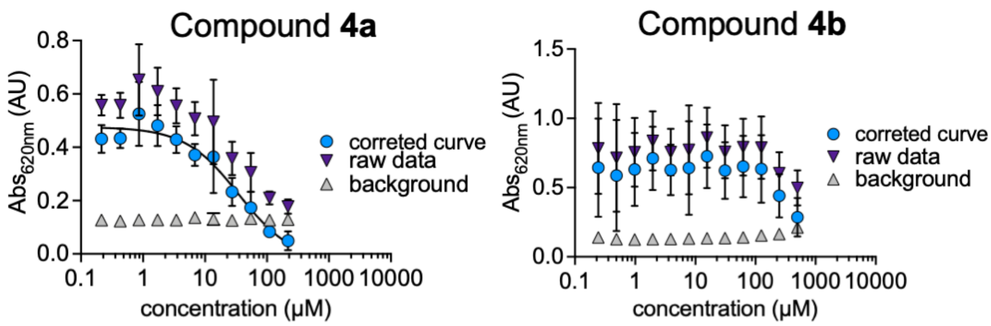

Combination of base and phosphate modifications

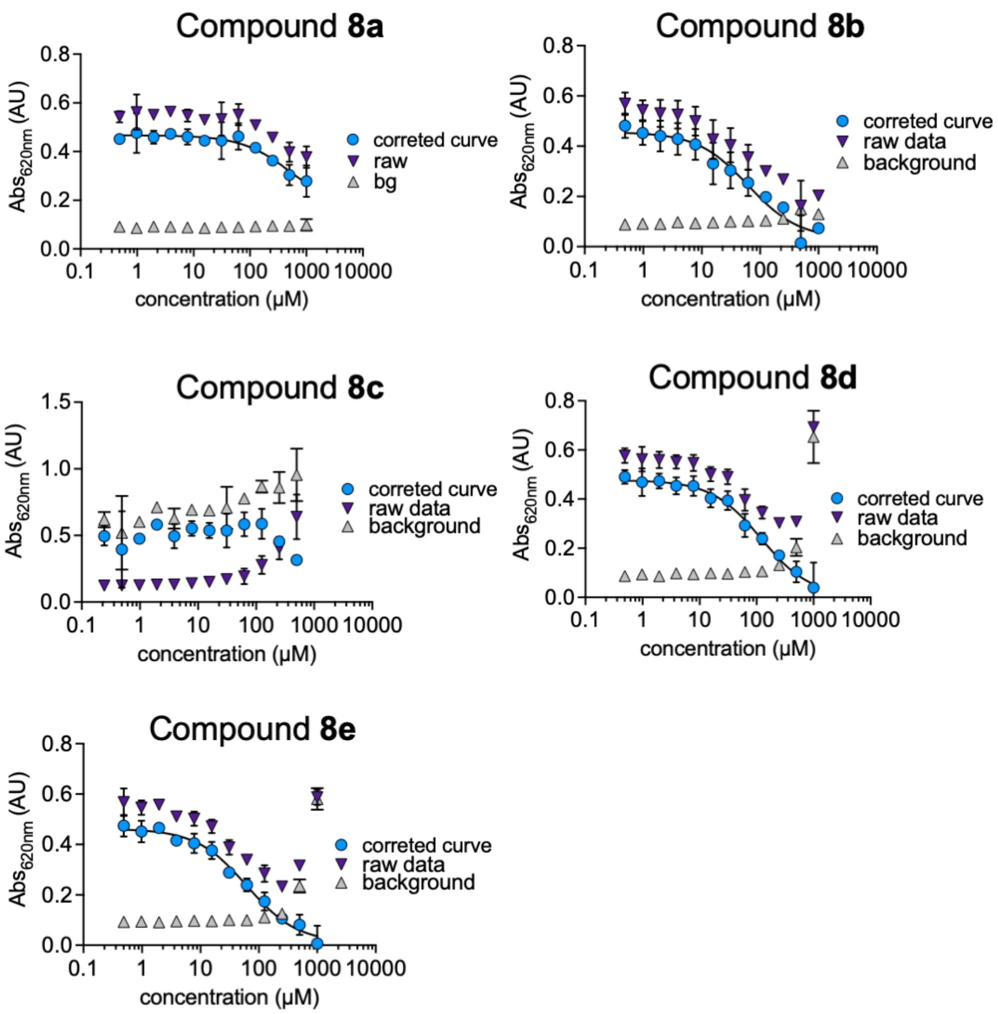

Figure S1. The cN-IIIB inhibition curves determined for the compounds from second library. The IC50 values were determined using MGP assay. The data presents mean values ± SD from triple experiments. To determine the IC50 values, a standard three parameter dose-response equation was fitted to the data.

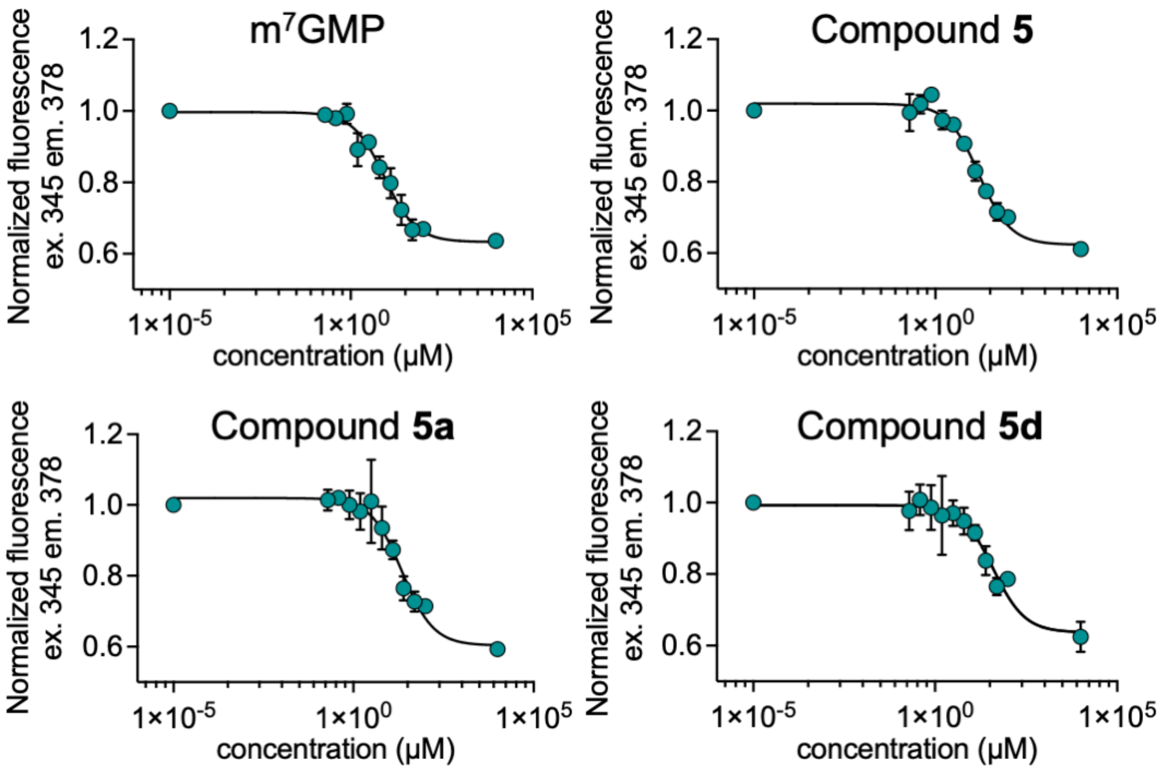

Figure S2. Binding curves for select compounds obtained in the eIF4E competition binding assay using pyrene-labelled m^{7}GTP as a probe. The data presents mean values ± SD from triple experiments.

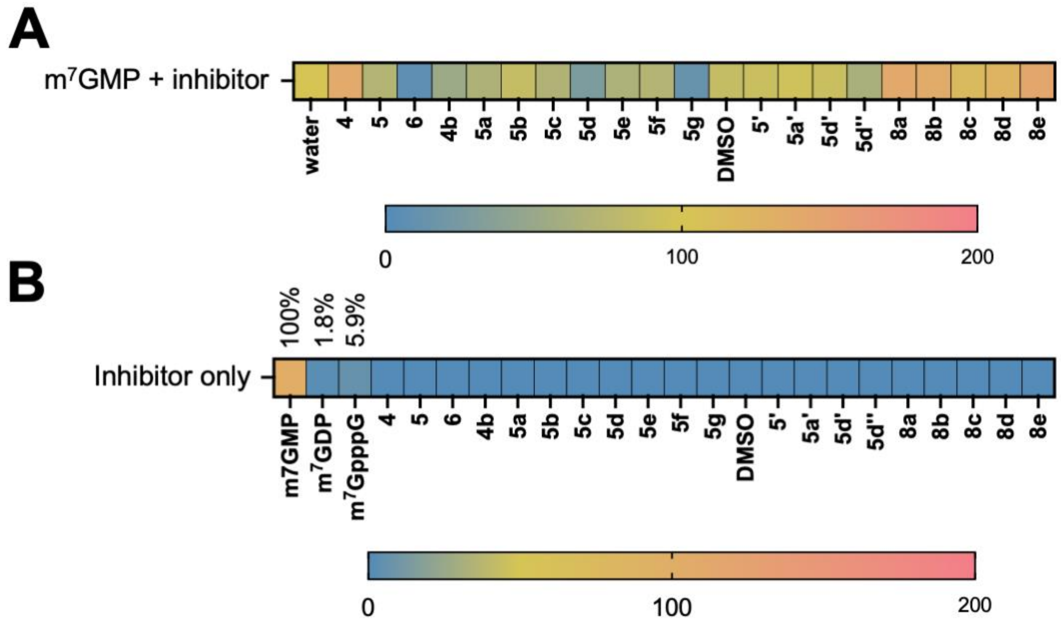

Figure S3. Evaluation of the second-generation inhibitors towards cN-IIIA enzyme. A. Inhibitory potency of compounds of library II which includes 3 leading compounds form first library; B. Evaluation of susceptibility to cN-IIIA for compounds from the second-generation inhibitors library in addition to three leading compounds form the first library and three natural nucleotides.

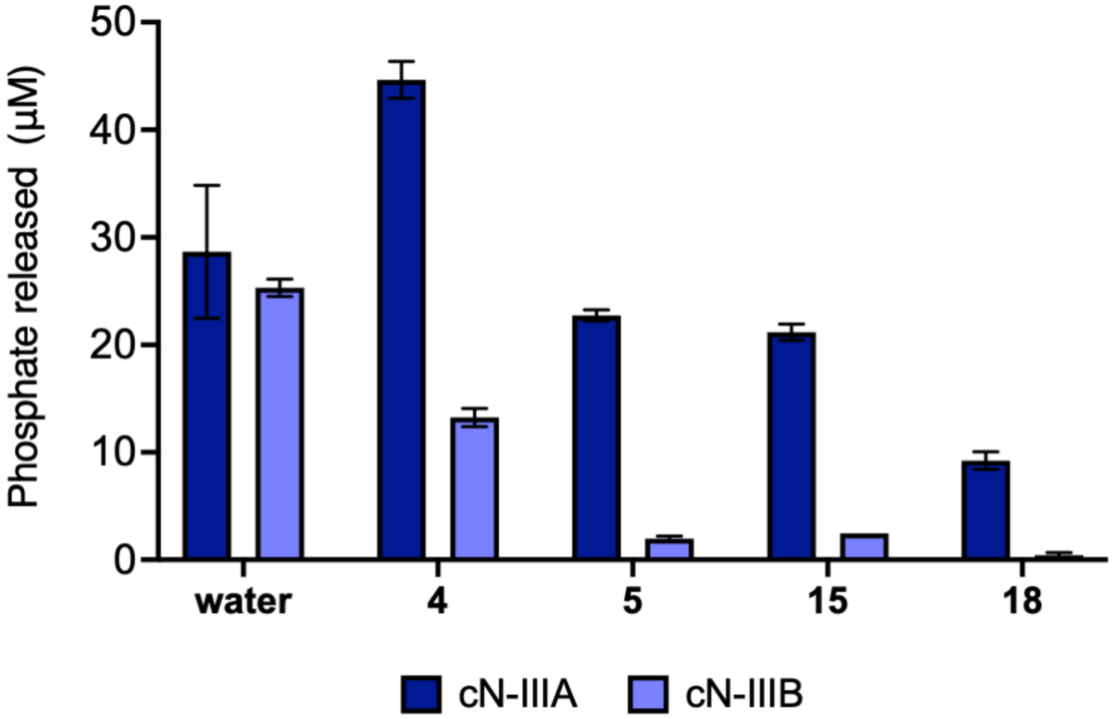

Figure S4. Comparison of inhibitory potency of first- and second- generation inhibitors towards cN-IIIA and cN-IIIB enzyme. The inhibitory potency was determined using MGP assay. Inhibitory potency was introduced as the % of m7GMP - substrate dephosphorylation by cN-IIIA and cN-IIIB with presence of inhibitors. The data represents mean values ± SD from triplicate.

Type I- Analogs of compound **5**- benzyl derivatives

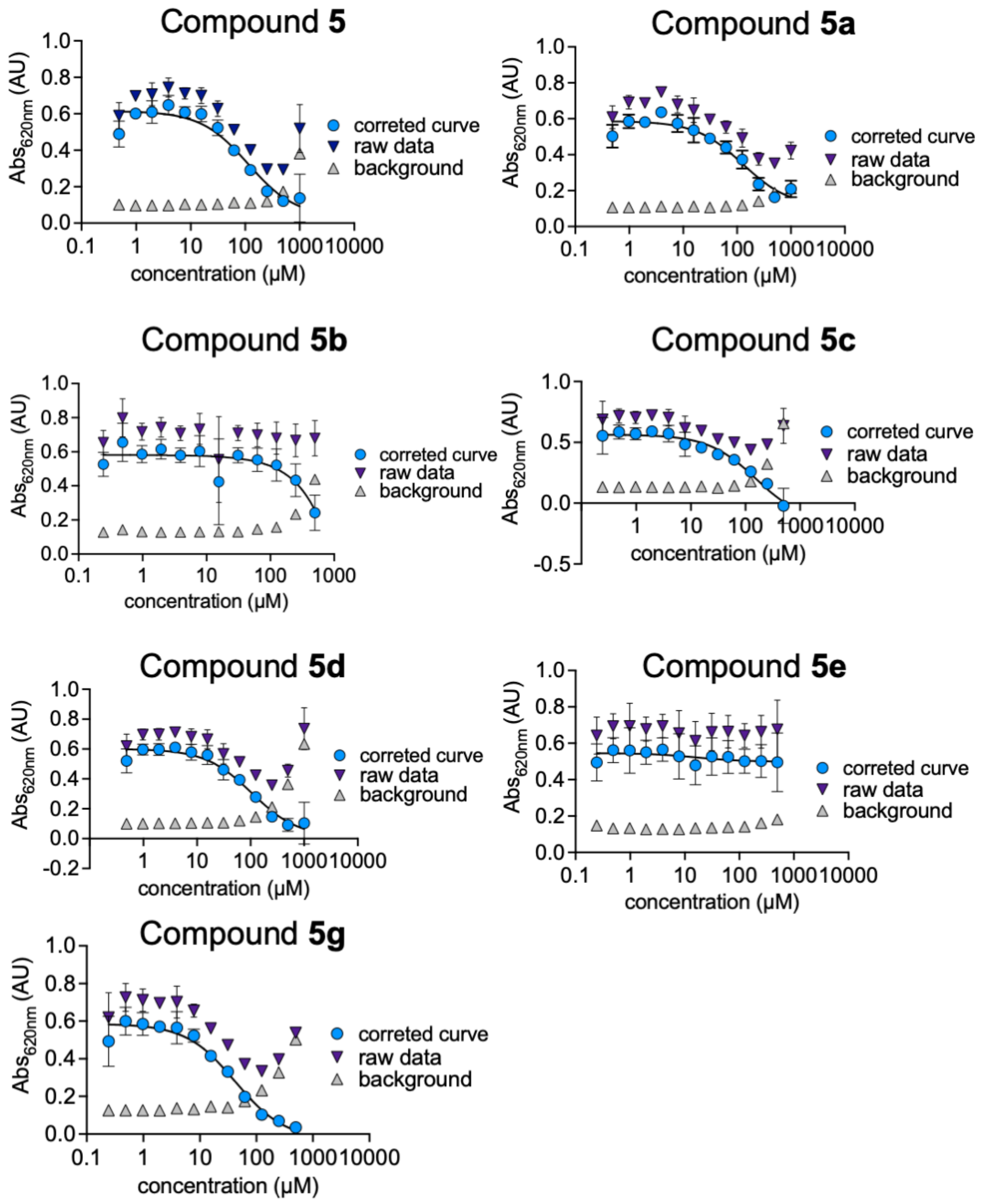

Figure S5. The IC_{50} curves for select compounds determined for cN-IIIA enzyme. The IC50 values were determined using MGP assay. The data presents mean values ± SD from triple experiments. To determine the IC50 values, a standard three parameter dose-response equation was fitted to the data.

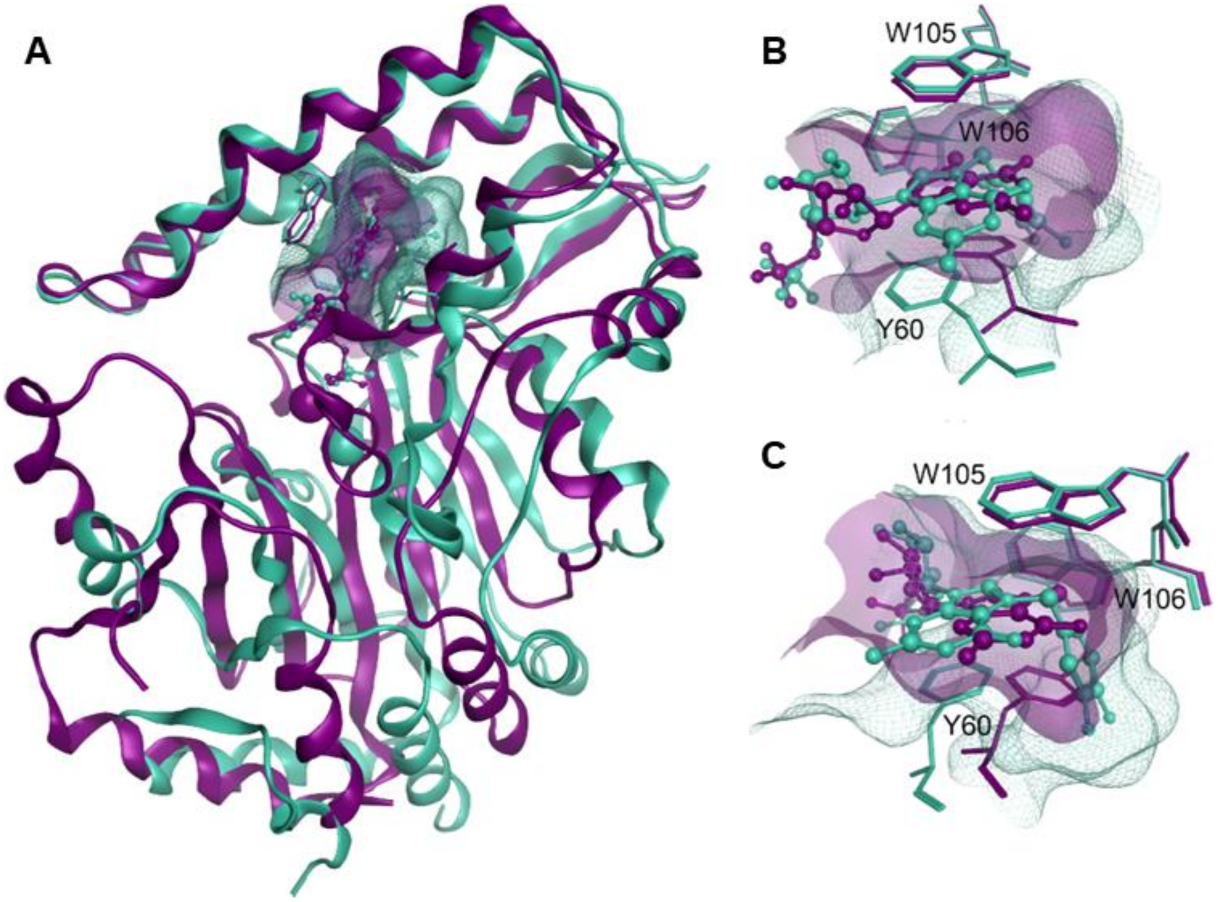

Figure S6. Comparison of cN-IIIB in the closed and open conformations: (a) full closed and open enzyme structures, and two views on the pocket in (b) and (c). The open hcN-IIIB structure in the complex with 3,4-dF-Bn⁷GMP (**5d**) is shown in cyan, whereas the homology model of the closed enzyme form in the complex with the docked m⁷GMP (pose 1) is displayed in purple. Pocket surfaces are drawn within 4.5 ˚AÅ of the ligand fragments (m⁷Gua or m⁷Gua for the closed and open receptors, respectively). Mg^{2+} ions are shown as spheres. The receptors are aligned based on residues 71–115.

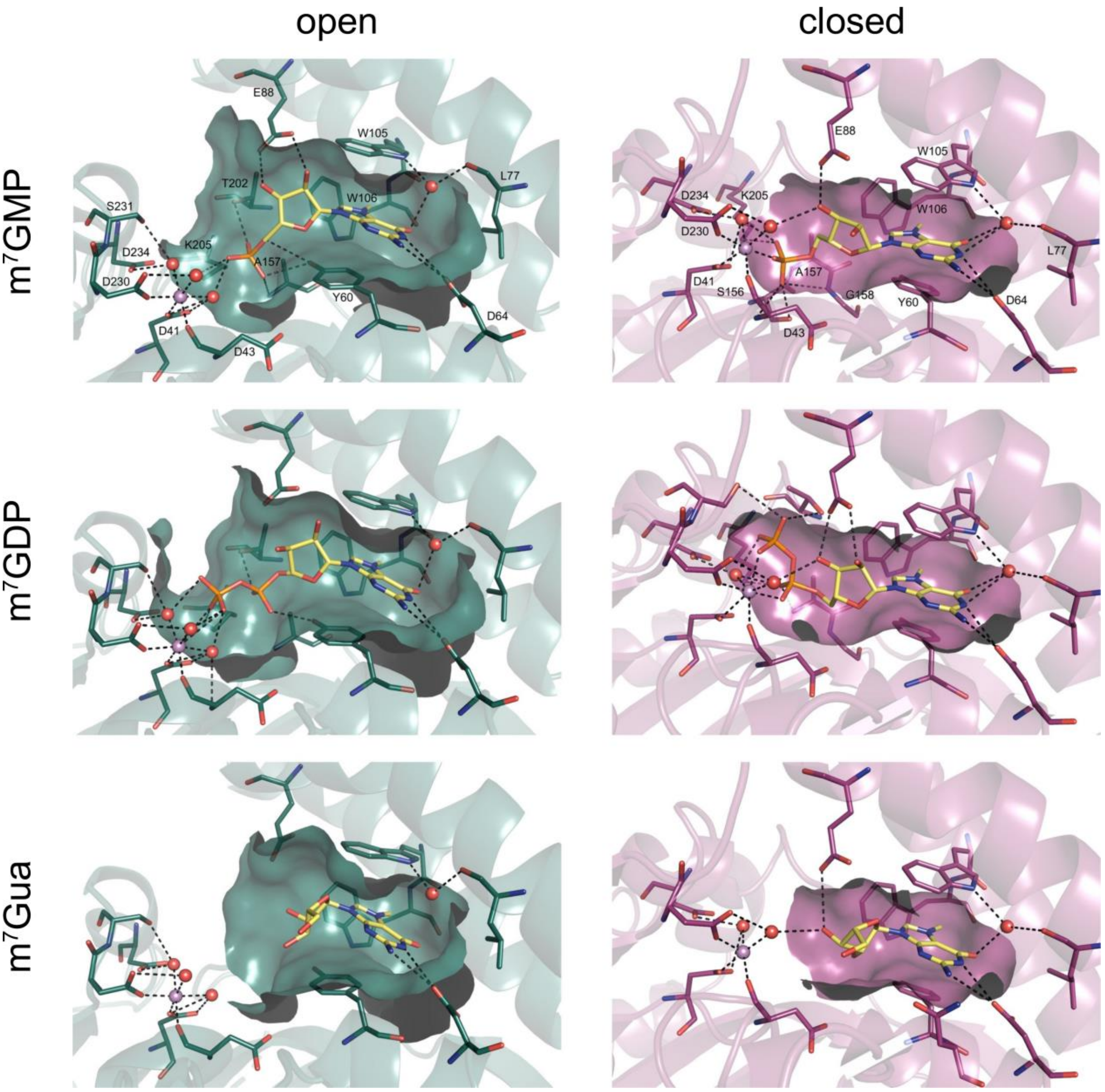

Figure S7. Docking results to the open and closed receptor. For substrates m^{7}GMP and m^{7}GDP top poses are shown. For the product, m^{7}Guo the lowest-score poses that adopt substrate-like binding mode: pose 6 and pose 3 are shown for the open and closed receptor, respectively. The Mg^{2+} ion is represented as a pink sphere, waters - as red spheres. Hydrogens are not shown, all hydrogen bonds of the ligand, hydrogen bonds of waters with the shown receptor residues, and interactions of Mg^{2+} ion are displayed as dashed lines.

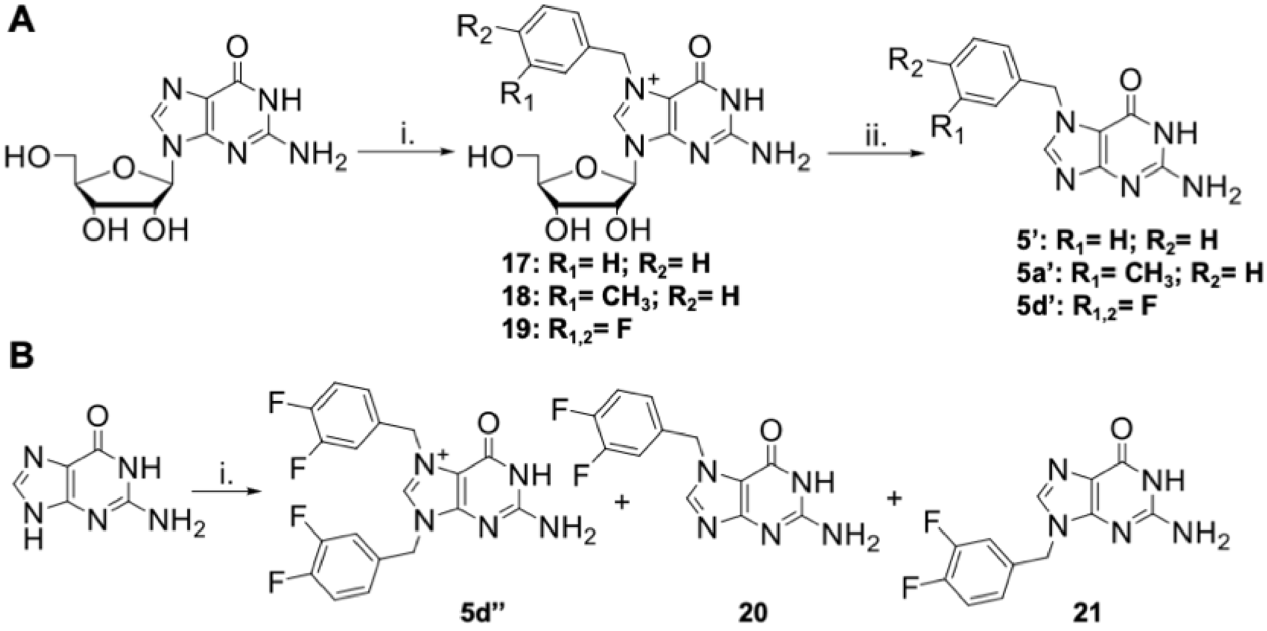

Figure S8. Scheme of synthesis of N7 benzyl guanine analogs. A. Synthesis of monosubstituted benzyl-guanine derivatives (**5′, 5a′** and **5d′**). i. benzyl bromide (**17**) or 3-methyl benzyl bromide (**18)** or 3,4-(difluoro) -benzyl bromide (**19**) - 1.1 equivalents, DMSO, rt, 24 hours; ii. 0.1 M HCl, 70 °C, 2 hours; B. Synthesis of disubstituted benzyl-guanine derivatives (**5d′′**); i. 3,4-(difluoro)- benzyl bromide 6 equivalents, 50 °C, 24 hours[1].

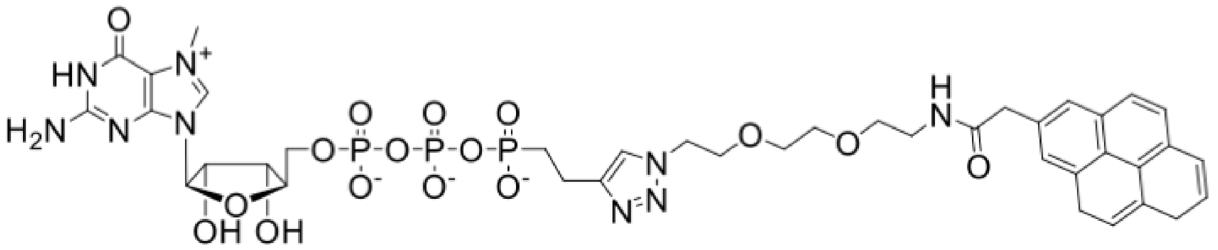

Figure S9. Structure of m7GTP pyrene-labeled probe applied for assessment the specificity of cN-IIIB inhibitors towards eIF4E protein[2].

Model #01	File	Built with	Oligo-State	Ligands	GMQE	QMEAN
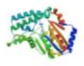	PDB	ProMod3 2.0.0	monomer	None	0.78	-0.77

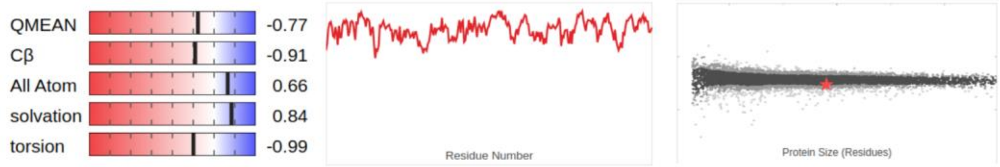

Template	Seq Identity	Oligo-state	QSQE	Found by	Method	Resolution	Seq Similarity	Range	Coverage	Description
4fe3.1.A	57.50	monomer	0.00	BLAST	X-ray	1.74Å	0.47	10 - 289	0.93	Cytosolic 5'-nucleotidase 3

Excluded ligands

Ligand Name.Number	Reason for Exclusion	Description
BME.1	Not biologically relevant.	BETA-MERCAPTOETHANOL
BME.2	Not biologically relevant.	BETA-MERCAPTOETHANOL
MG.4	Binding site not conserved.	MAGNESIUM ION
NA.3	Not biologically relevant.	SODIUM ION
U5P.5	Binding site not conserved.	URIDINE-5'-MONOPHOSPHATE

```
Target     MAEEVSTLMKATVLMRQPGRVQEIVGALRKGGGDRLQVISDFDMTLSRFAYNGKRCPSSYNILDNSKIISEECRKELTAL
4fe3.1.A   ---------KSSVRIKNPTRVEEIICGLIKGGAAKLQIITDFNMTLSRFSYNGKRCPTCHNIIDNCKLVTDECRRKLLQL

Target     LHHYYPIEIDPHRTVKEKLPHMVEWWTKAHNLLCQQKIQKFQIAQVVRESNAMLREGYKTFFNTLYHNNIPLFIFSAGIG
4fe3.1.A   KEQYYAIEVDPVLTVEEKFPYMVEWYTKSHGLLIEQGIPKAKLKEIVADSDVMLKEGYENFFGKLQQHGIPVFIFSAGIG

Target     DILEEIIRQMKVFHPNIHIVSNYMDFNEDGFLQGFKGQLIHTYNKNSSACENSGYFQQLEGKTNVILLGDSIGDLTMADG
4fe3.1.A   DVLEEVIRQAGVYHSNVKVVSNFMDFDENGVLKGFKGELIHVFNKHDGALKNTDYFSQLKDNSNIILLGDSQGDLRMADG

Target     VPGVQNILKIGFLNDKVEERRERYMDSYDIVLEKDETLDVVNGLLQHILCQGVQLEMQGP
4fe3.1.A   VANVEHILKIGYLNDRVDELLEKYMDSYDIVLVKEESLEVVNSILQKTL-----------
```

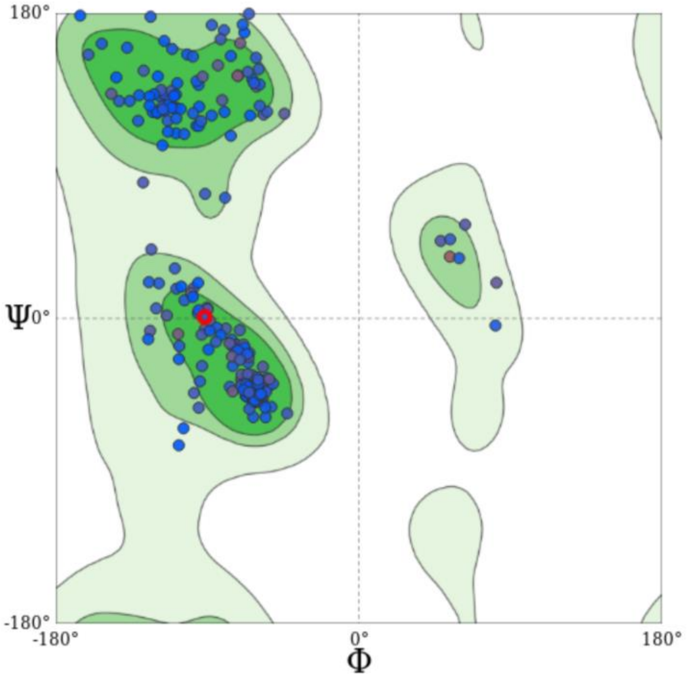

Figure S10. SWISS-MODEL summary report for the human cN-IIIB homology model based on the murine cN-IIIA template (PDB code: 4FE3) and the Ramachandran plot for the model.

Procedures for the Synthesis of Nucleotides

First library of inhibitors

The nucleotides **S1** and **S2**[3]; **S3** and **S22**[4]; **S18**[5]; **S12** and **S13**; **S4-S11**[6-8]; **S14- S17**[8, 9]; **S23** and **S24**[10]; **S25** and **S26**[11] **S27**[12]; **S28**[13]; **S29- S31**[14]; **S32**[15] were synthesized by previously reported procedures [1-17].

Synthesis of substrates used for synthesis of second-generation inhibitors

5′-Deoxy-5′-azidoguanosine[16], guanosine 5′-fluoromono- and diphosphates [3], guanosine 5′-H-phosphonate [17], 7-mehtyl-9-propargylguanine, ethynyl phosphonate[6], azidomethyl phosphonate were synthesized according to previously reported procedures.

Spectroscopic data

**10**, G-triazolP (isomer 1,5)	
^1H NMR	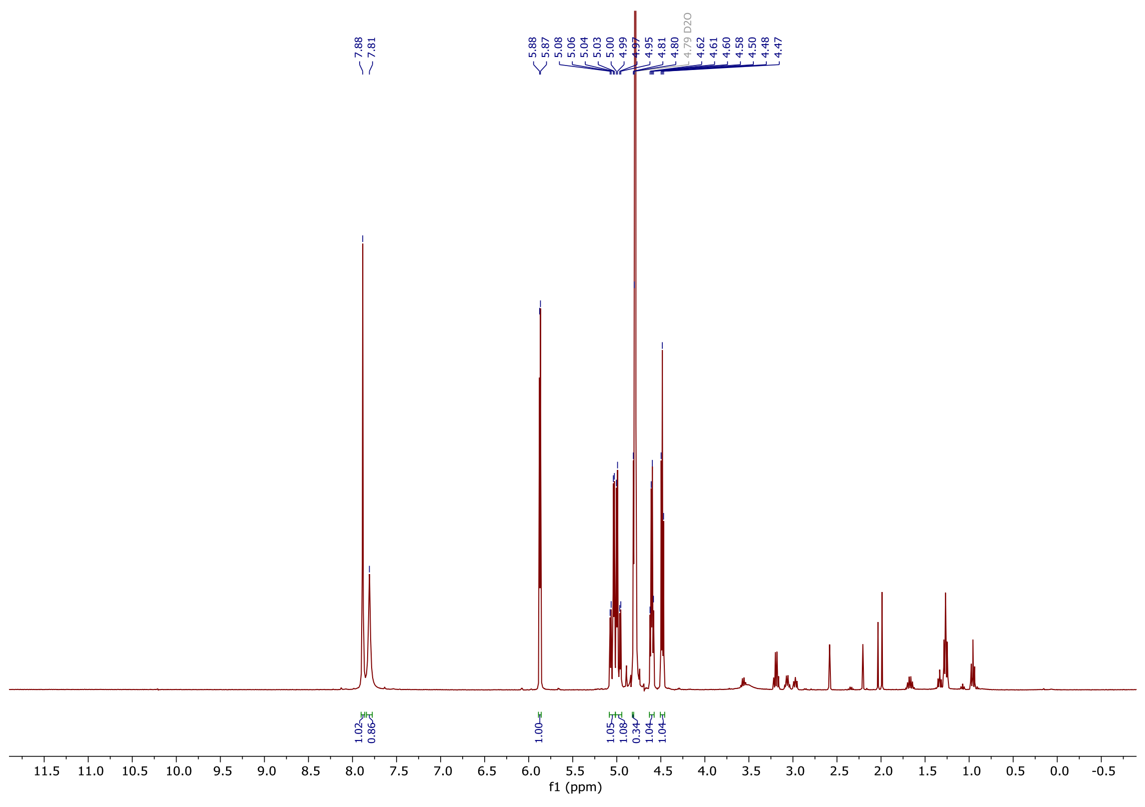

^{31}P NMR	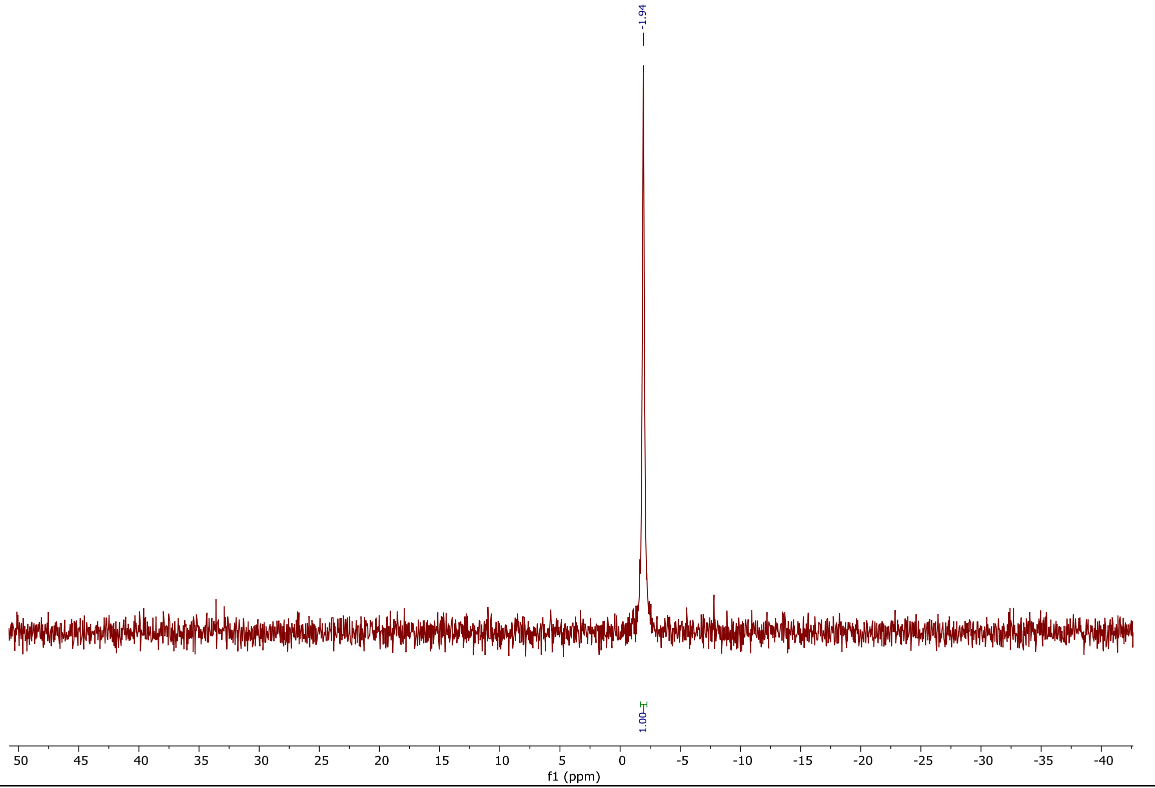
^{01}H- ^{1}H COSY NMR	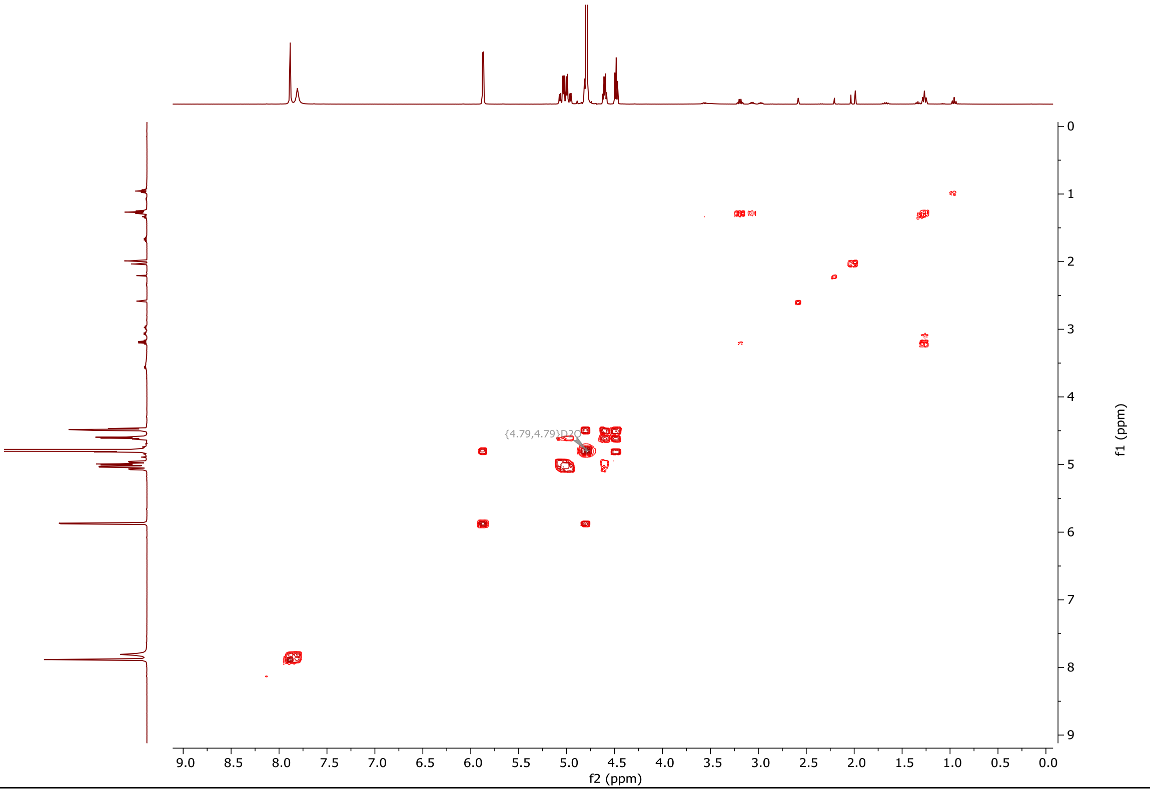

^{13}C- ^{1}H HSQC NMR

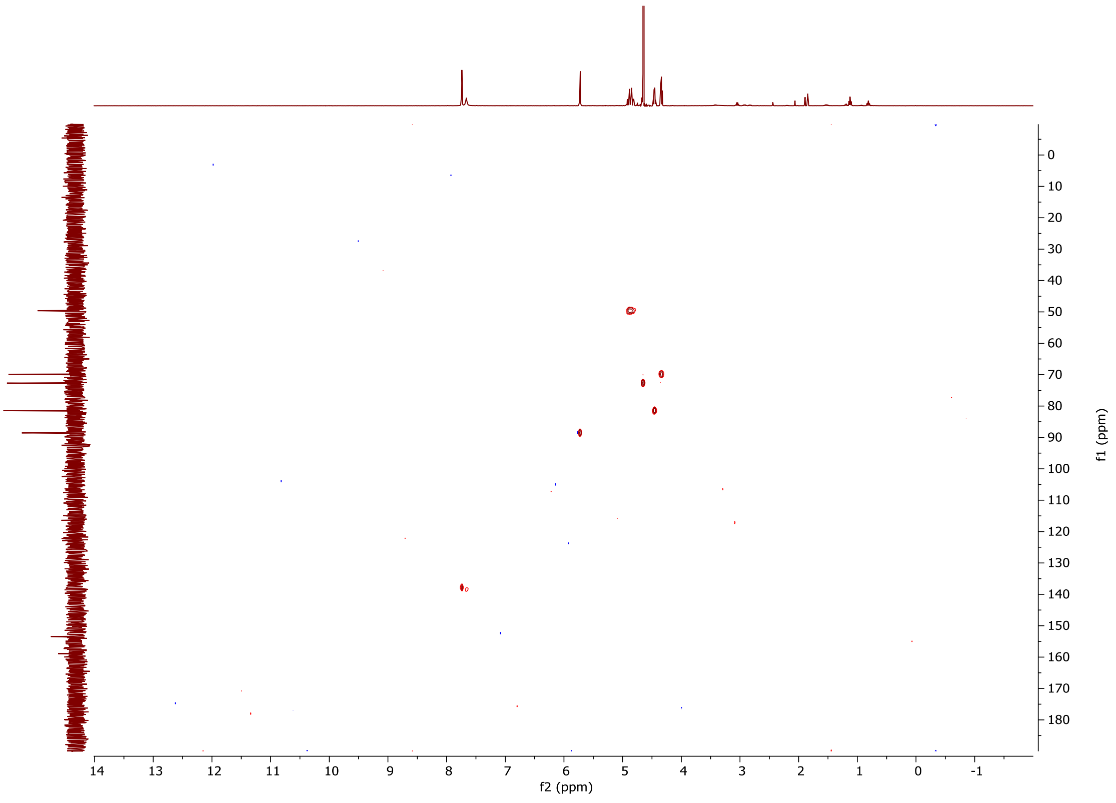

15, (5’-azido-7-benzylguanosine)

RP HPLC profile (Abs, AU, 254 nm)

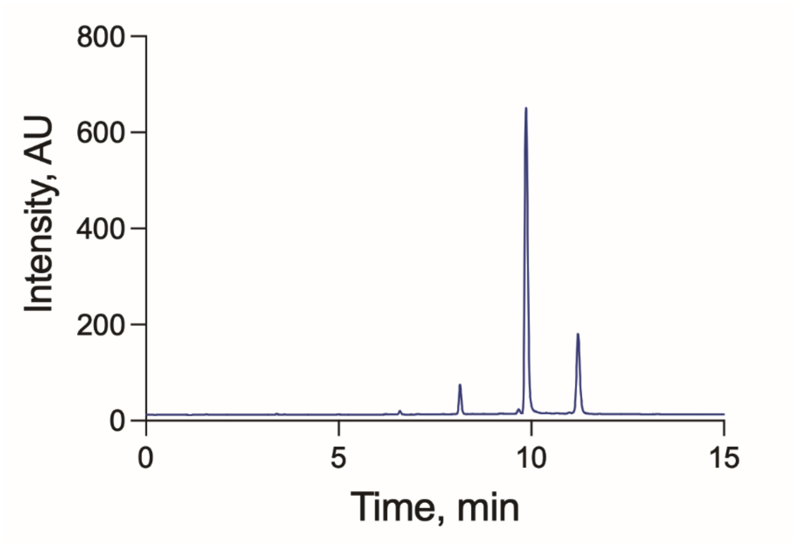

Mass spectrometry (positive ionization)

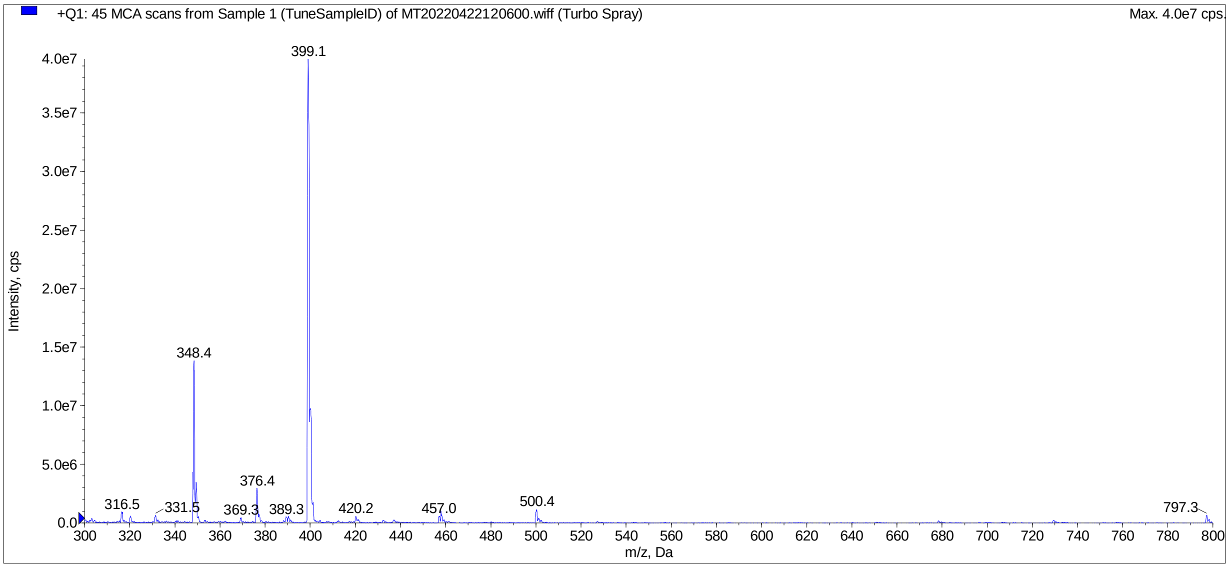

^{1}H NMR

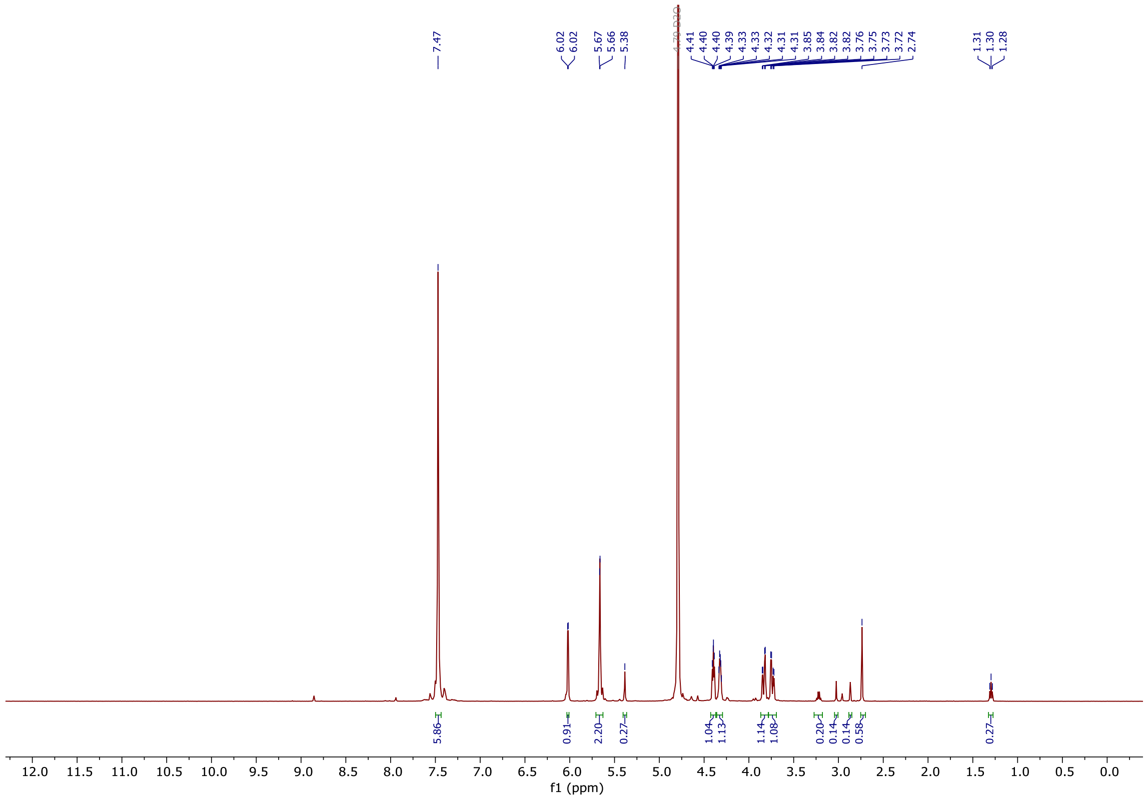

^{1}H- ^{1}H COSY NMR

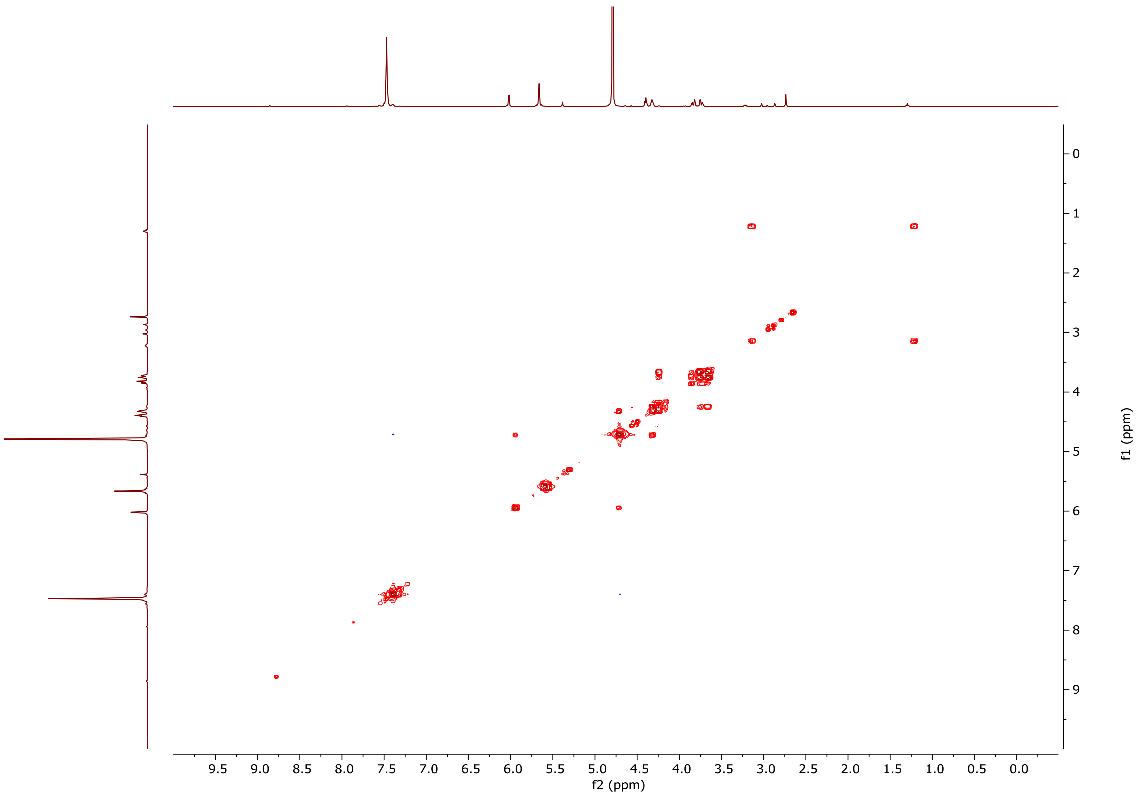

^{13}C- ^{1}H HSQC NMR

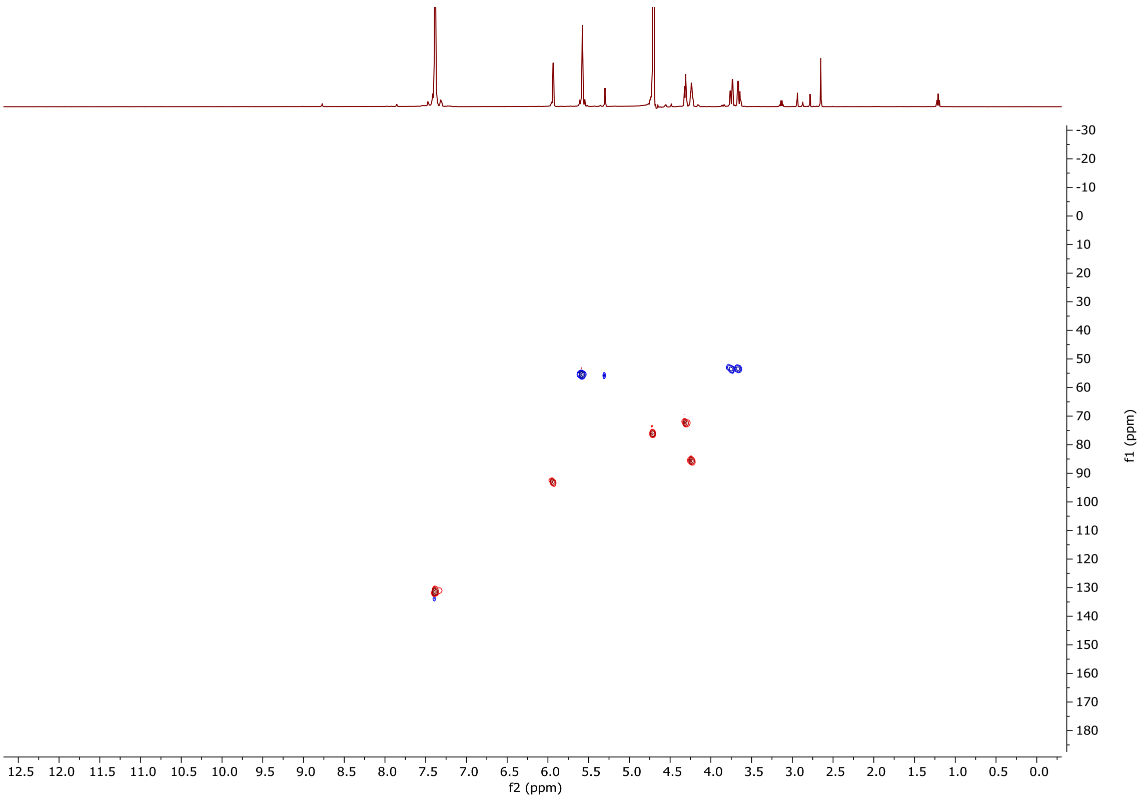

^{13}C NMR

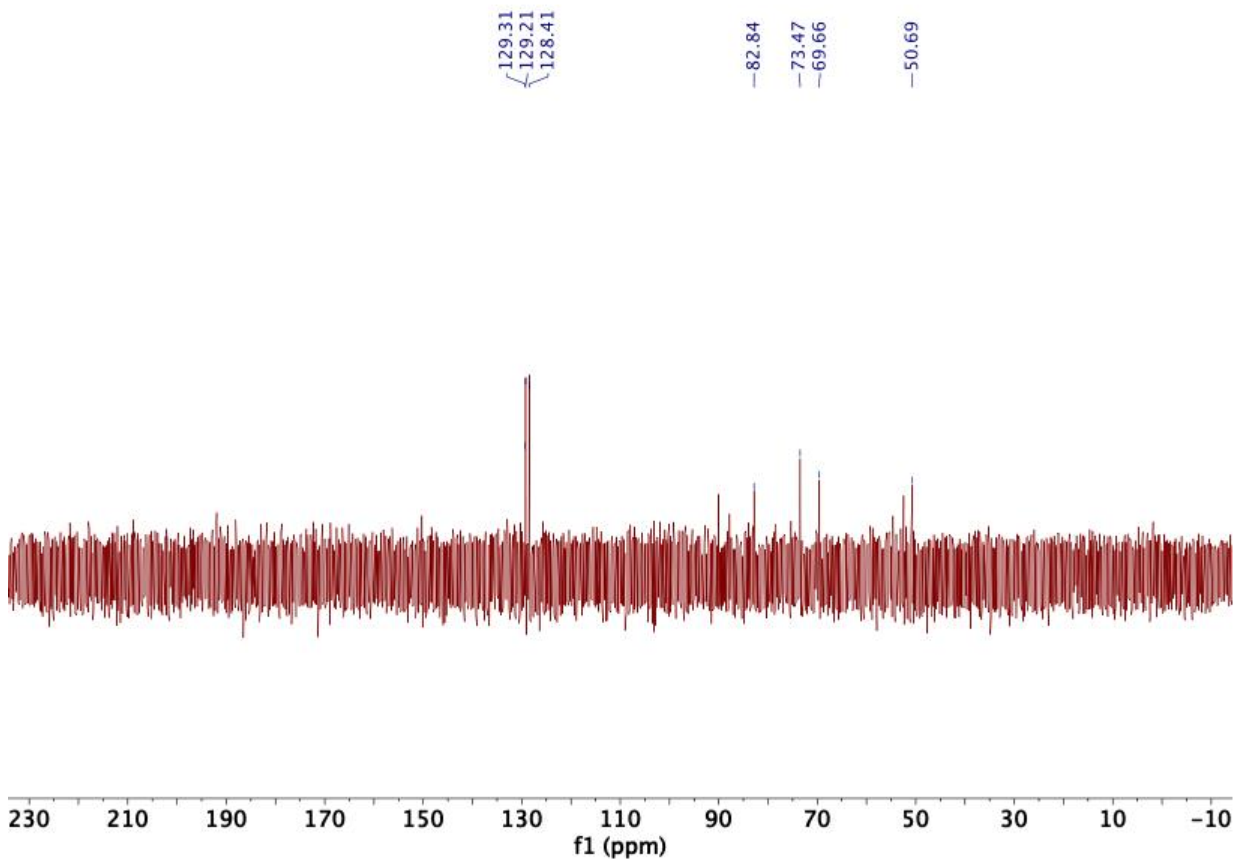

16, (5’-azido-7-(3methyl)-benzylguanosine)

RP HPLC profile (Abs, AU, 254 nm)

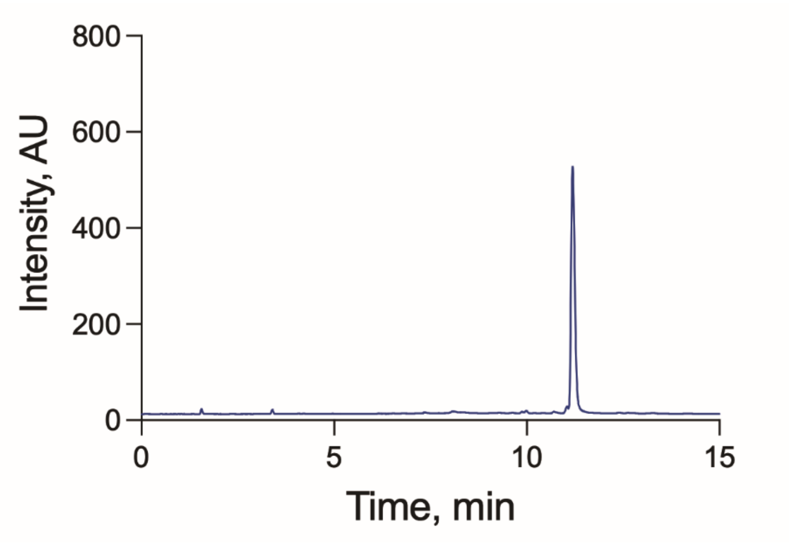

Mass spectrometry (positive ionization)

Acq. Time: 10:00
Acq. Date: Thursday, March 01, 2018
Sample Name: TuneSampleID
Acq. File: MT20180301100058.wiff
Scan Mode: Profile
Polarity/Scan Type: Positive Q1 MS

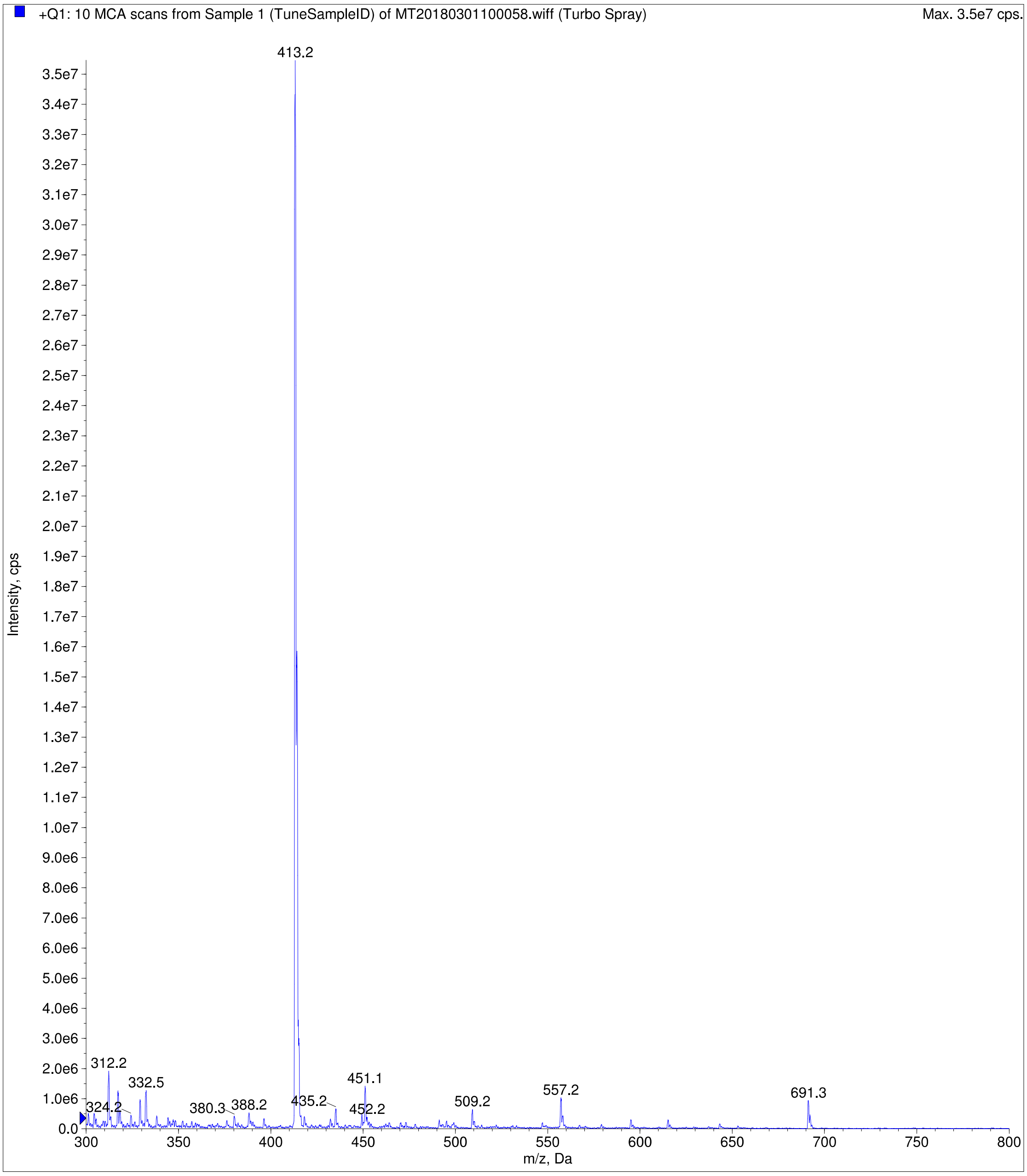

Printing Time: 10:03:27 AM
Printing Date: Thursday, March 01, 2018
Page 1 of 1
Workstation: API3200
Analyst Version: 1.5.2

^1H NMR

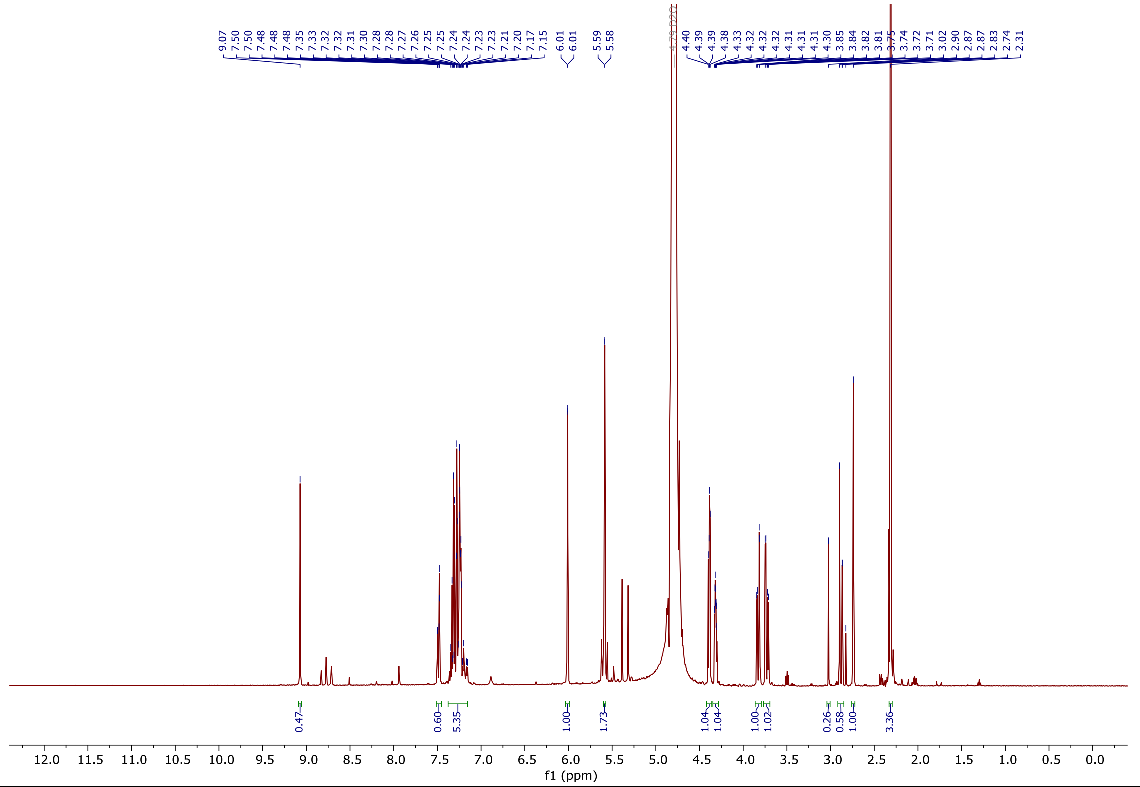

^1H- ^1H COSY NMR

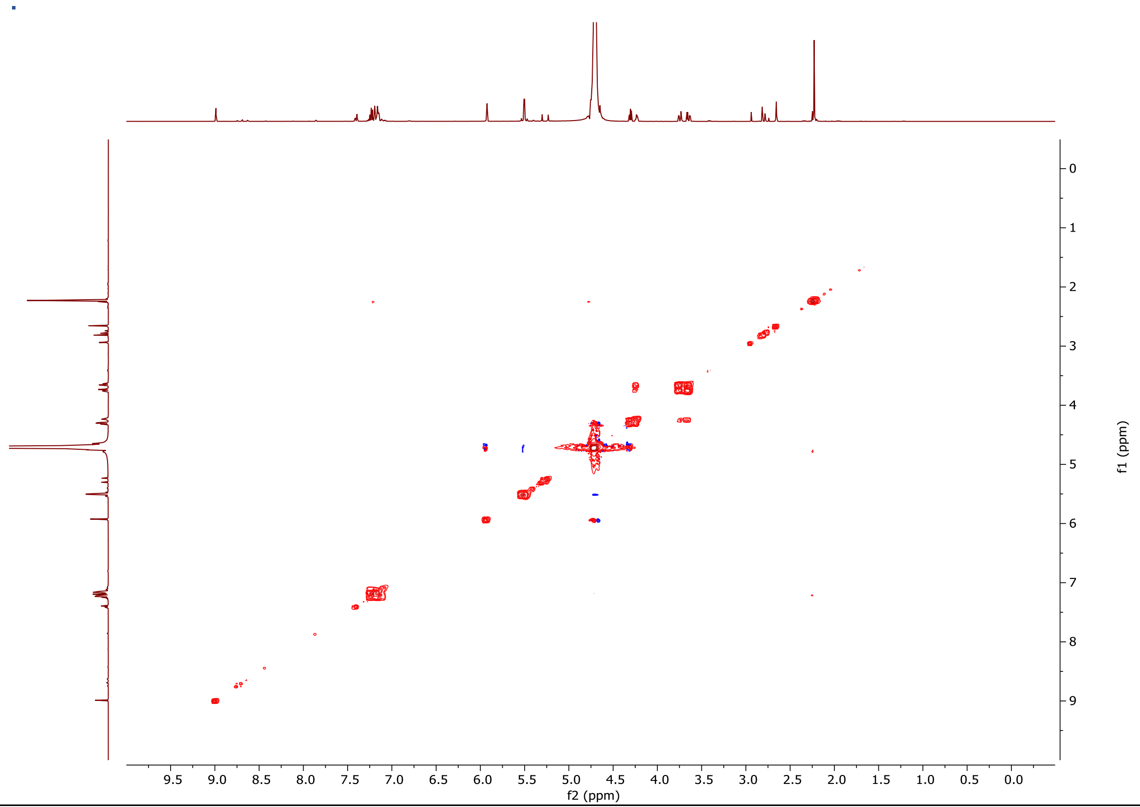

<table>
<tr><td>^{13}C- ^{1}H HSQC NMR</td><td>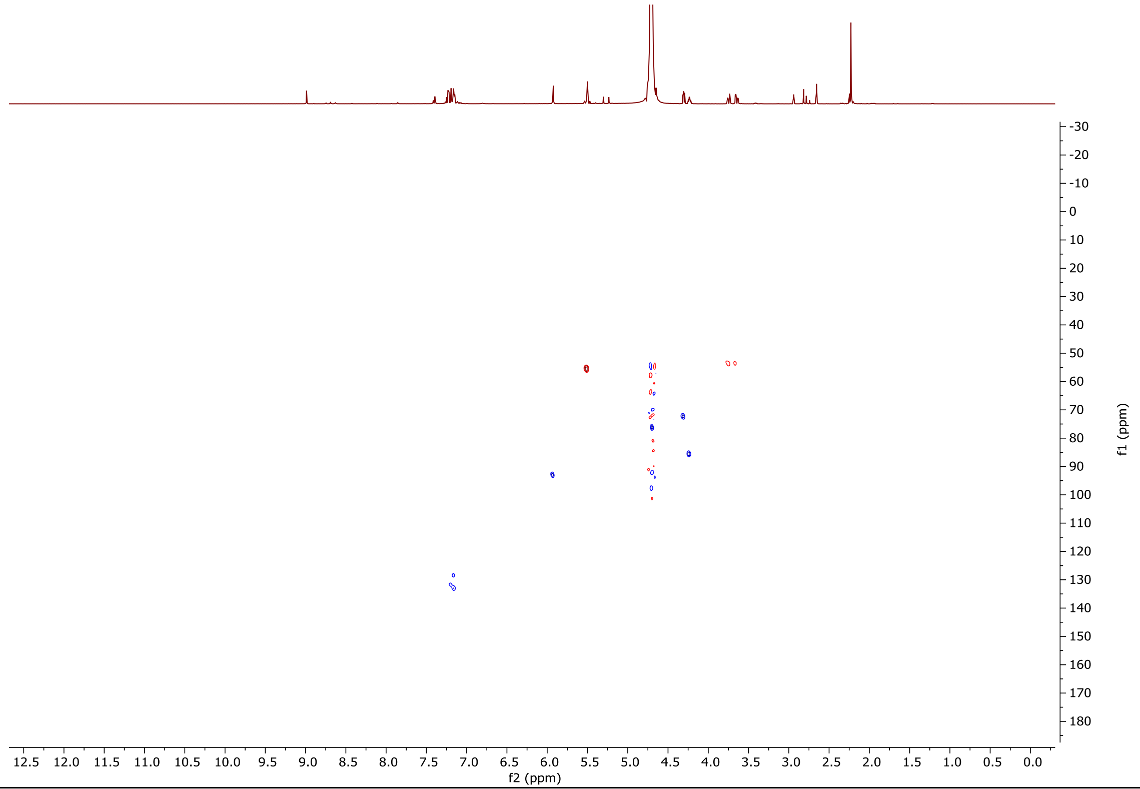</td></tr>
</table>

<table>
<tr><td colspan="2">4a, m^{7}G-triazolP (isomer 1,5)</td></tr>
<tr><td>HRMS</td><td>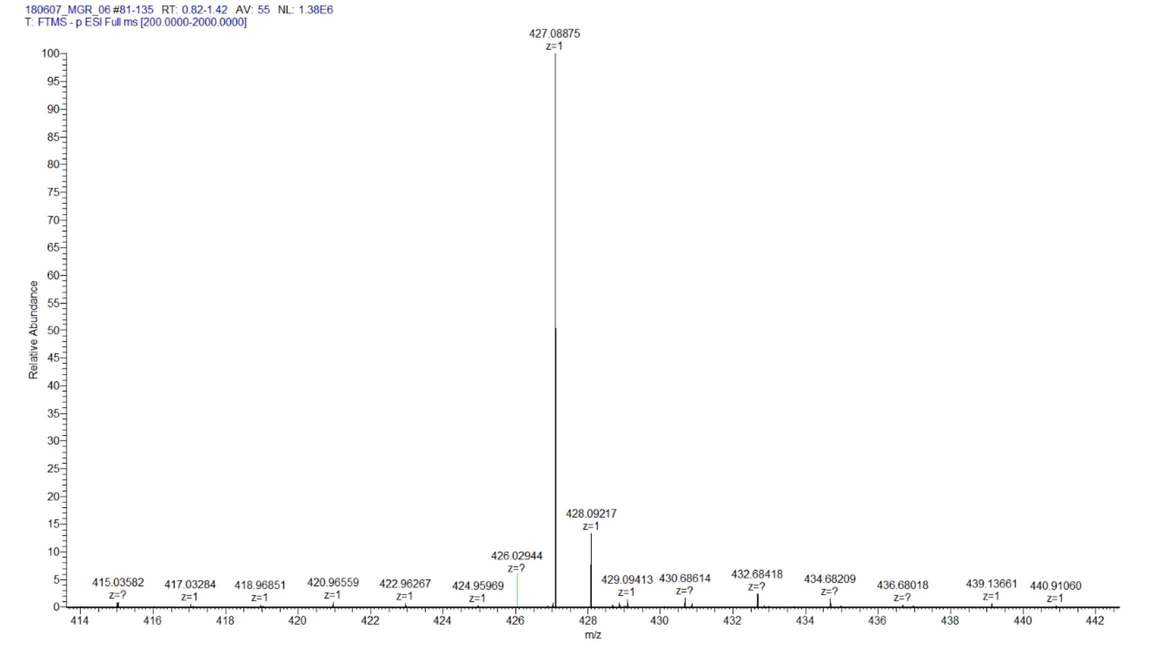</td></tr>
</table>

RP HPLC profile (Abs, AU, 254 nm)	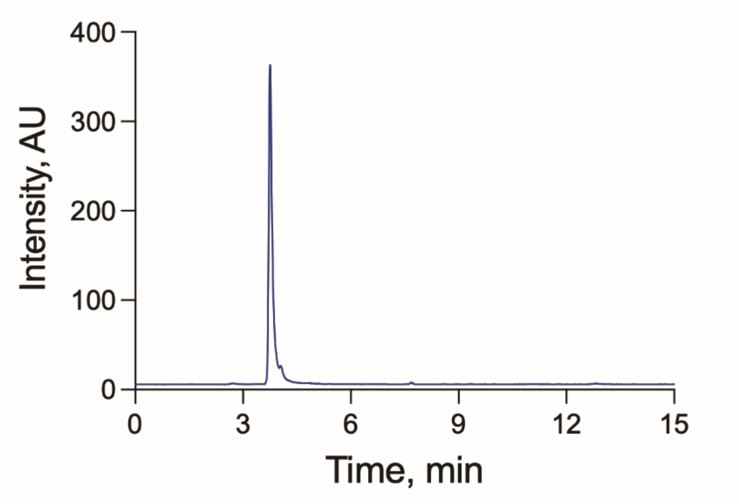
^{1}H NMR	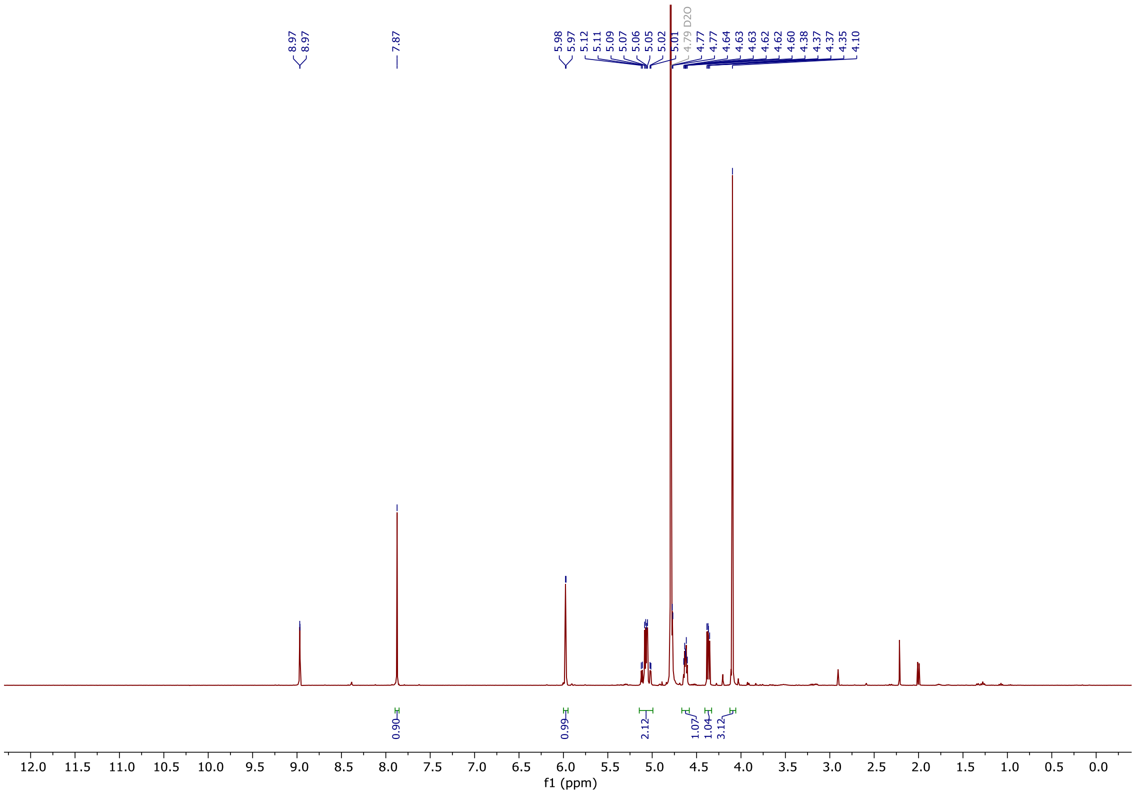

^{31}P NMR

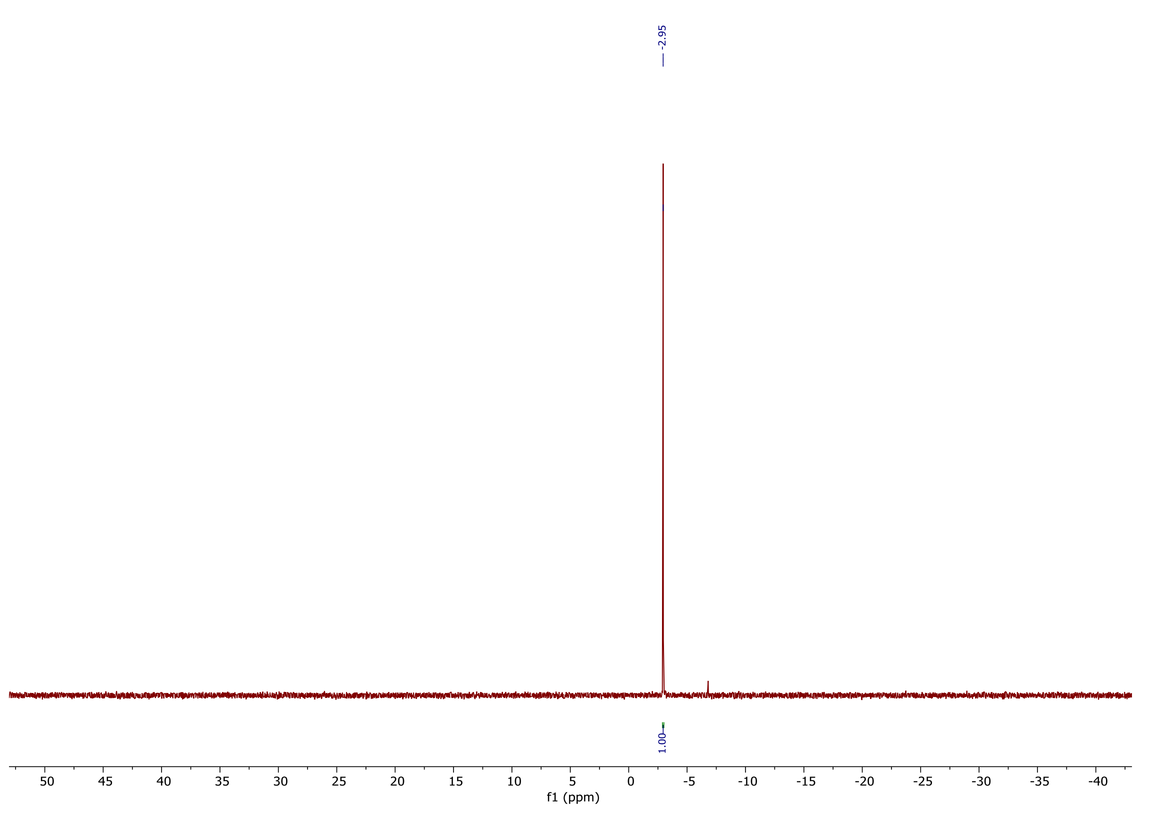

^{1}H- ^{1}H COSY NMR

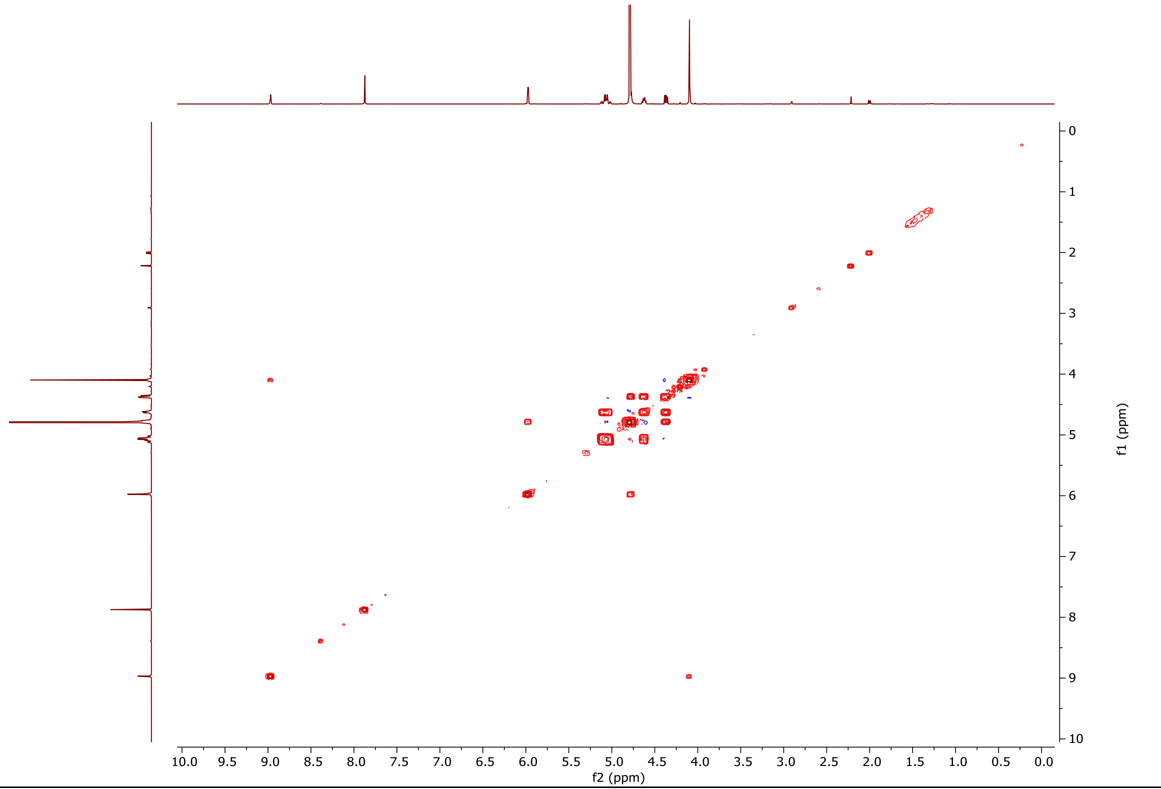

^{13}C- ^{1}H HSQC NMR

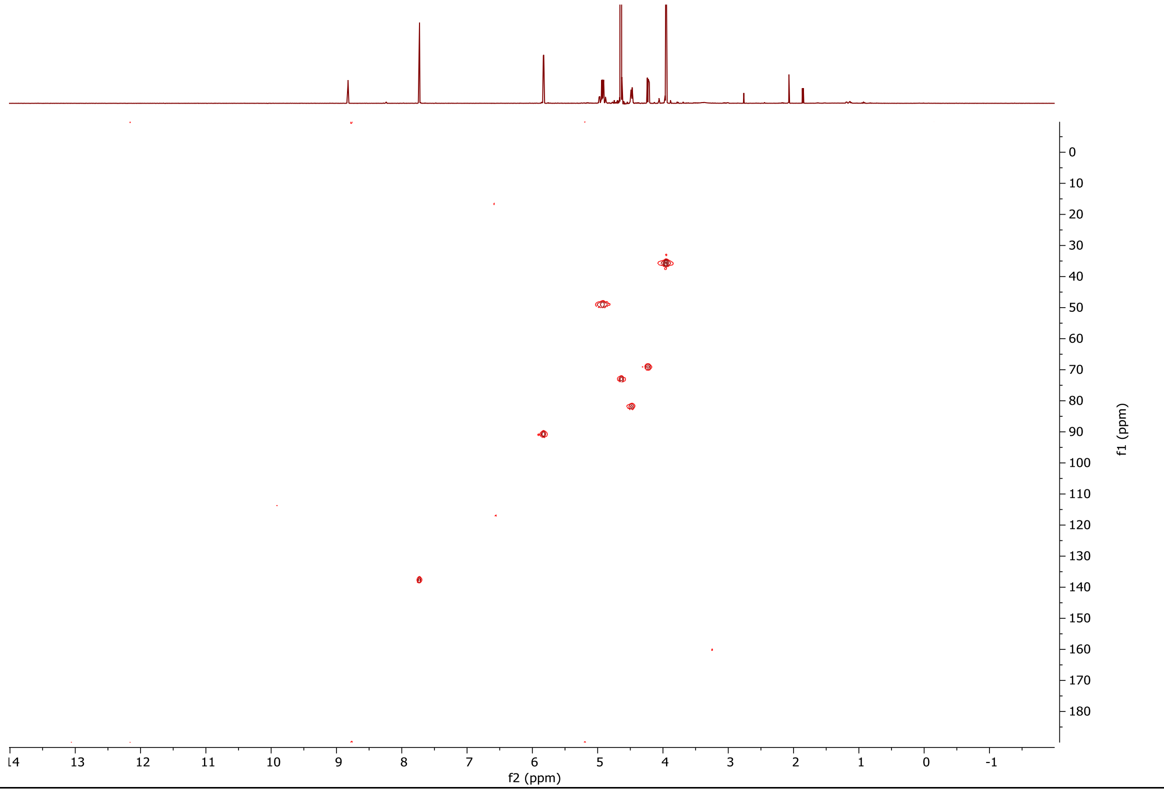

4b, m^7G-9-triazol-CH_2P

HRMS

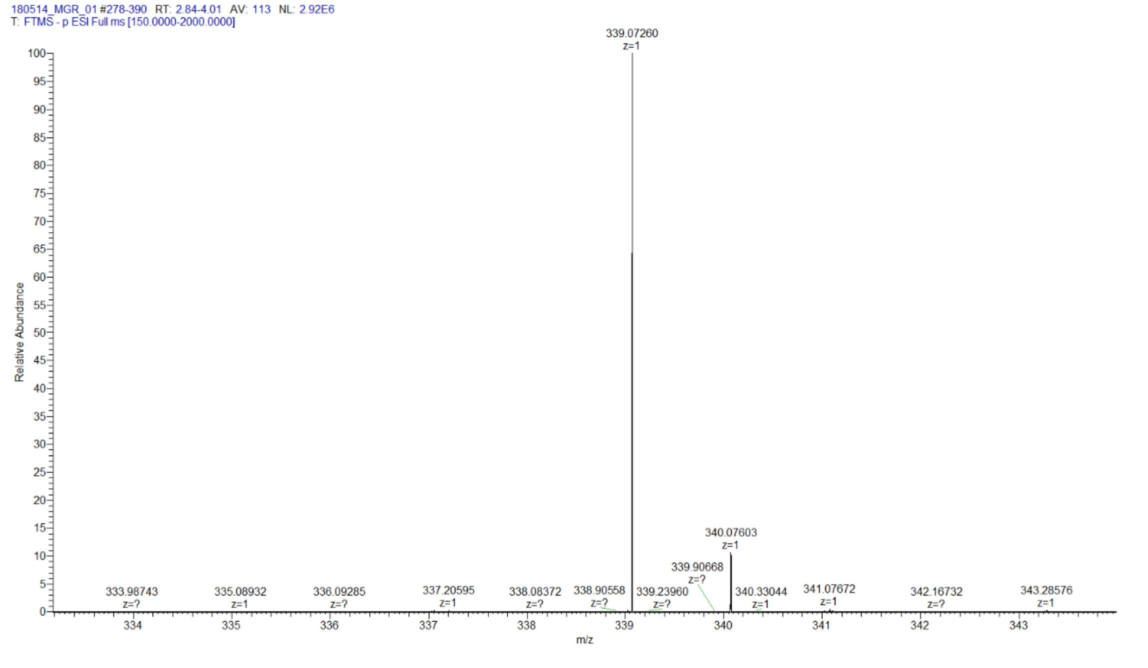

RP HPLC profile (Abs, AU, 254 nm)	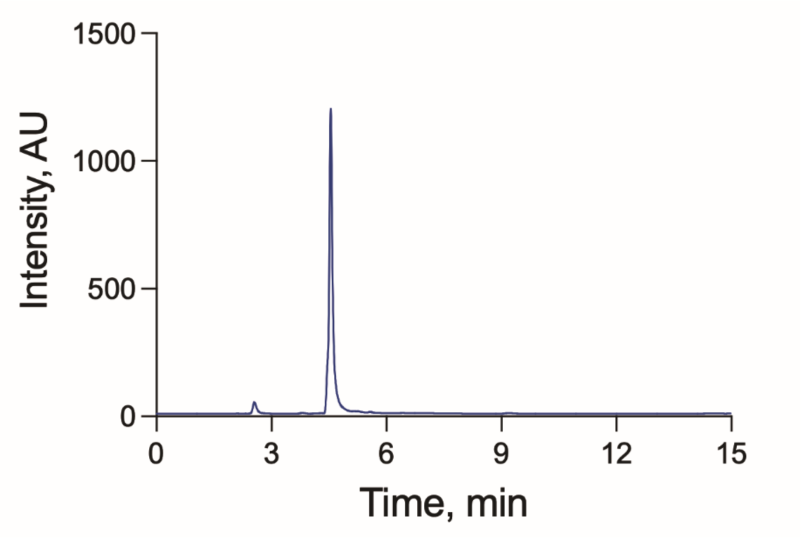
^1H NMR	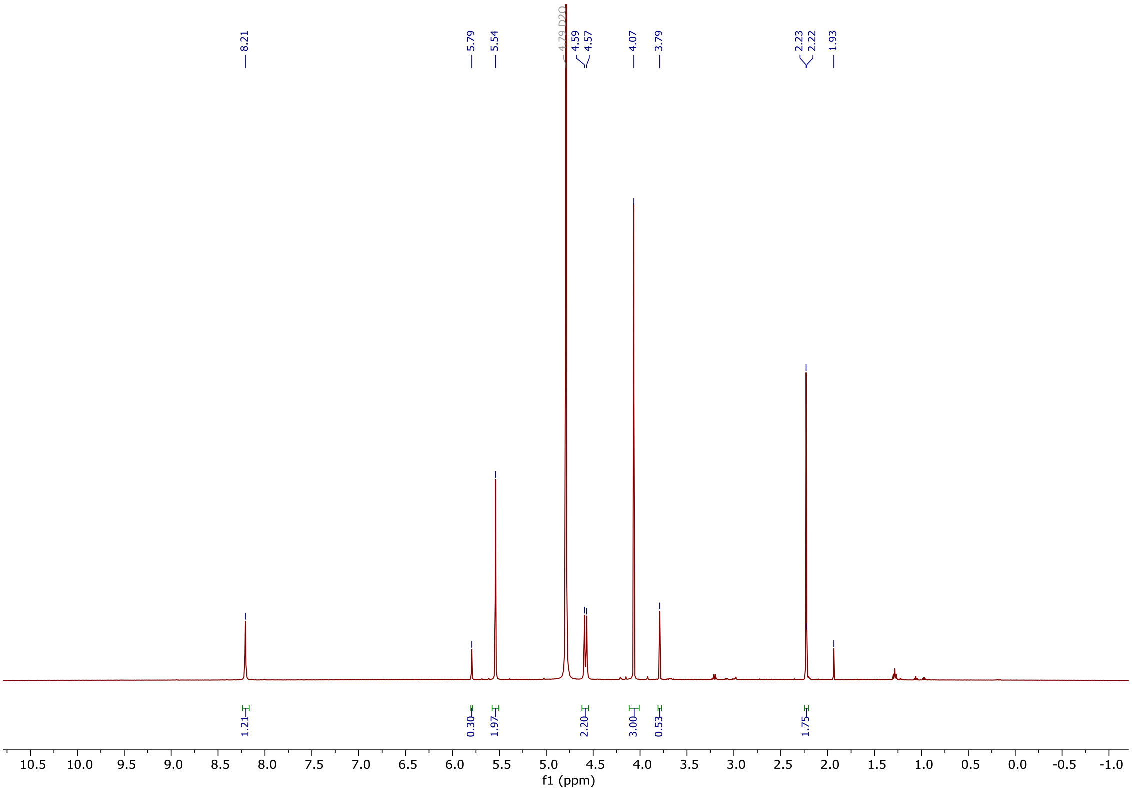

^{1}H- ^{1}H COSY NMR

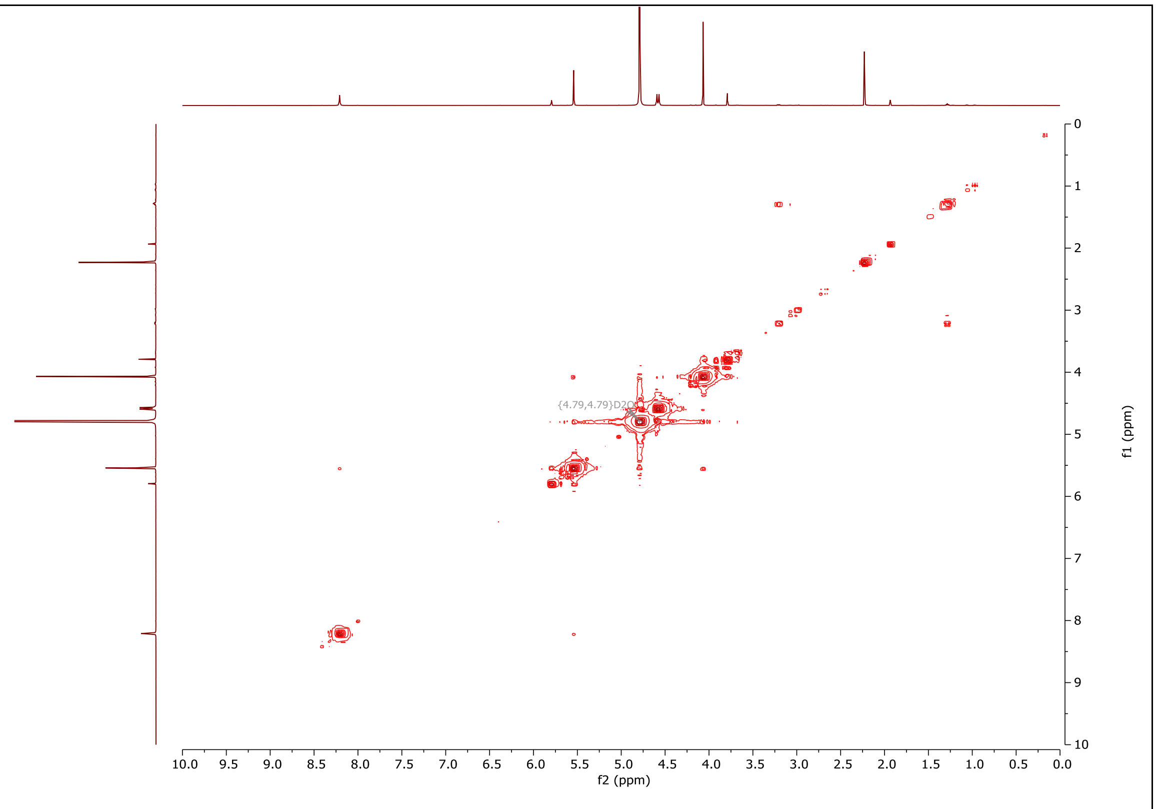

^{31}P NMR

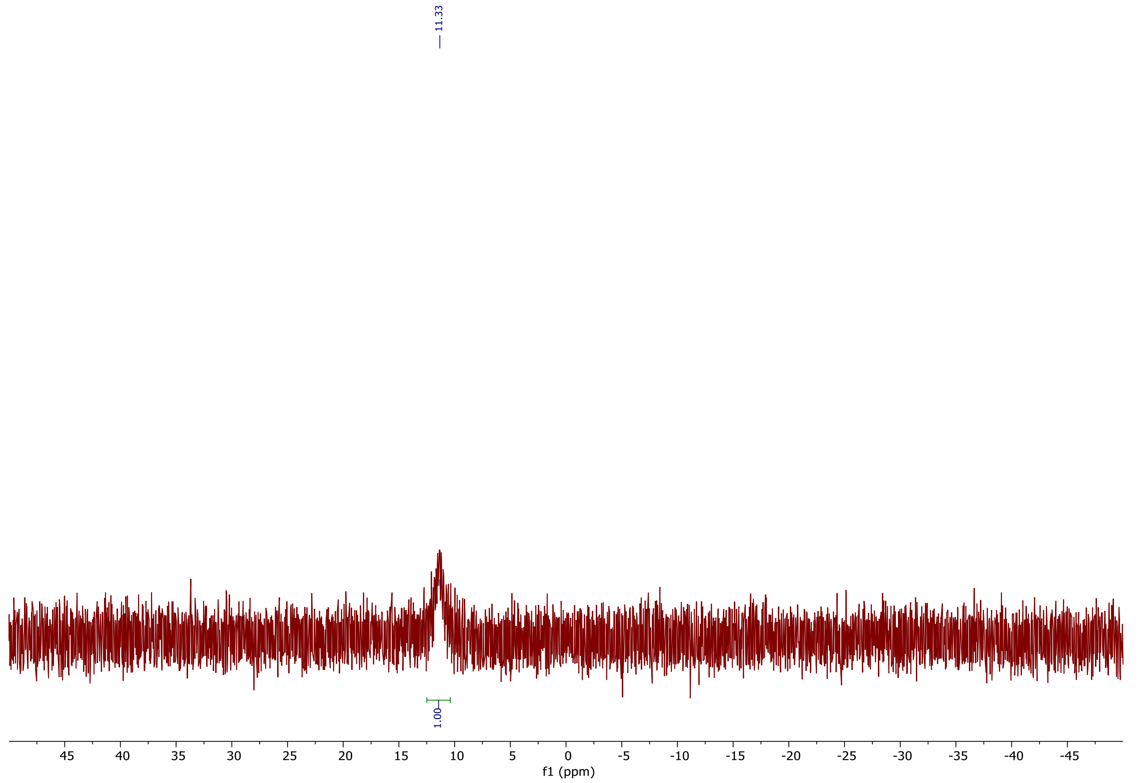

<table>
<tr><th colspan="2">**5a,** 3-MeBn^{7}GMP</th></tr>
<tr><td>HRMS</td><td>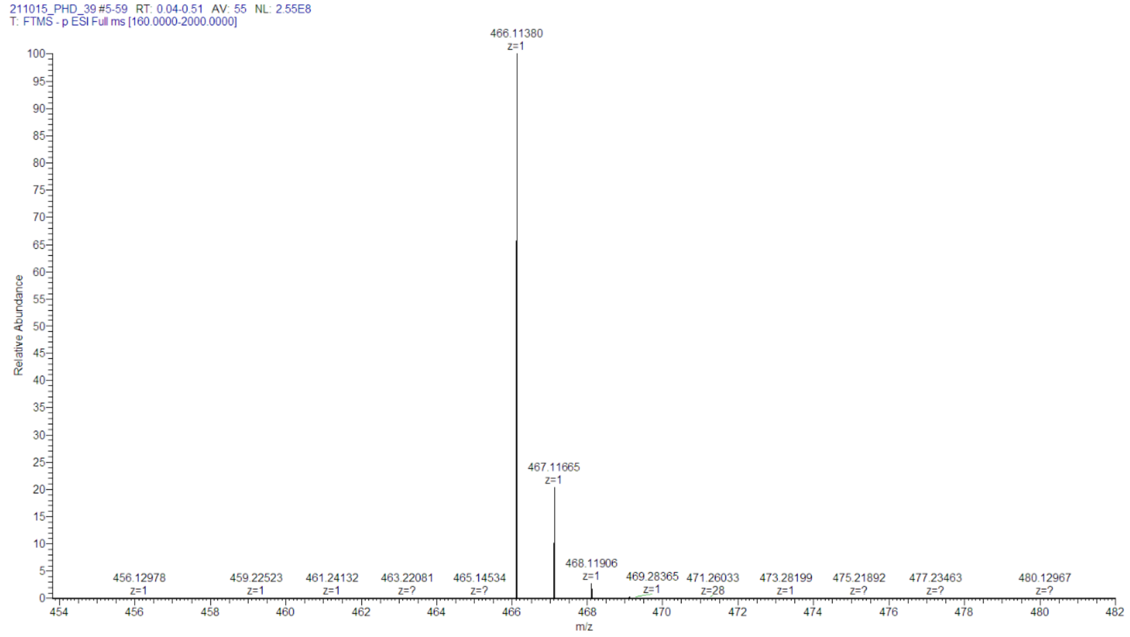
</td></tr>
<tr><td>RP HPLC profile (Abs, AU, 254 nm)</td><td>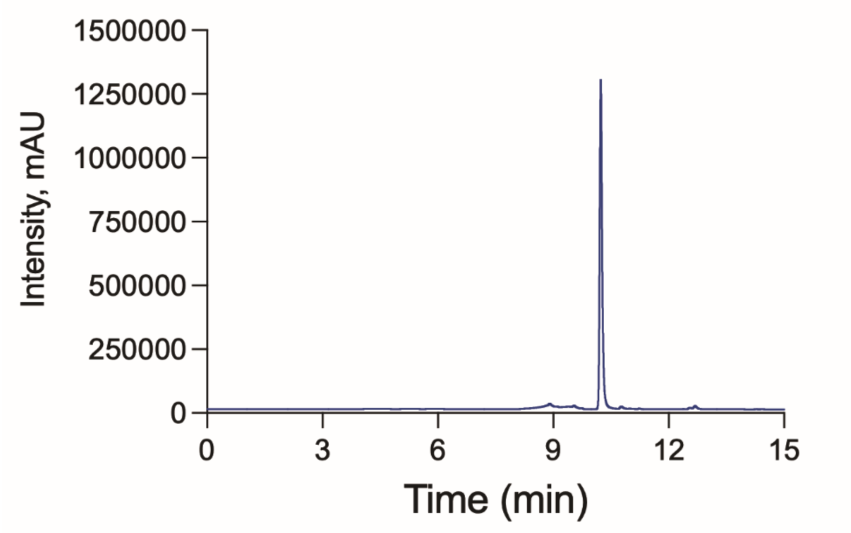
</td></tr>
</table>

**5b,** 4-MeBn^{7}GMP	
HRMS	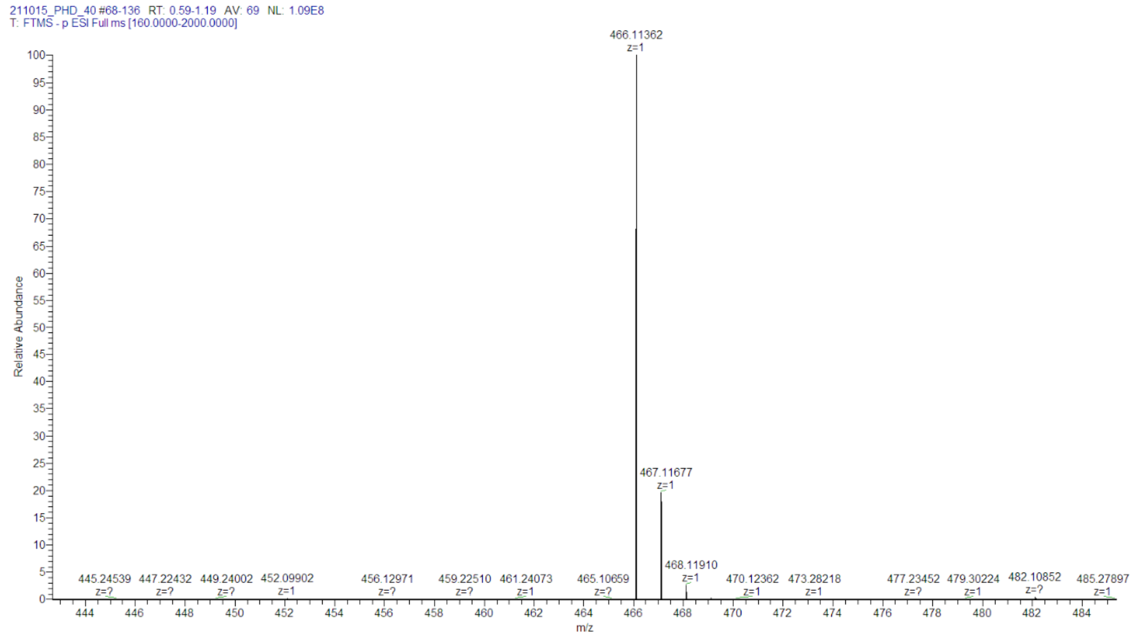
RP HPLC profile (Abs, AU, 254 nm)	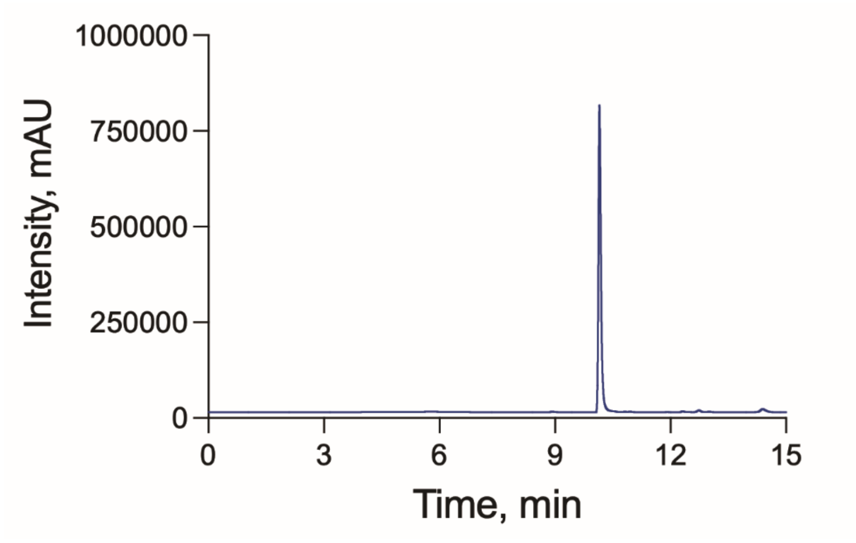

<table>
<tr><th colspan="2">5c, 3,5-Me_2Bn^7GMP</th></tr>
<tr><td>HRMS</td><td>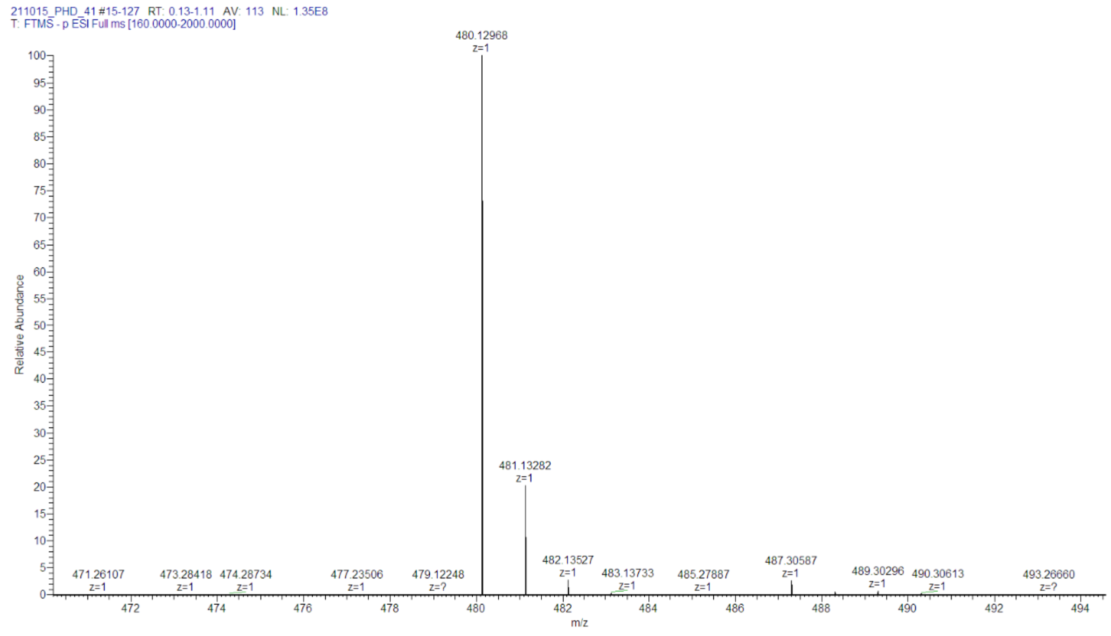
</td></tr>
<tr><td>RP HPLC profile (Abs, AU, 254 nm)</td><td>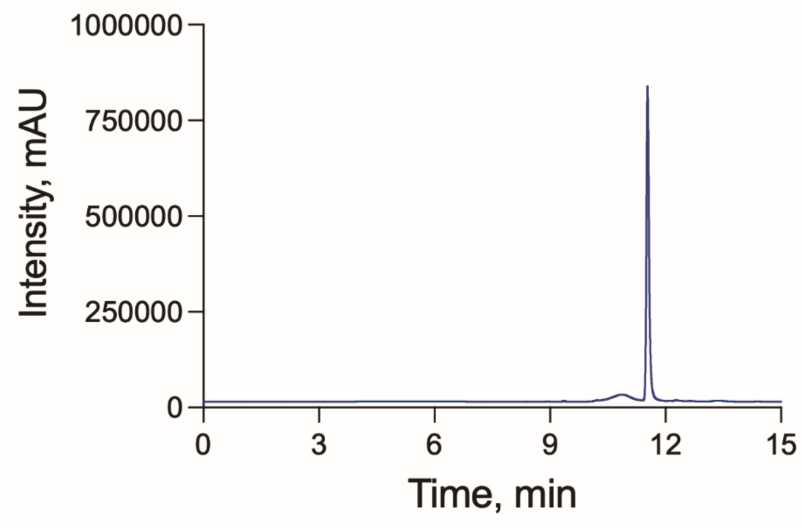
</td></tr>
</table>

5d, 3,4-F_2-Bn^7GMP

HRMS

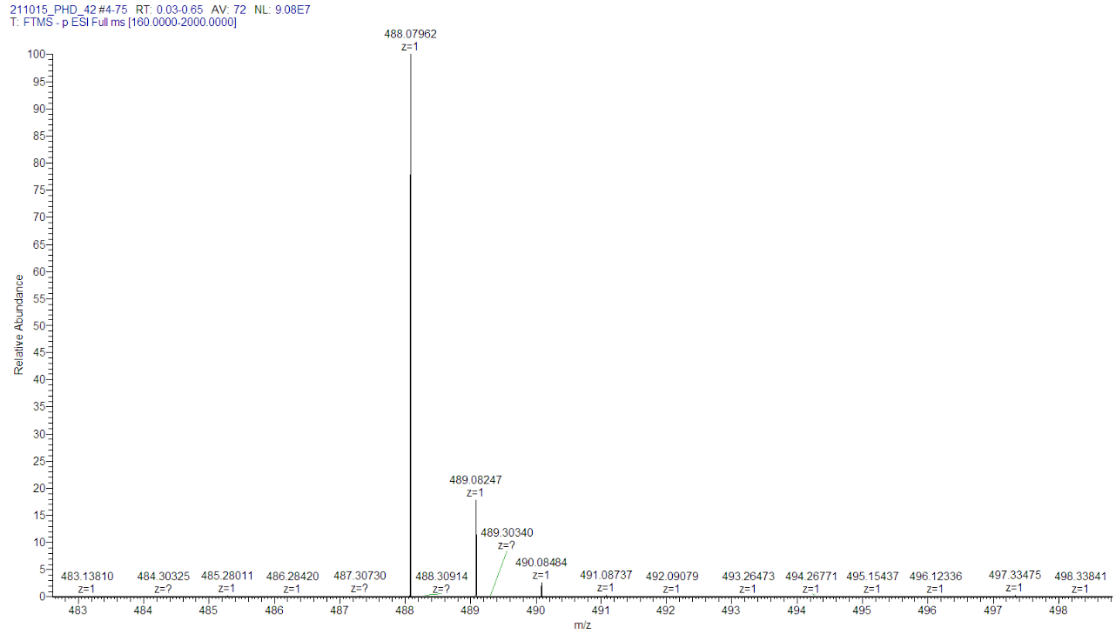

RP HPLC profile (Abs, AU, 254 nm)

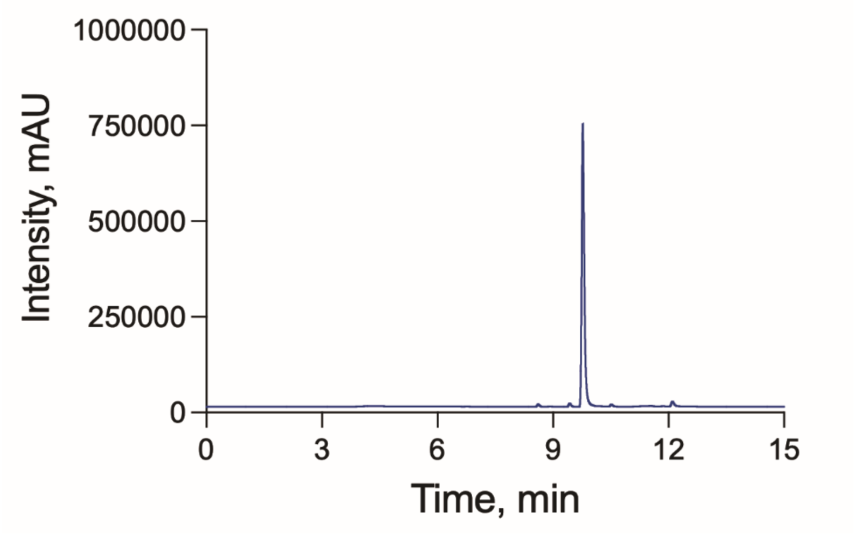

**5e,** 2,4-F_2-Bn^7GMP	
HRMS	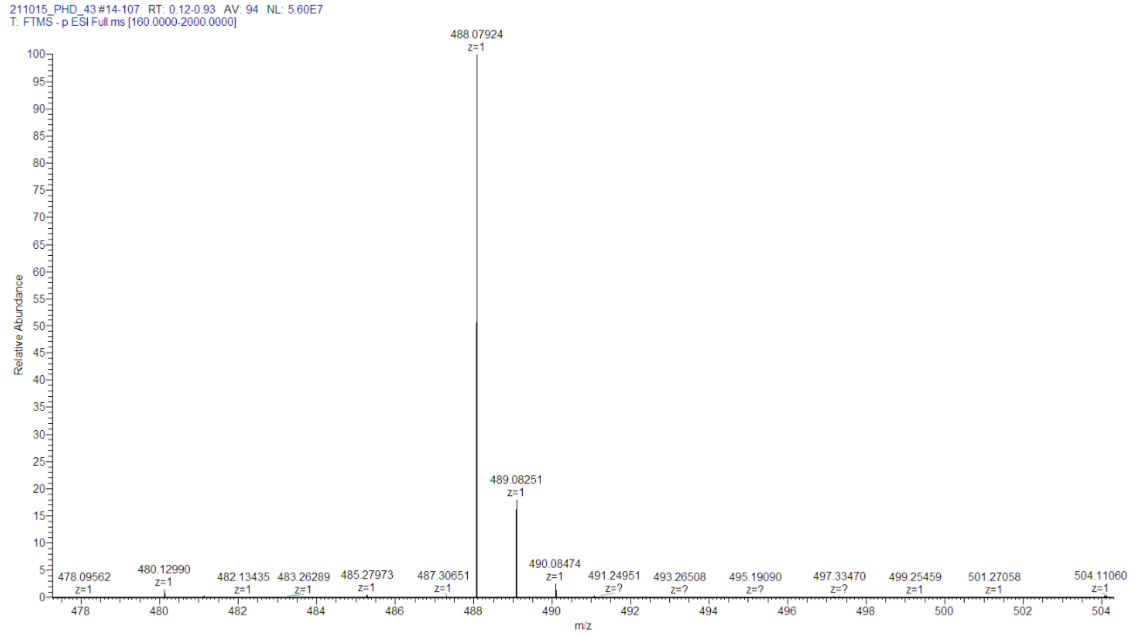
RP HPLC profile (Abs, AU, 254 nm)	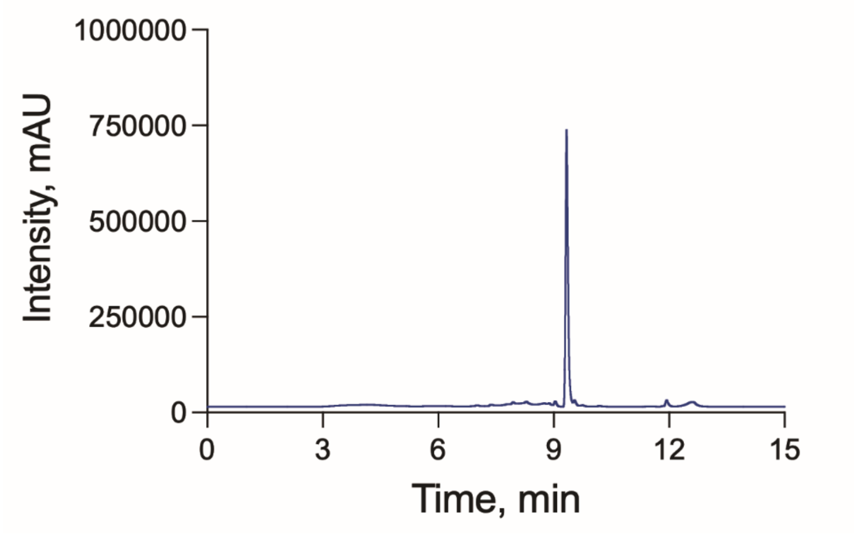

**5f,** 3,4,5-F_3-Bn^7GMP	
HRMS	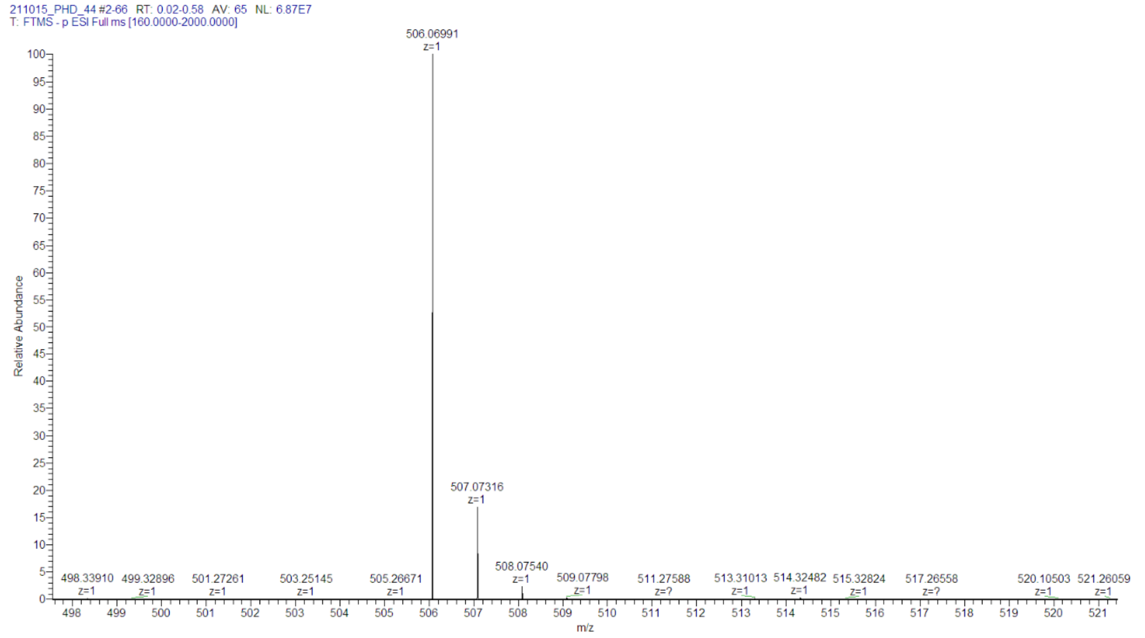

**5g,** 4-CF_3-Bn^7GMP	
HRMS	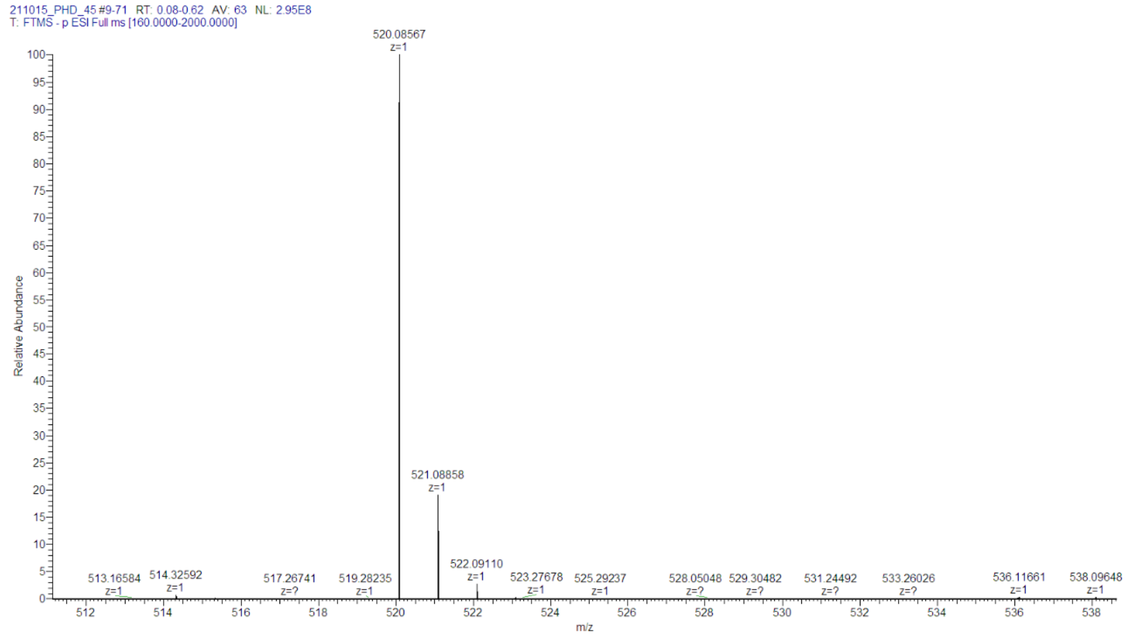

RP HPLC profile (Abs, AU, 254 nm)	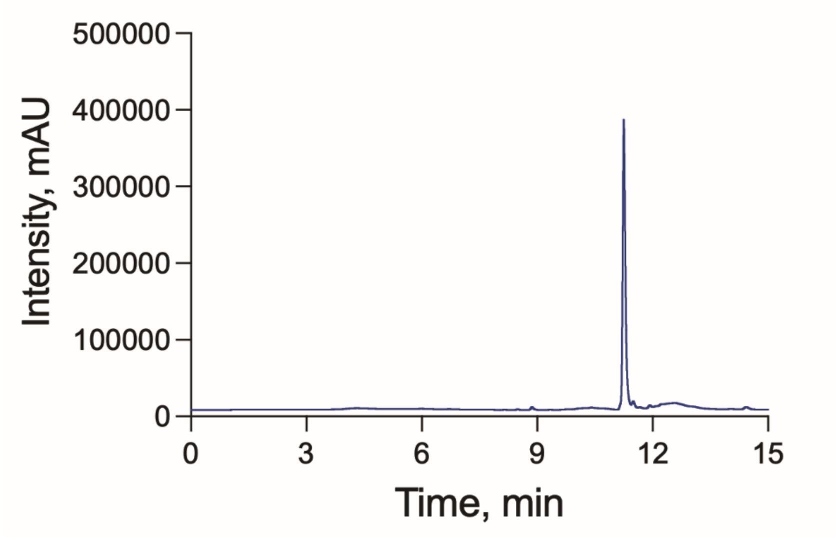

**8a,** Bn^{7}GMPF	
HRMS	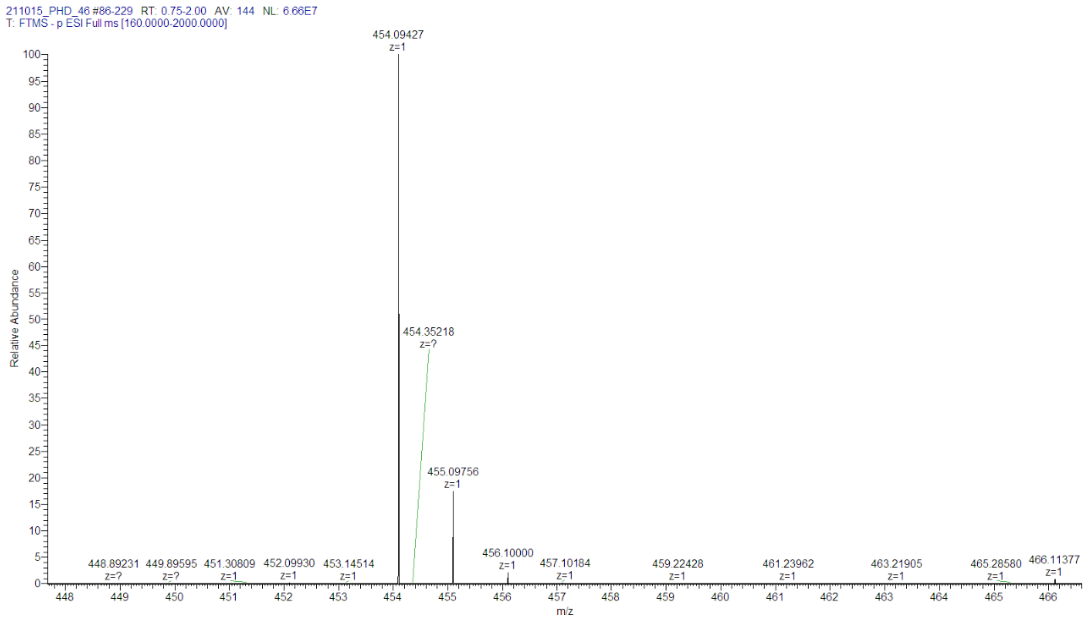
RP HPLC profile (Abs, AU, 254 nm)	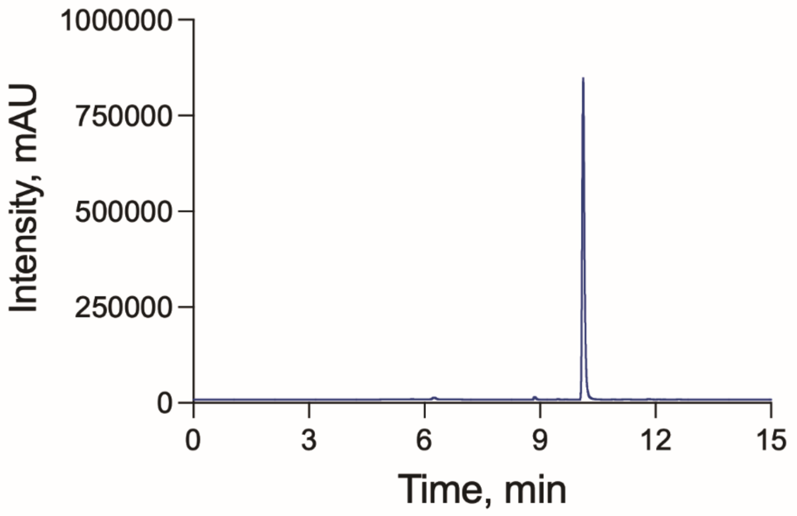

**8b,** Bn[7]GDPF	
HRMS	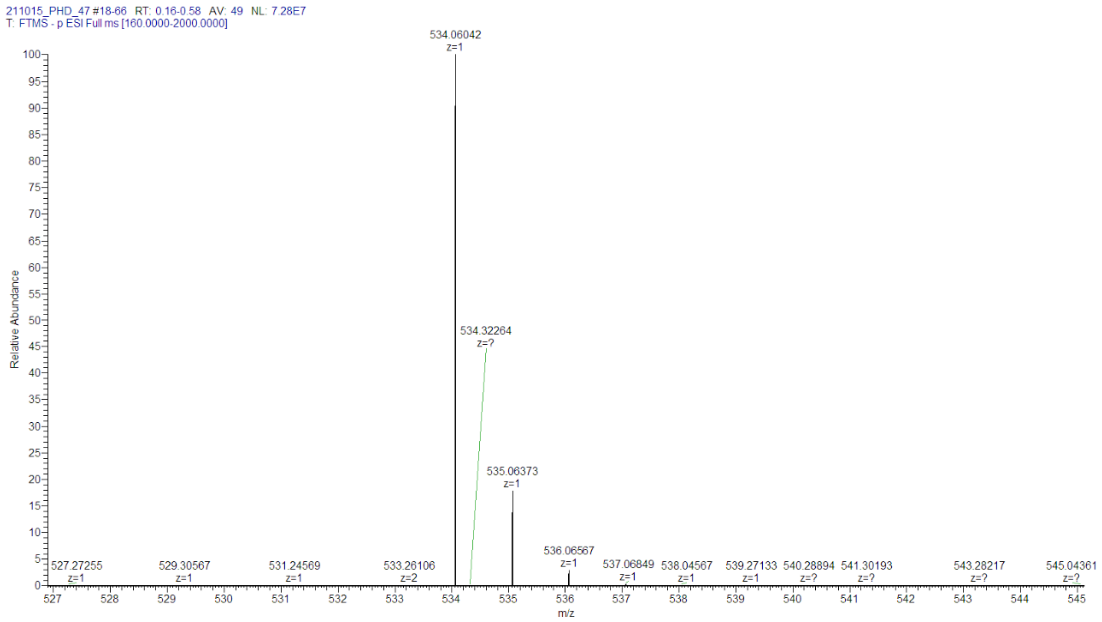
RP HPLC profile (Abs, AU, 254 nm)	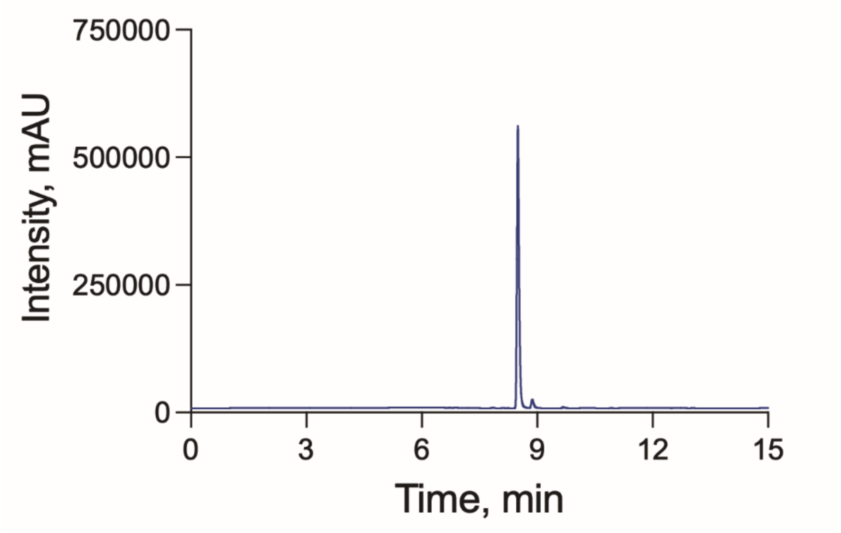

8c, Bn^{7}GMPH

HRMS

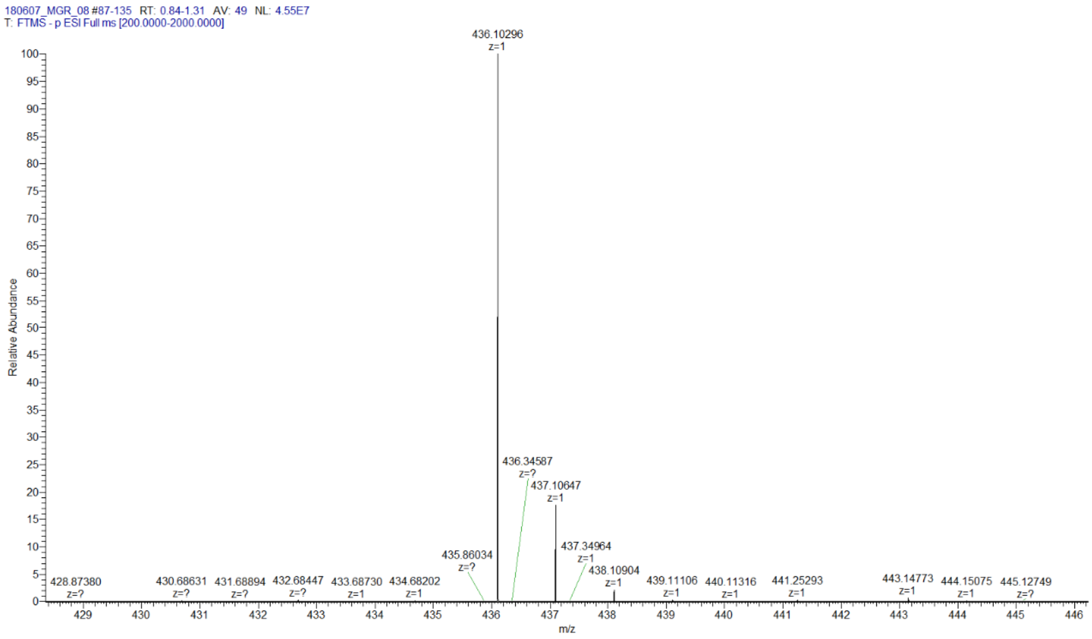

RP HPLC profile (Abs, AU, 254 nm)

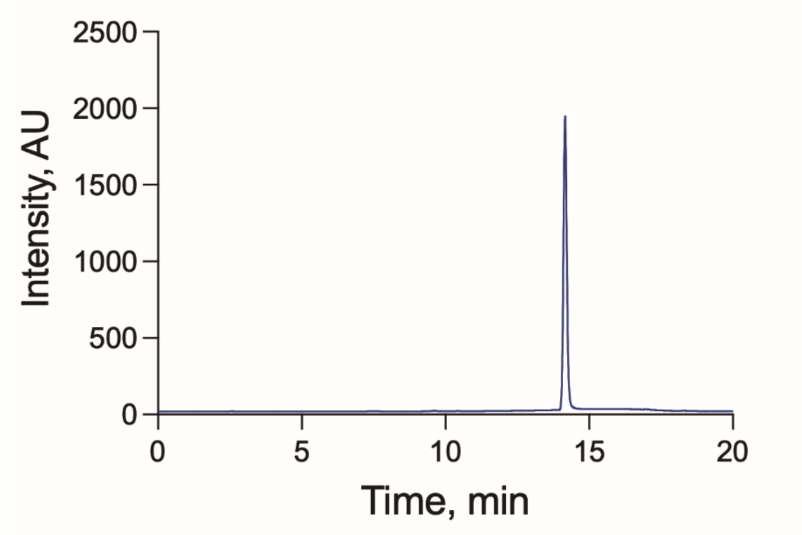

^1H NMR

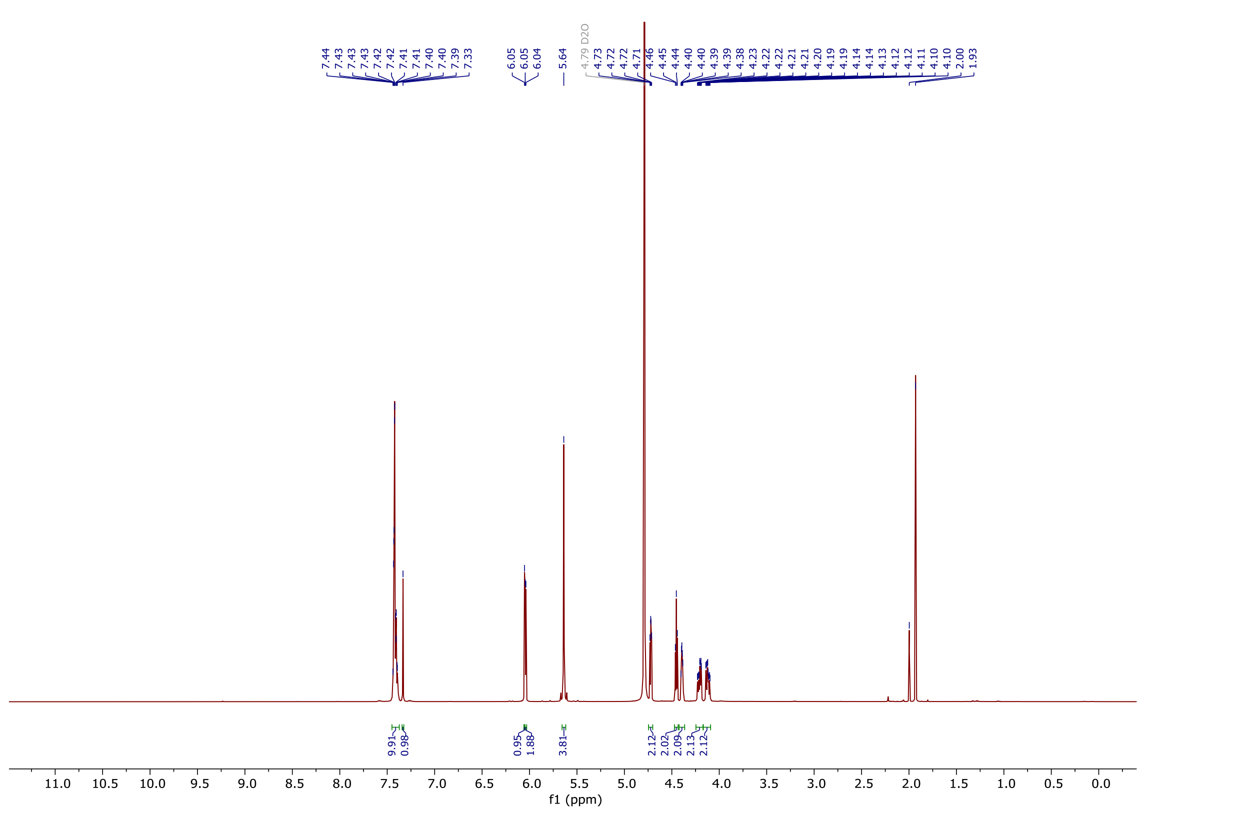

^1H- ^1H COSY NMR

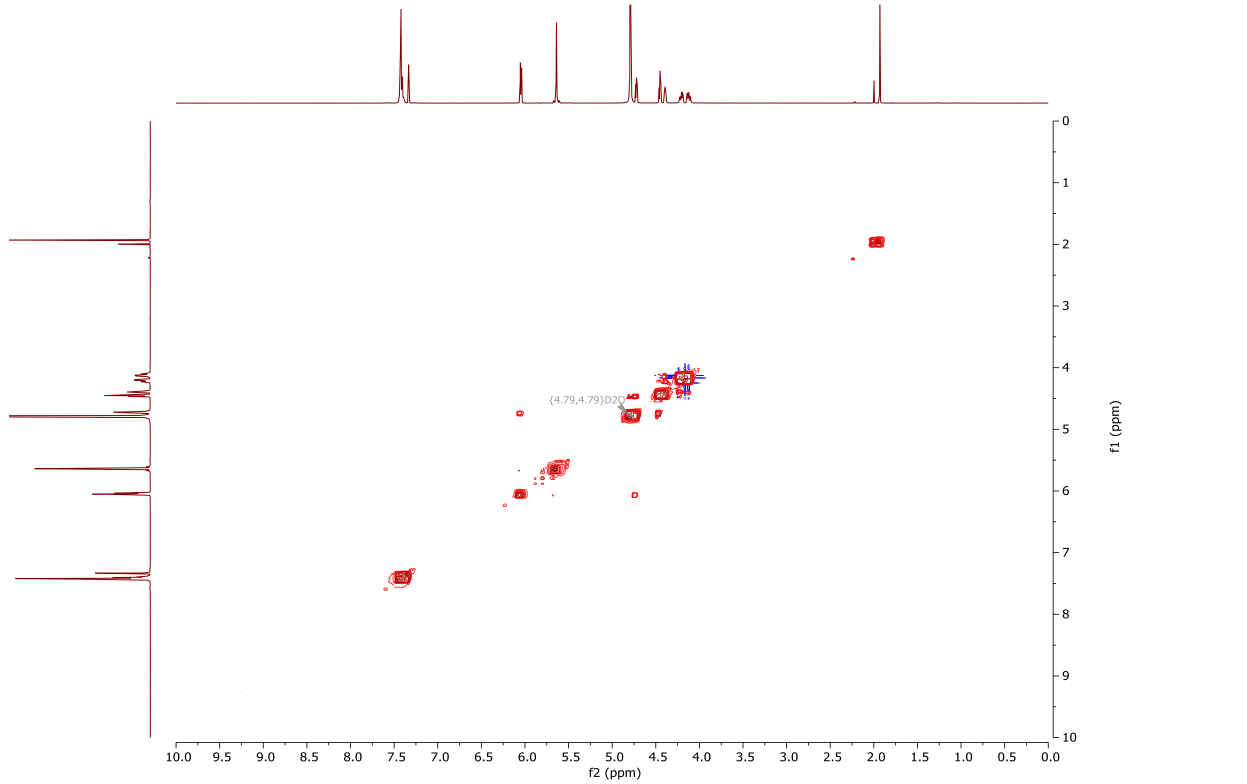

^{13}C- ^{1}H HSQC NMR	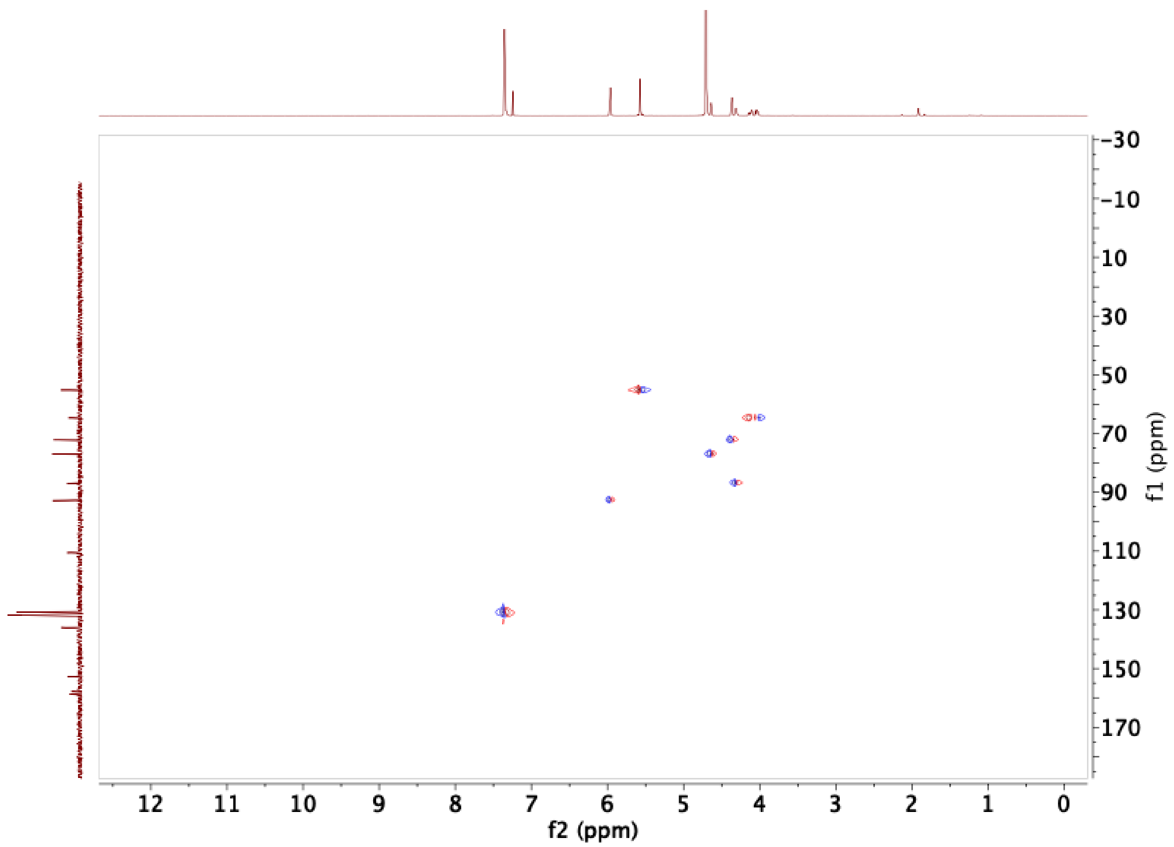
^{31}P NMR	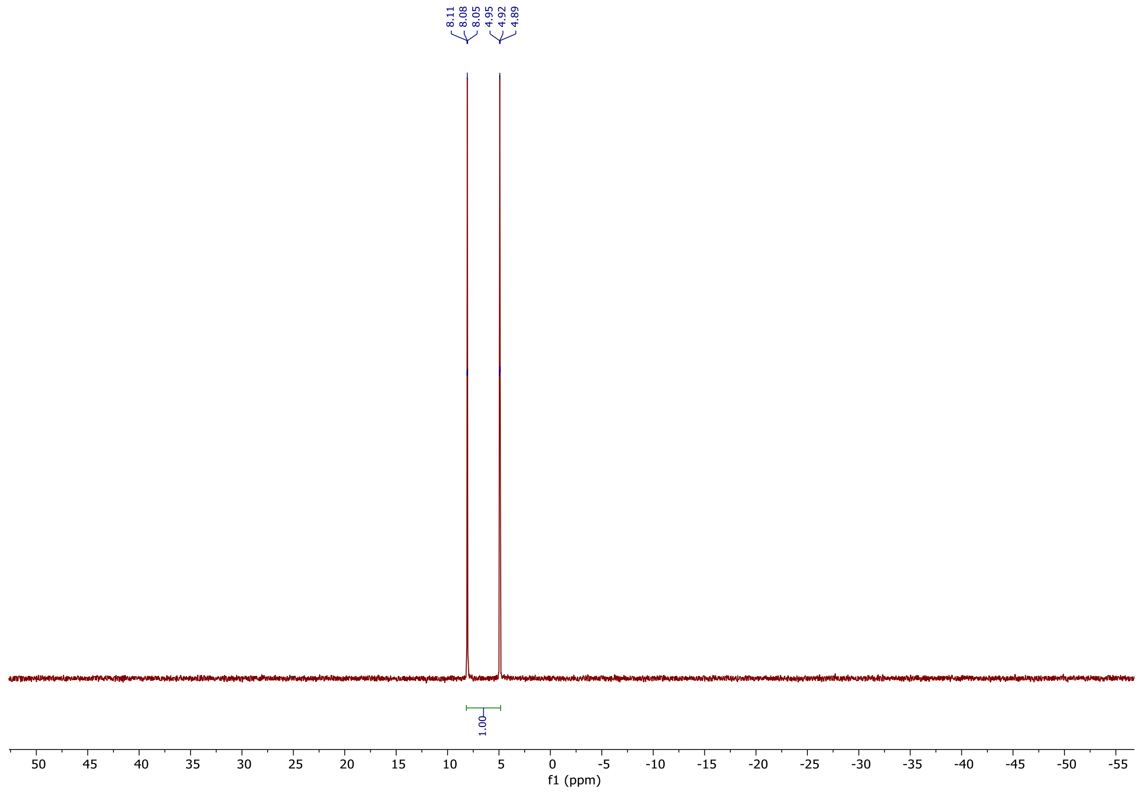

^{13}C NMR

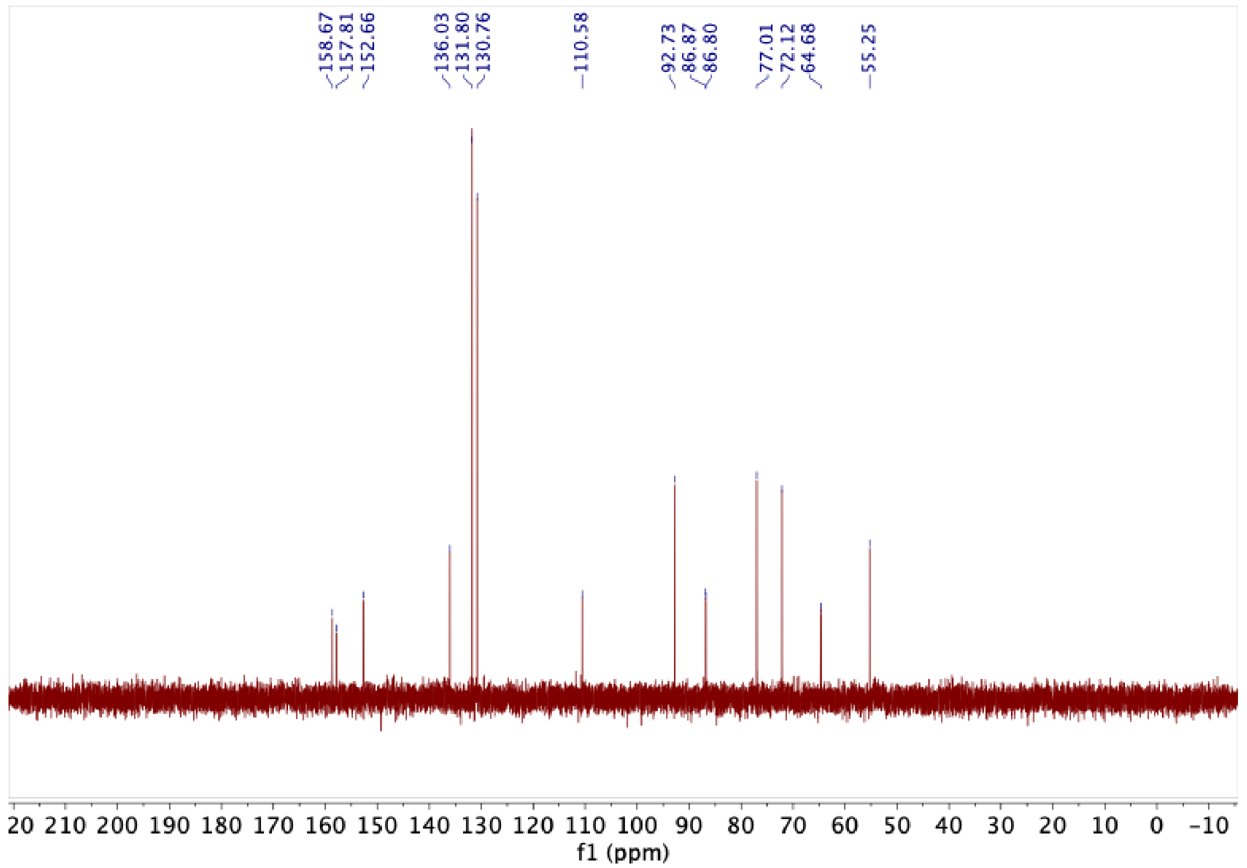

8d, Bn^{7}G-triazolP

HRMS

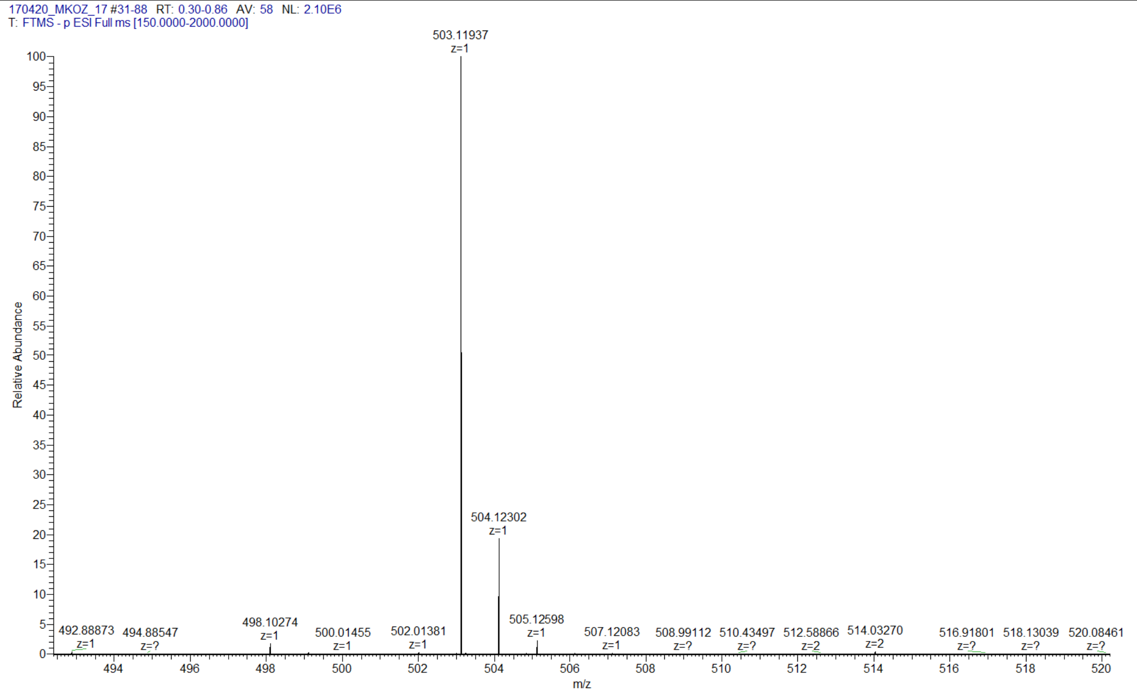

RP HPLC profile (Abs, AU, 254 nm)	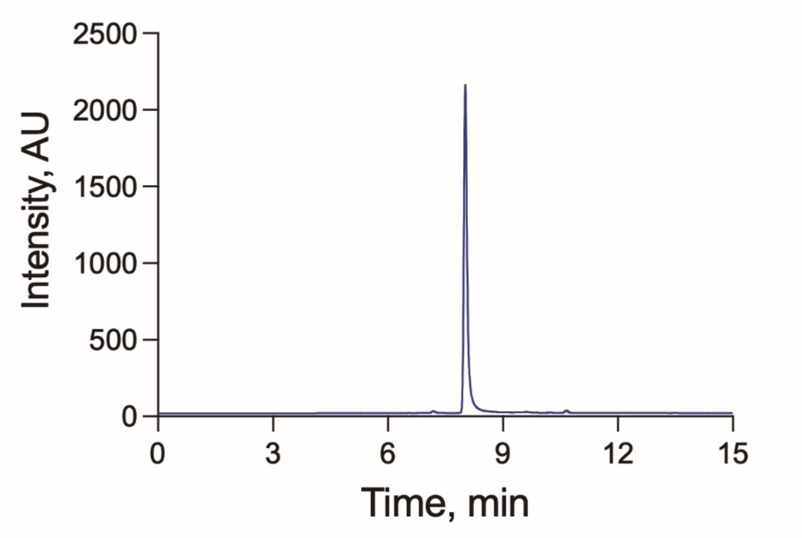
^{1}H NMR	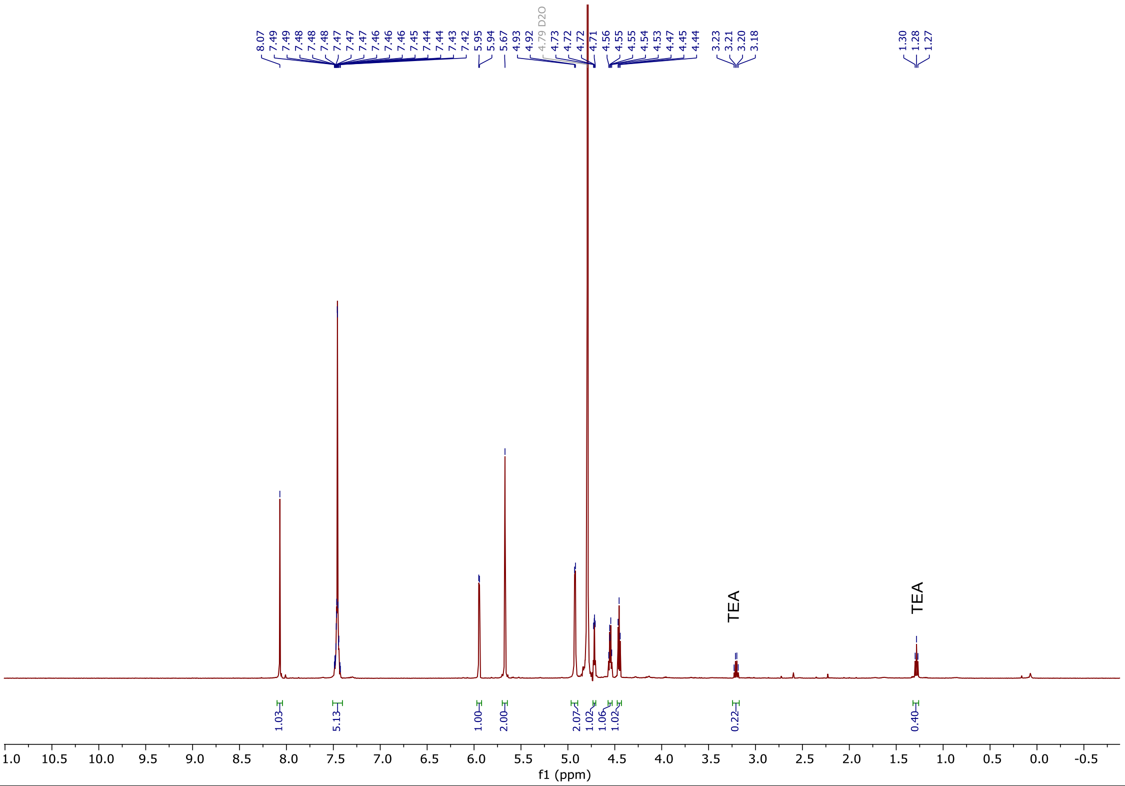

^{31}P NMR	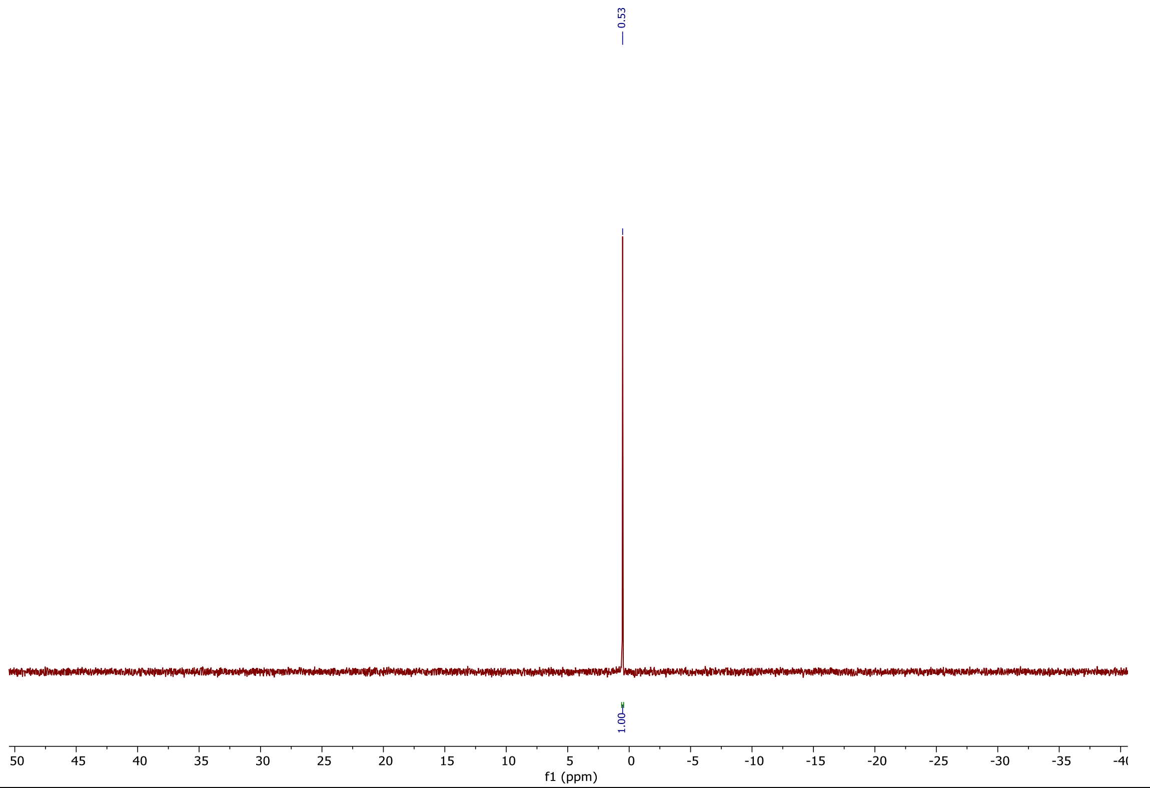
^{1}H- ^{1}H COSY NMR	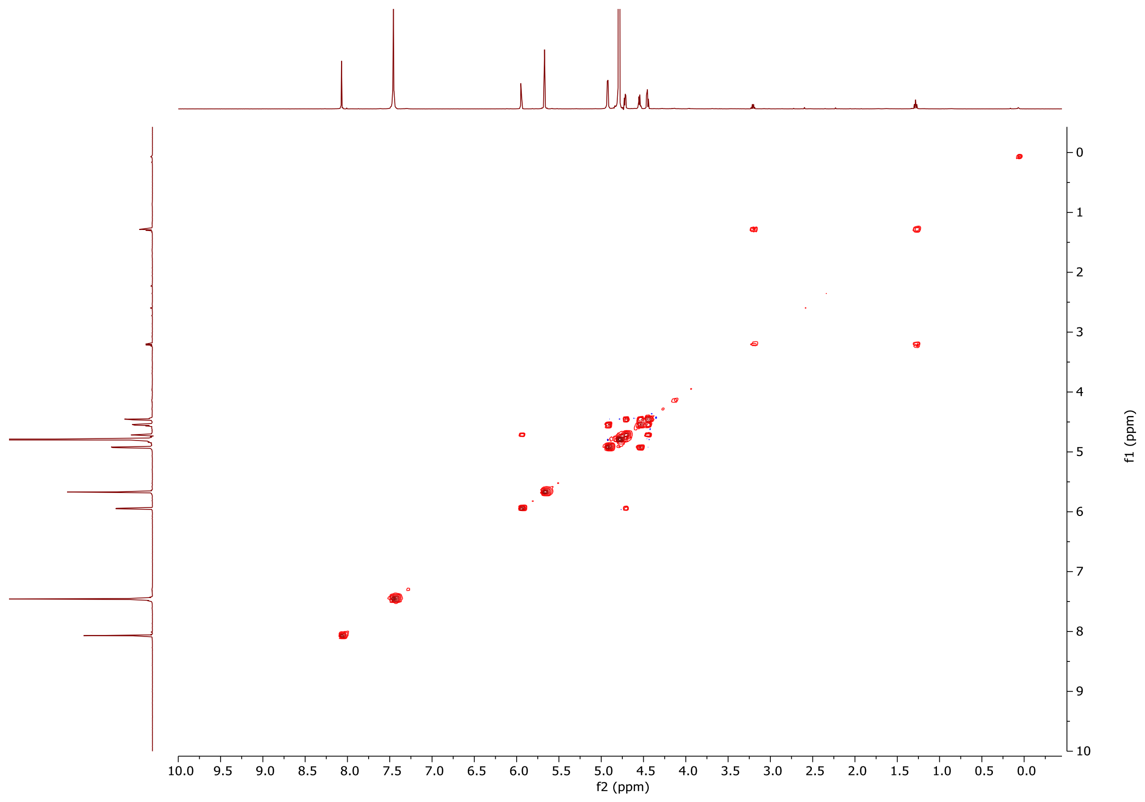

^{13}C- ^{1}H HSQC NMR	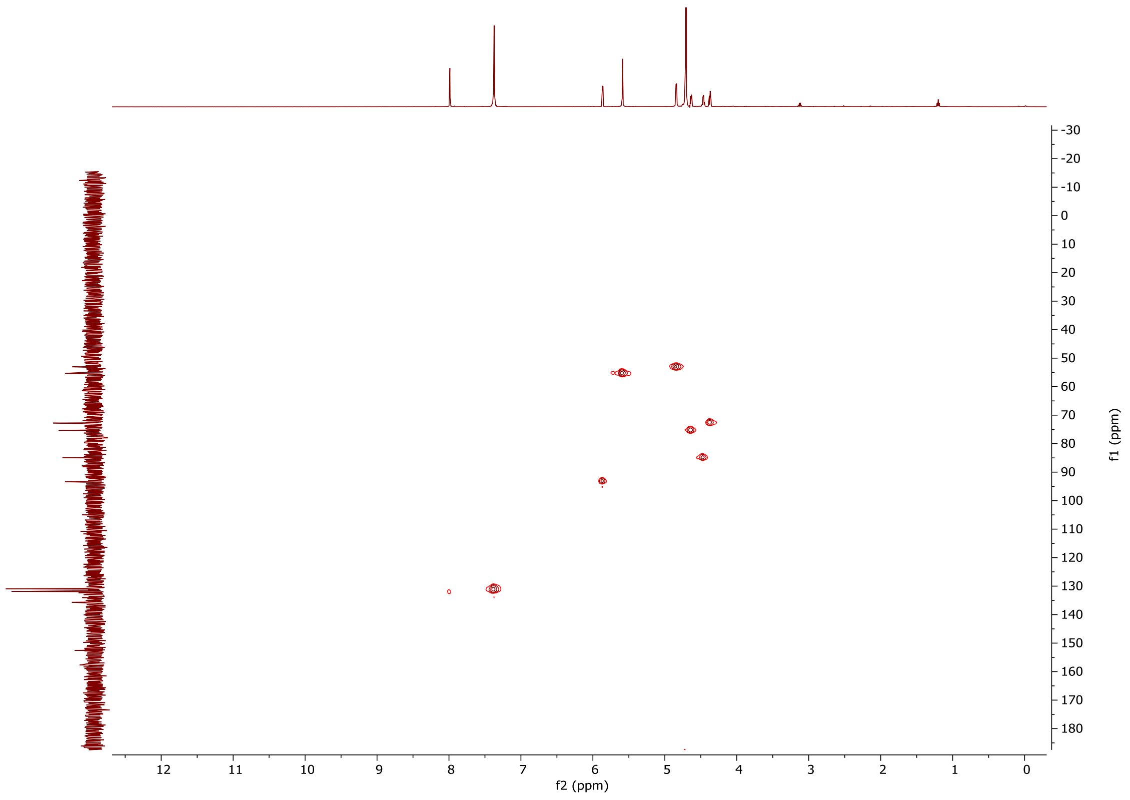
^{13}C NMR	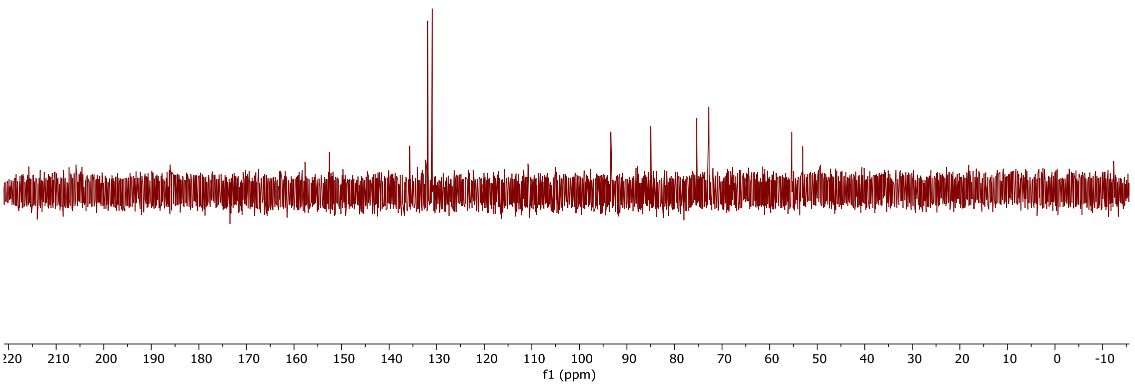

**8e,** 3Me-Bn^{7}G-triazolP	
HRMS	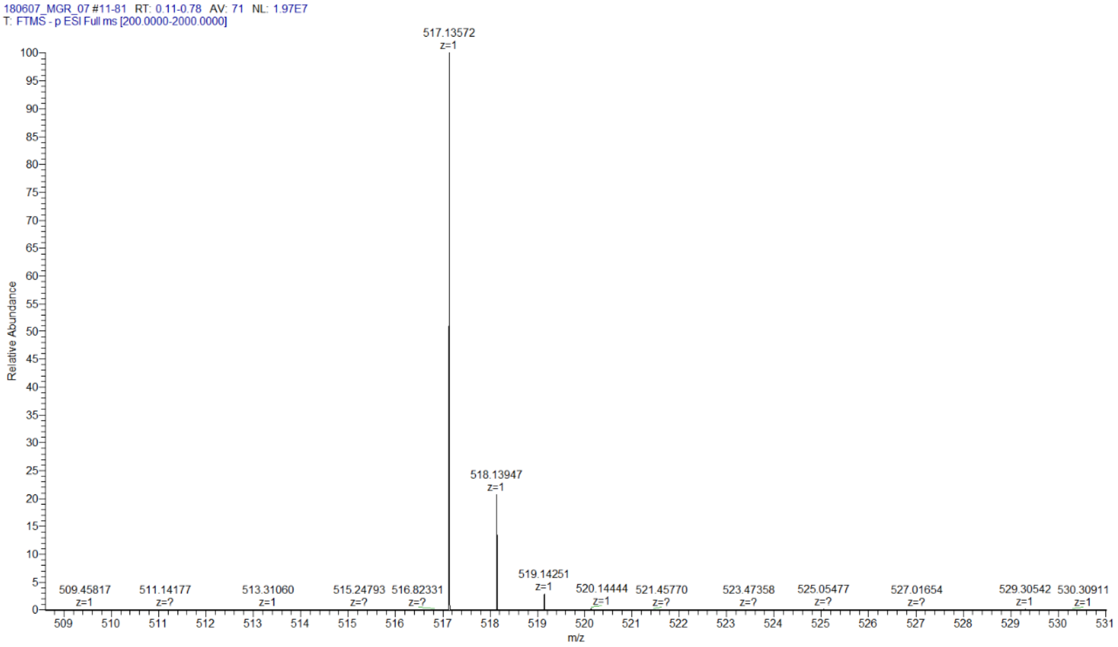
RP HPLC profile (Abs, AU, 254 nm)	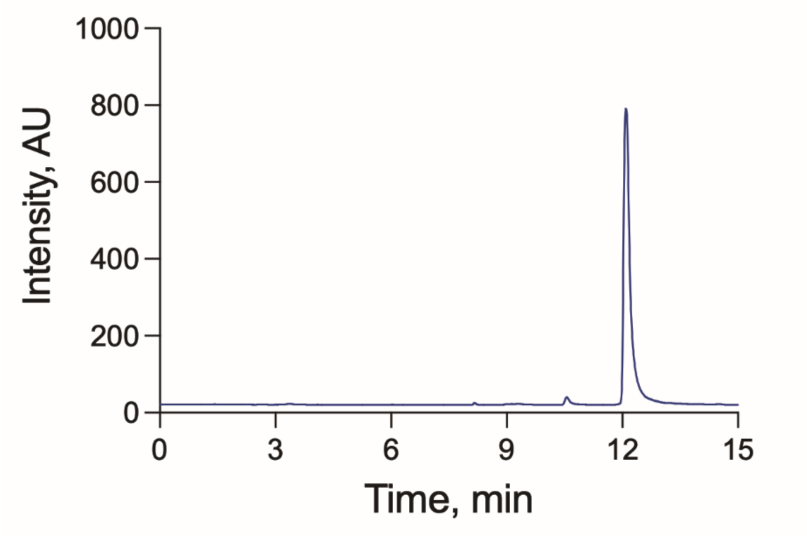

^{1}H NMR	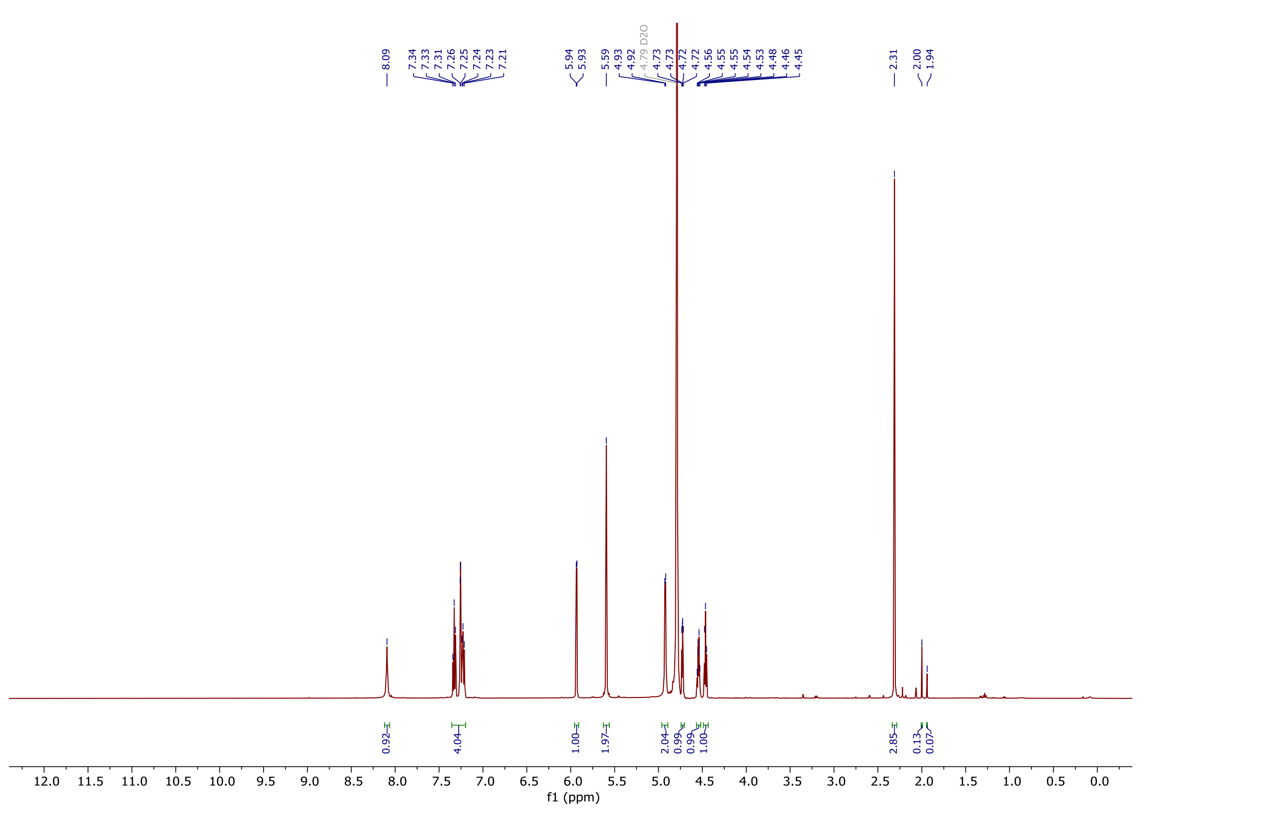
^{1}H-^{1}H COSY NMR	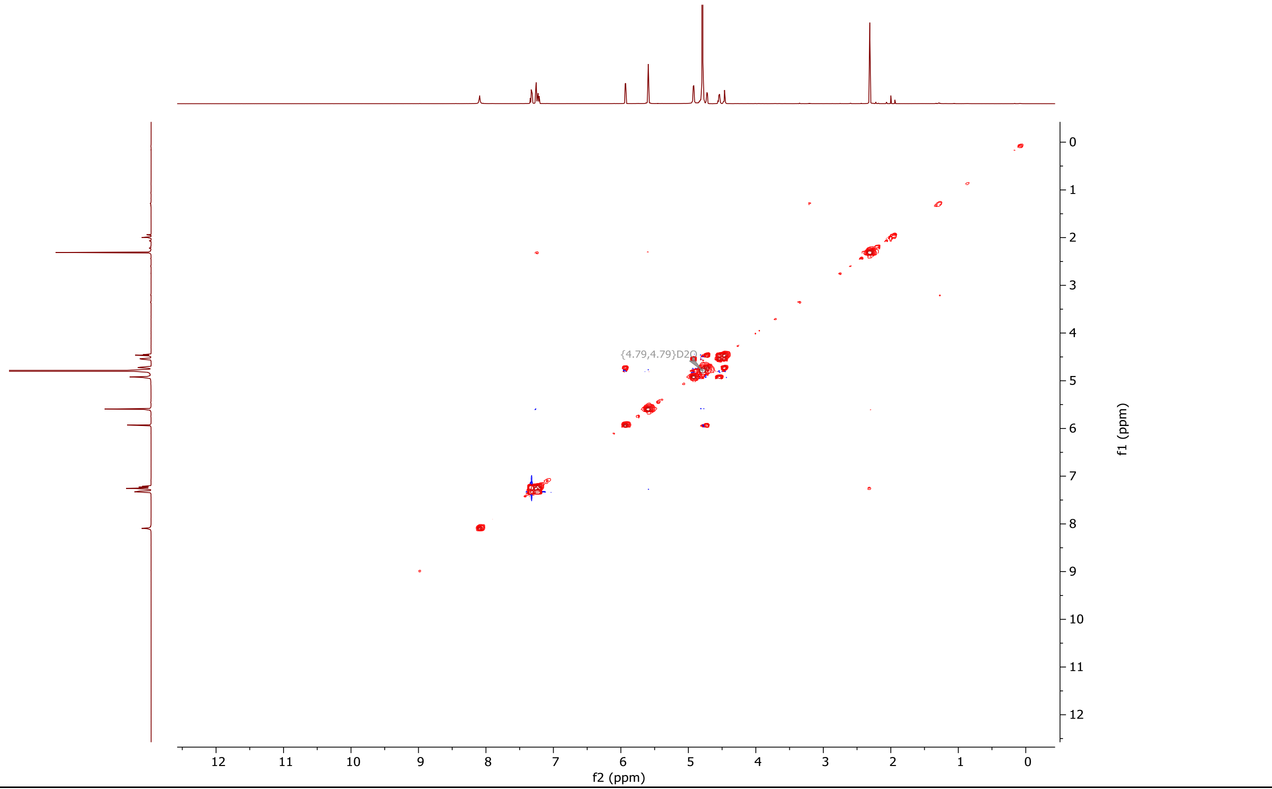

^{13}C- ^{1}H HSQC NMR

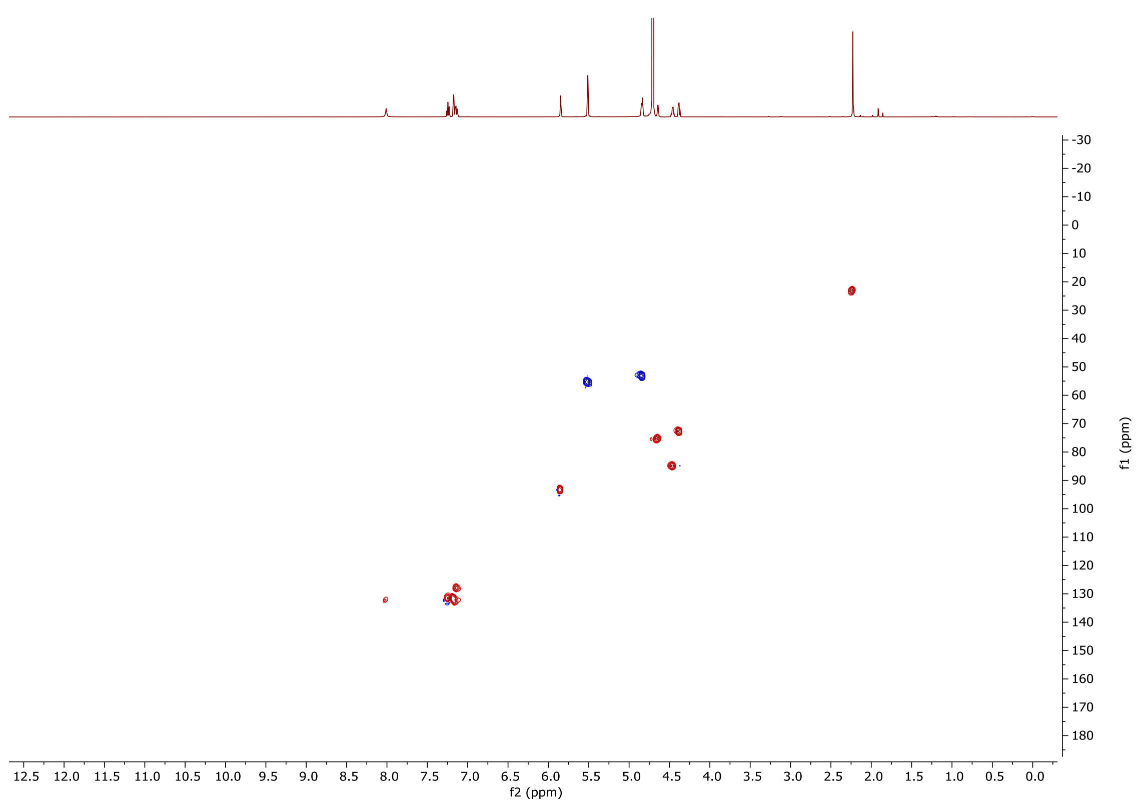

^{31}P NMR

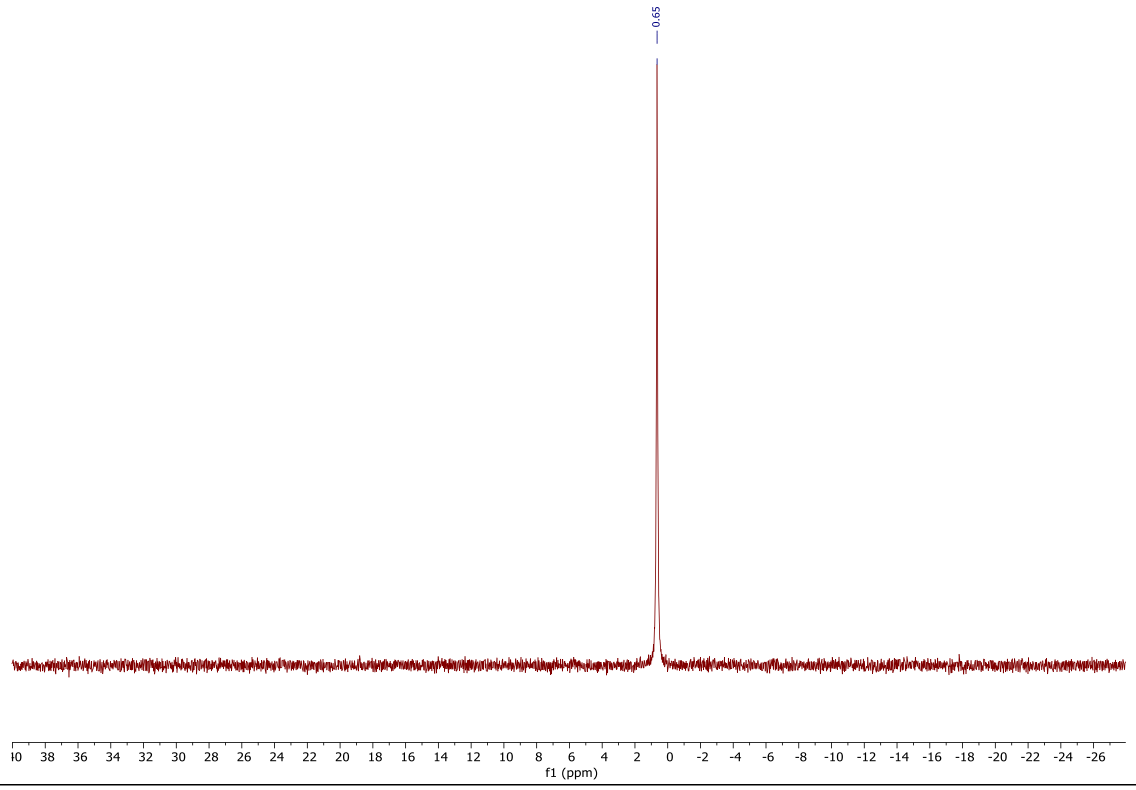

^{13}C NMR

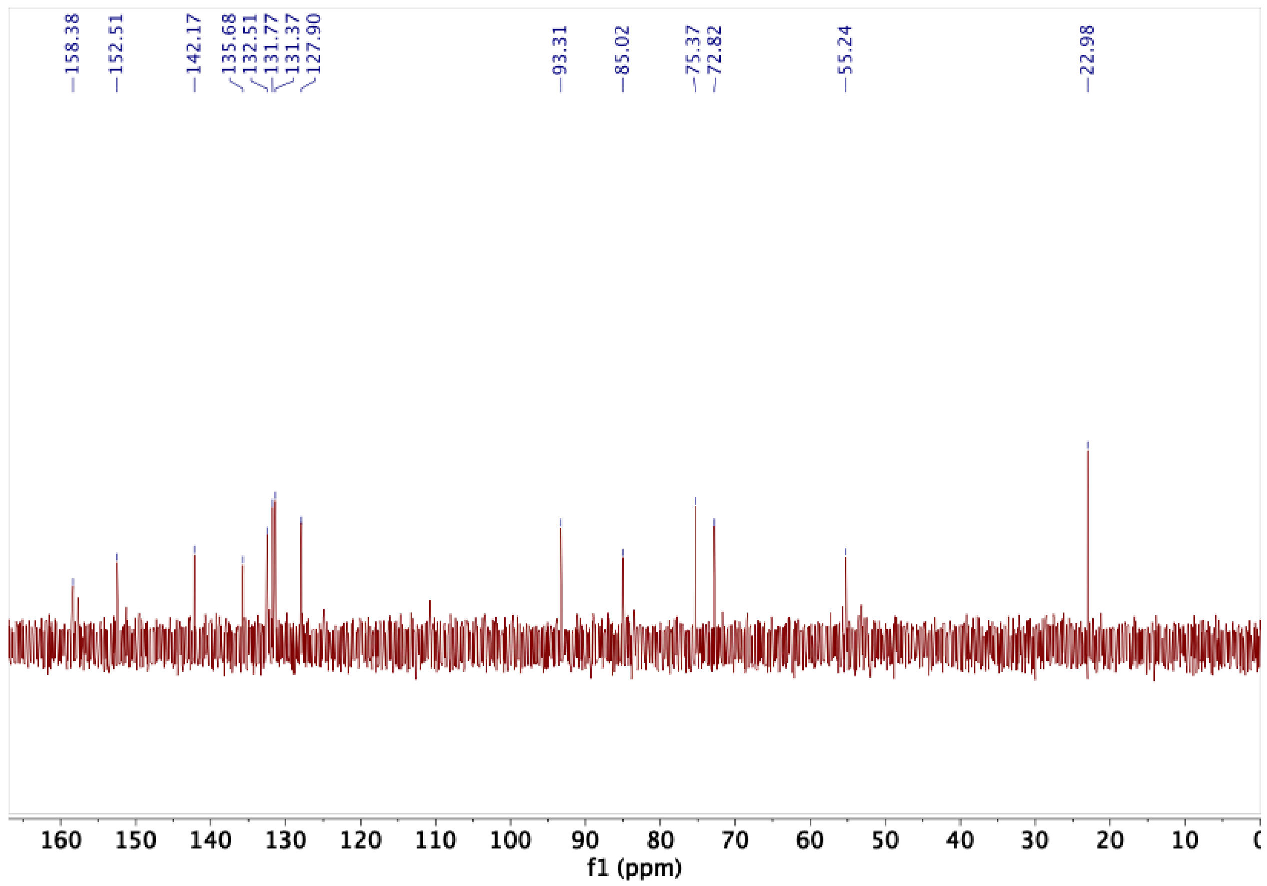

158.38
152.51
142.17
135.68
132.51
131.77
131.37
127.90
93.31
85.02
75.37
72.82
55.24
22.98
160 150 140 130 120 110 100 90 80 70 60 50 40 30 20 10
f1 (ppm)

**5’,** Bn[7]guanine	
^{1}H NMR	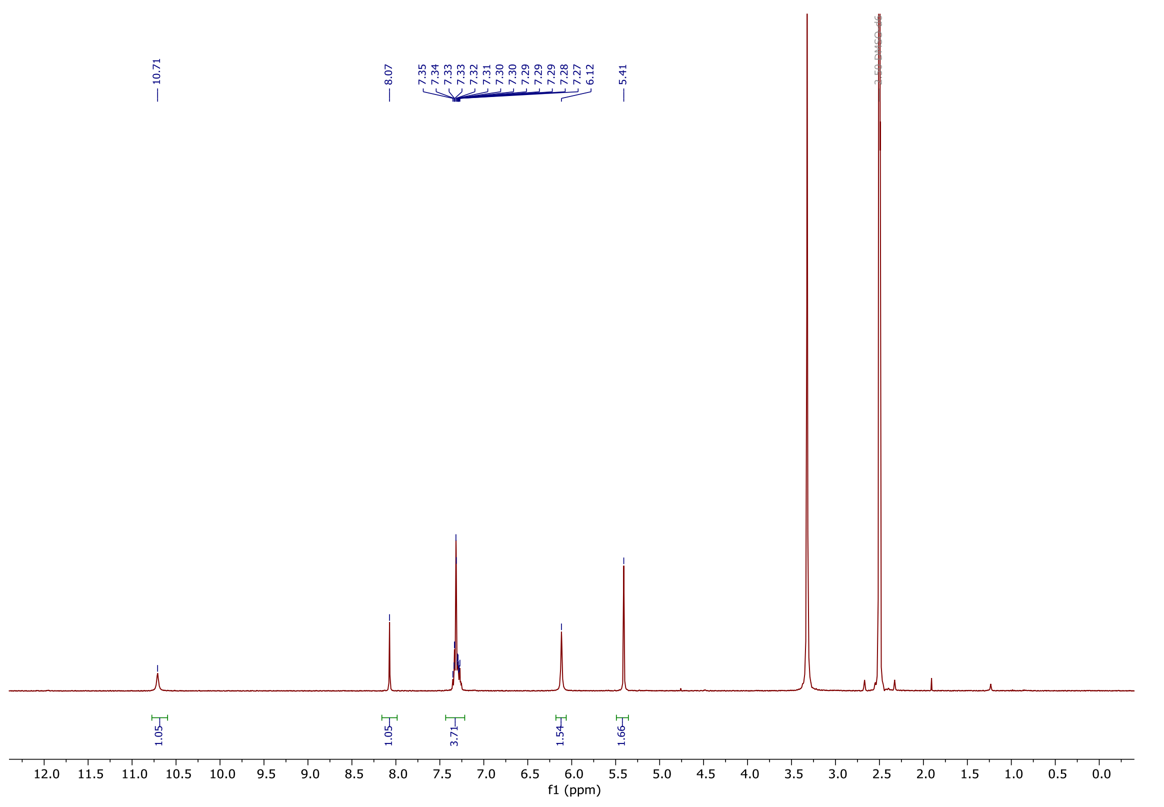
^{1}H- ^{1}H COSY NMR	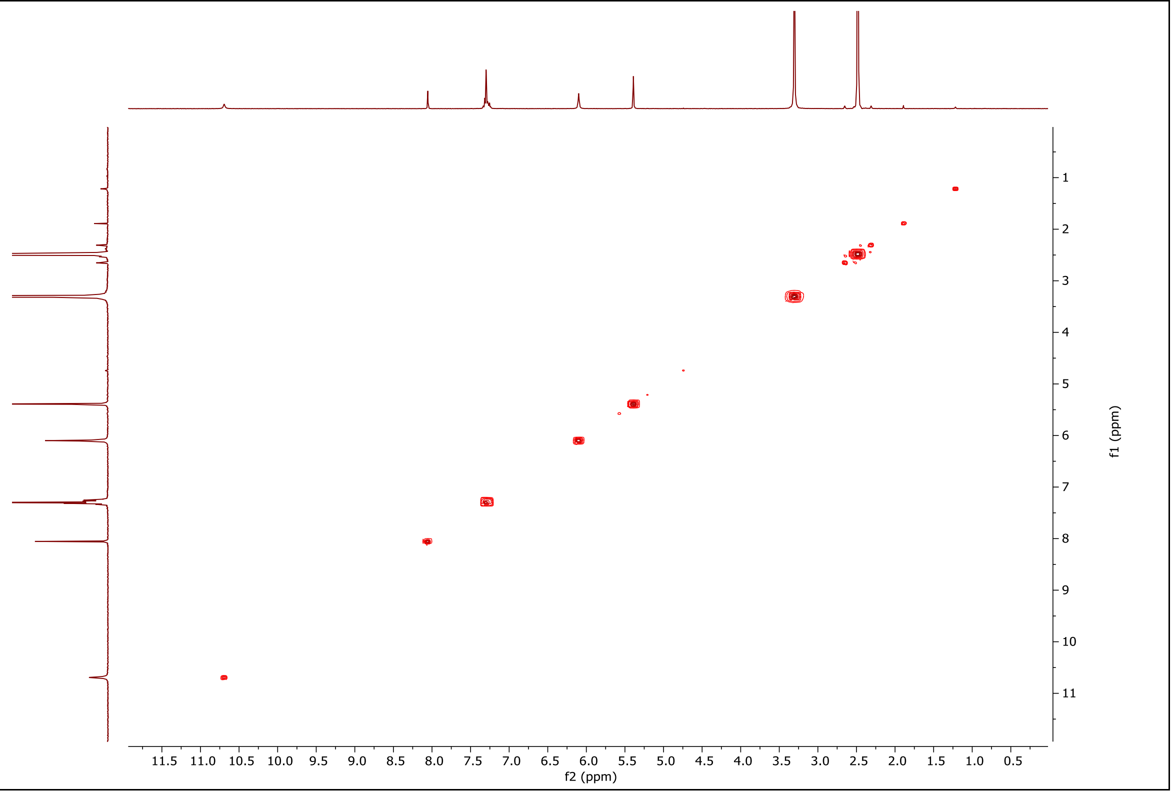

^{13}C- ^{1}H HSQC NMR

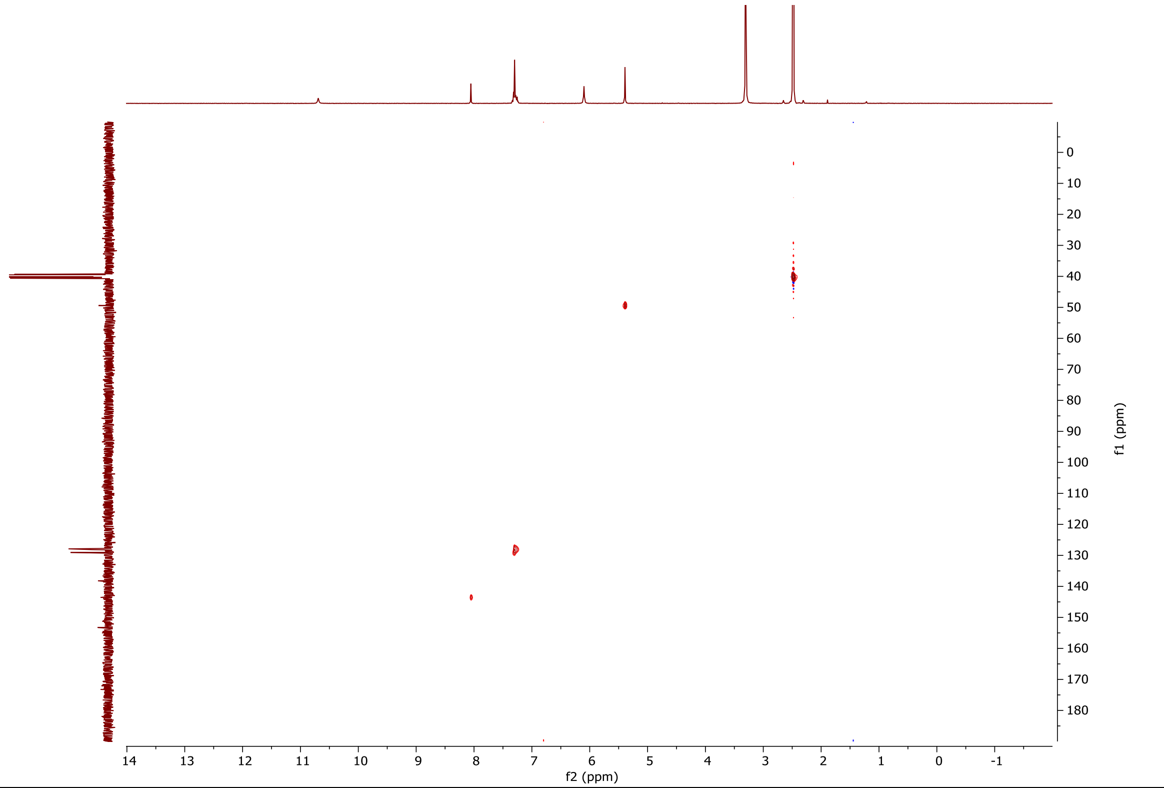

5a’, 3MeBn^{7}guanine

^{1}H NMR

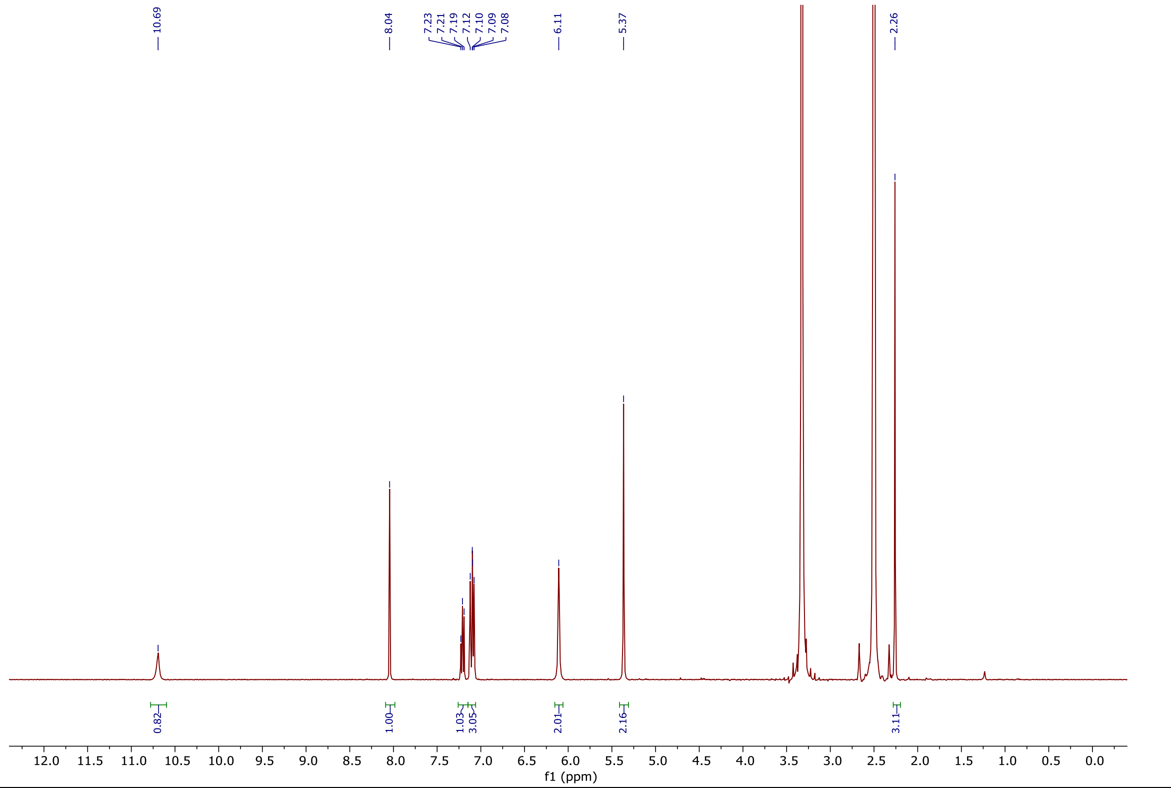

^{1}H- ^{1}H COSY NMR	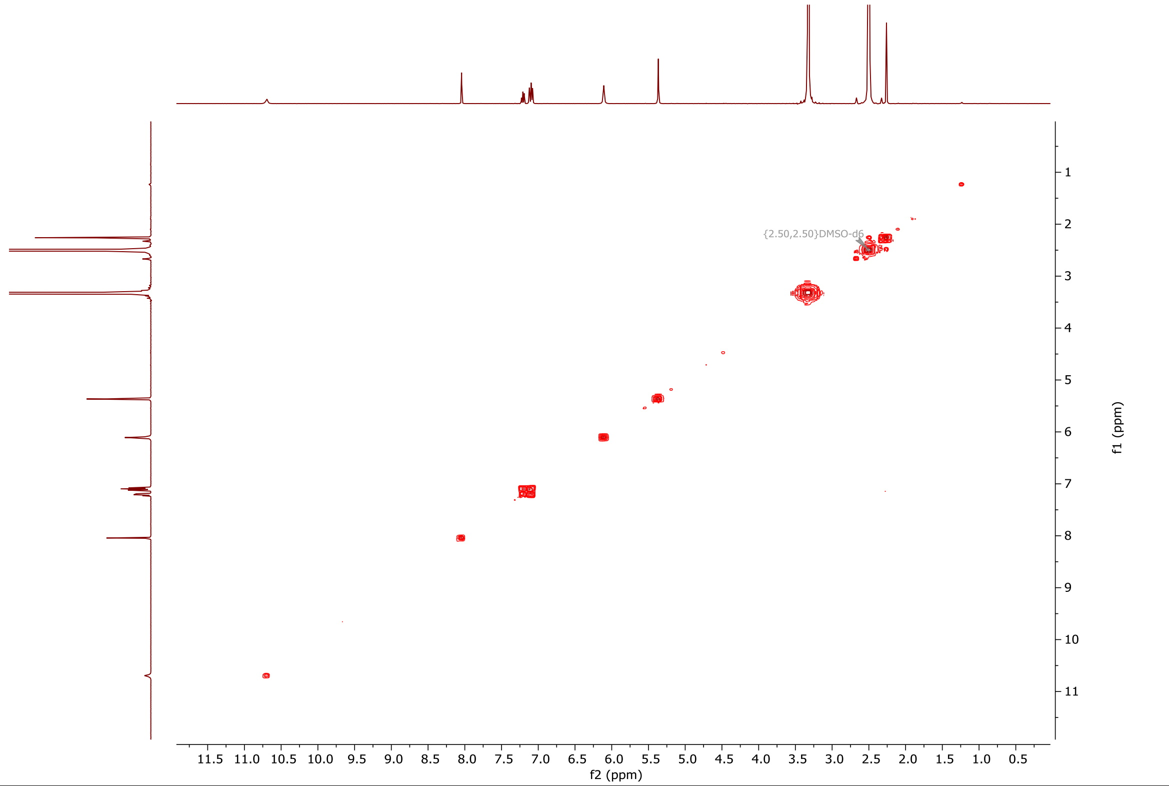
^{13}C- ^{1}H HSQC NMR	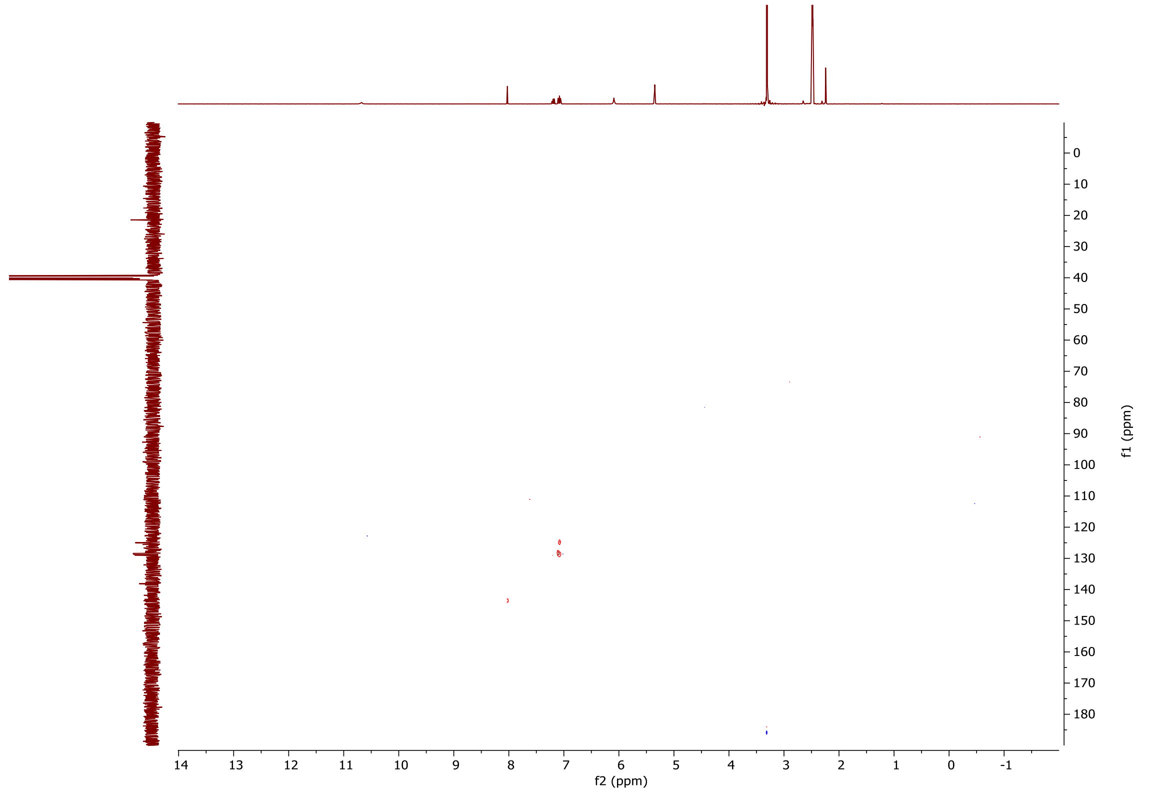

**5d’,** 3,4F$_2$Bn^{7}guanine	
^{1}H NMR	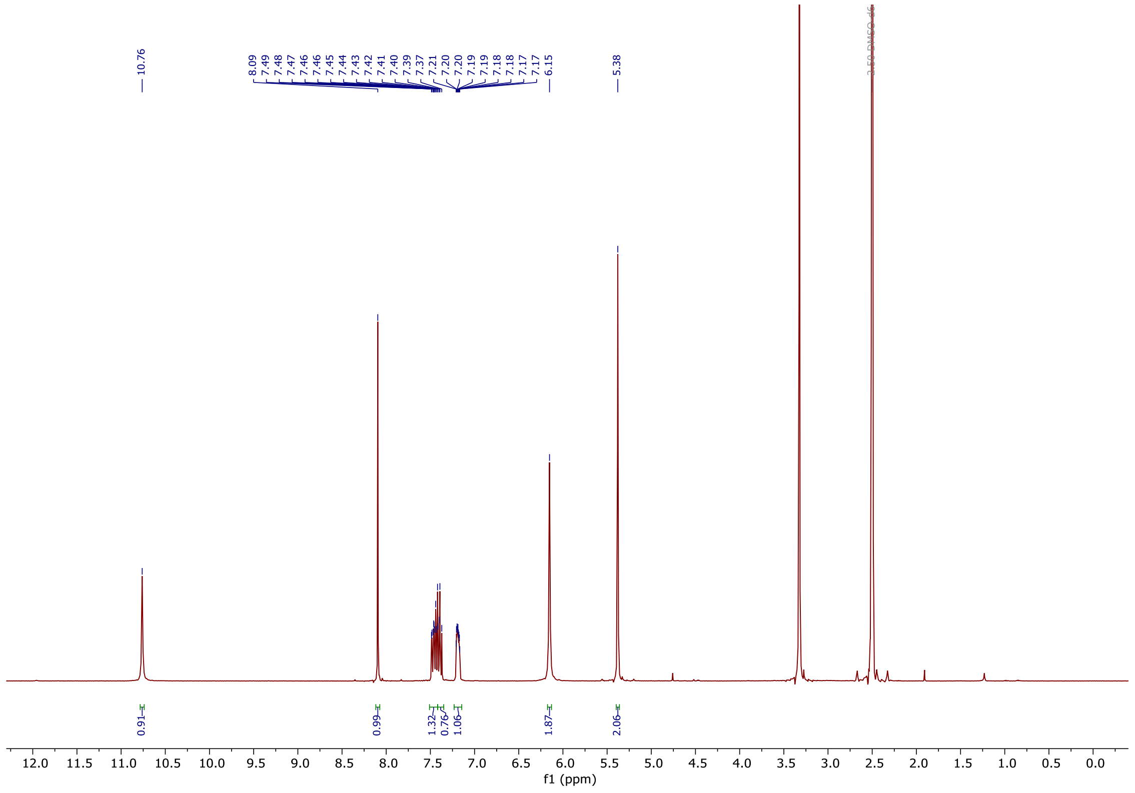
^{1}H- ^{1}H COSY NMR	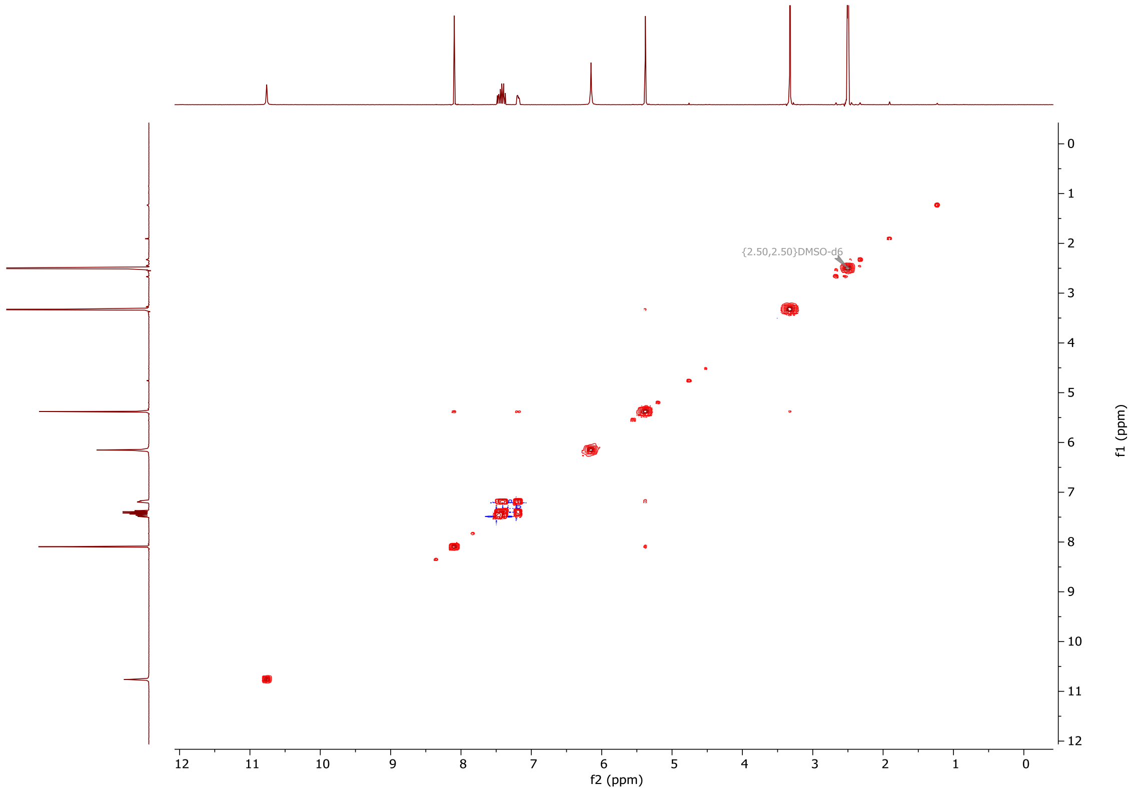

^{13}C- ^{1}H HSQC NMR	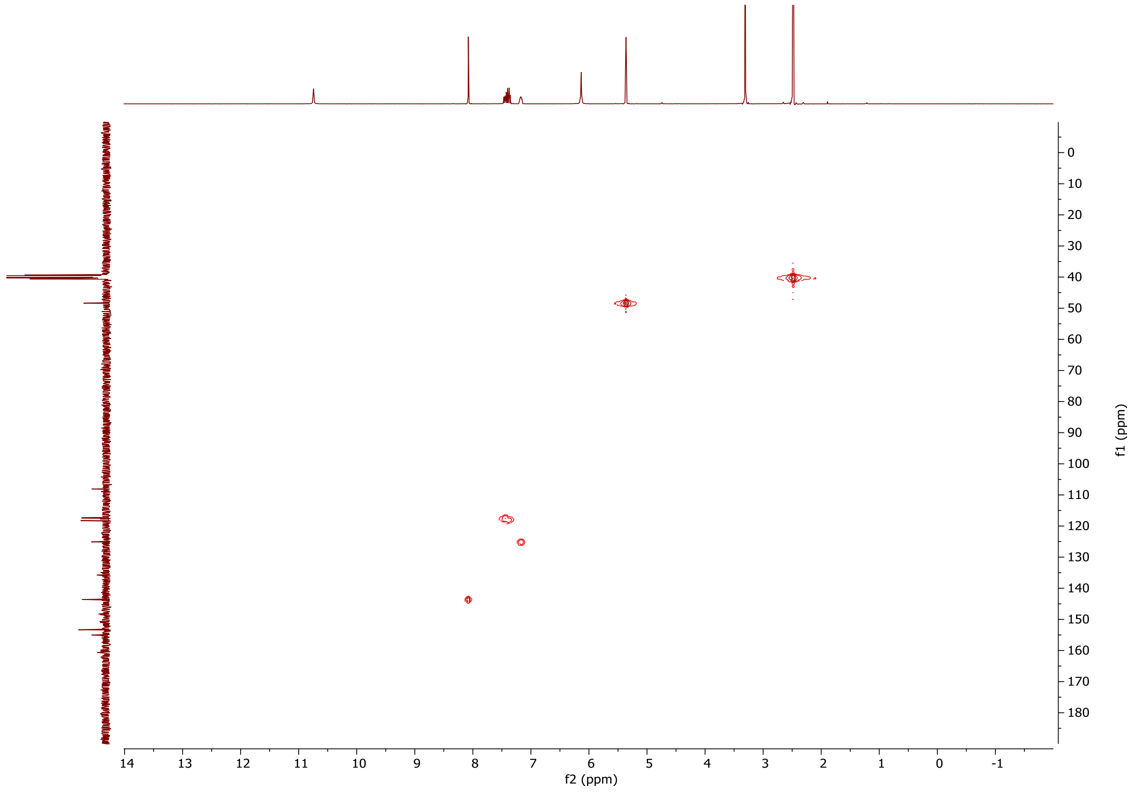
^{13}C NMR	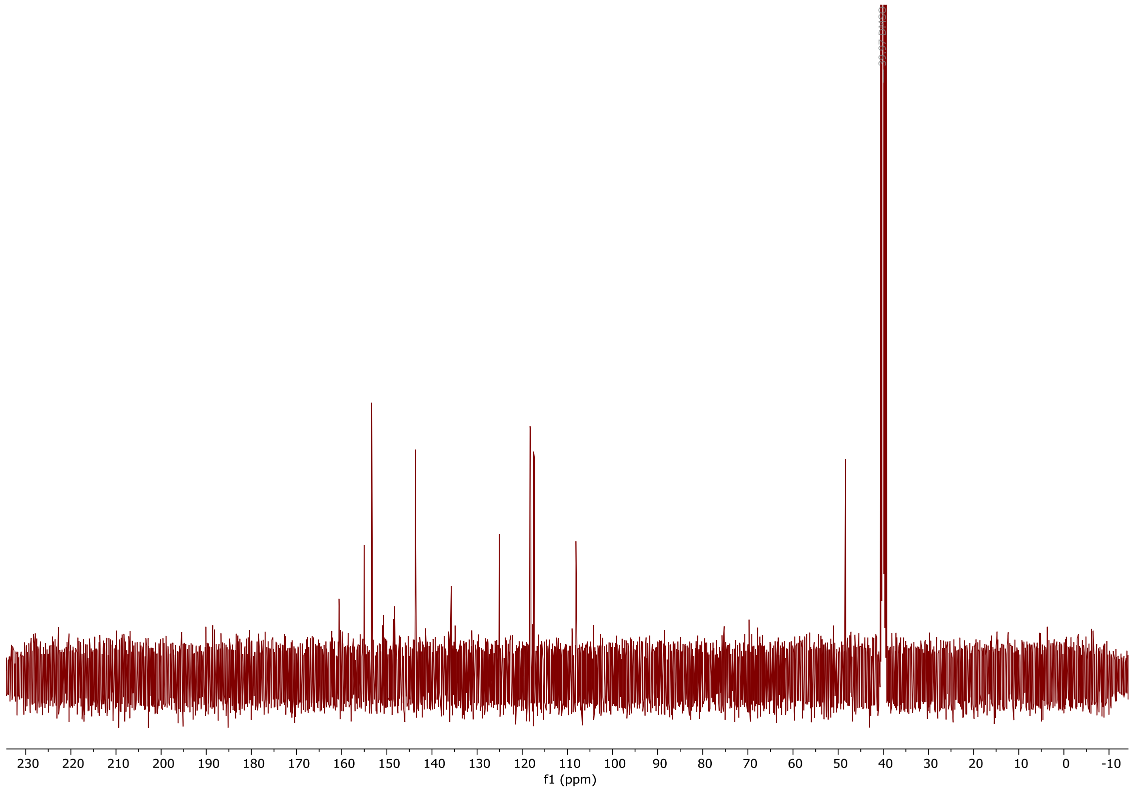

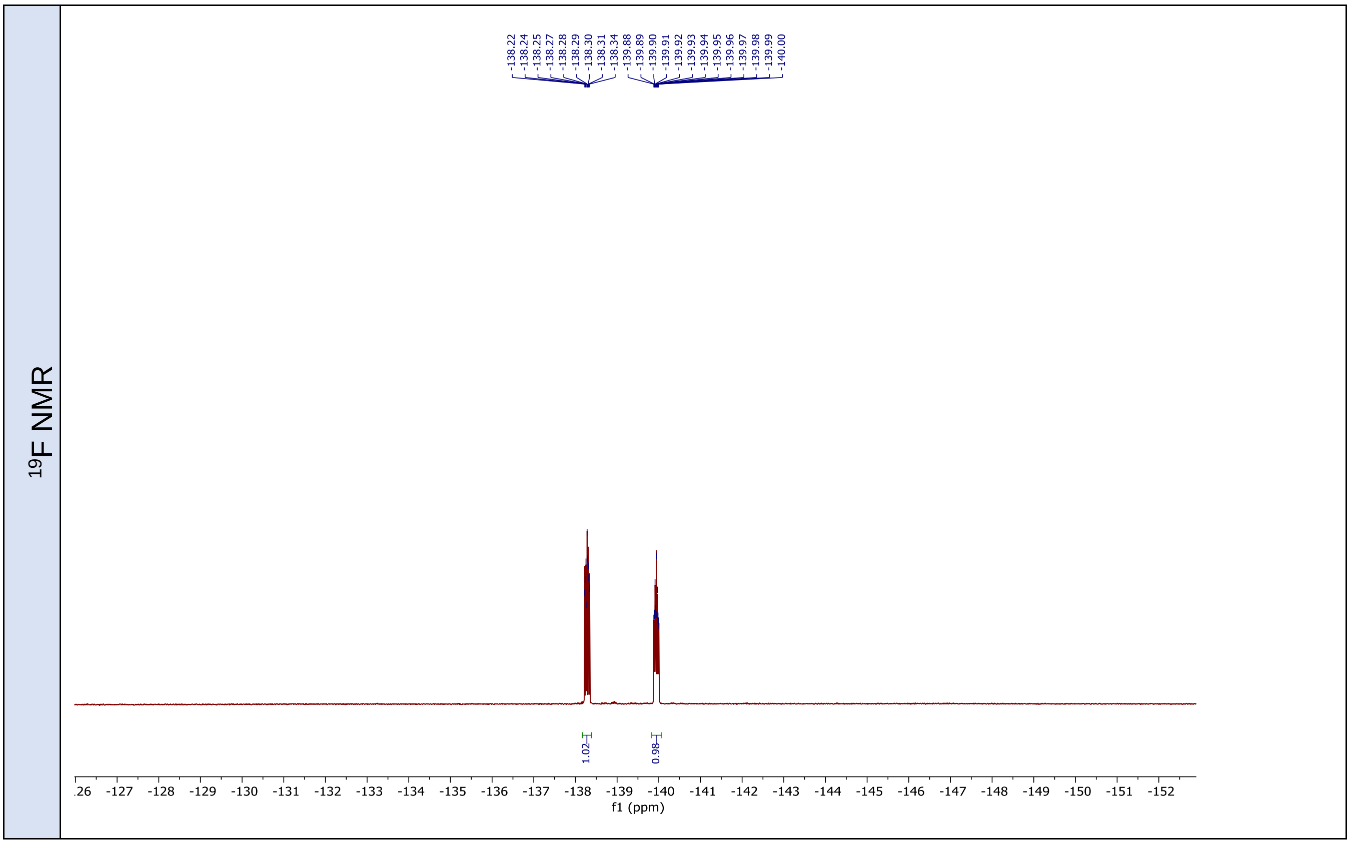
19F NMR
-138.22
-138.24
-138.25
-138.27
-138.28
-138.29
-138.30
-138.31
-138.34
-139.88
-139.89
-139.90
-139.91
-139.92
-139.93
-139.94
-139.95
-139.96
-139.97
-139.98
-139.99
-140.00
1.02
0.98
f1 (ppm)

5d'', (3,4F$_2$Bn^{7,9})$_2$guanine

^{1}H NMR

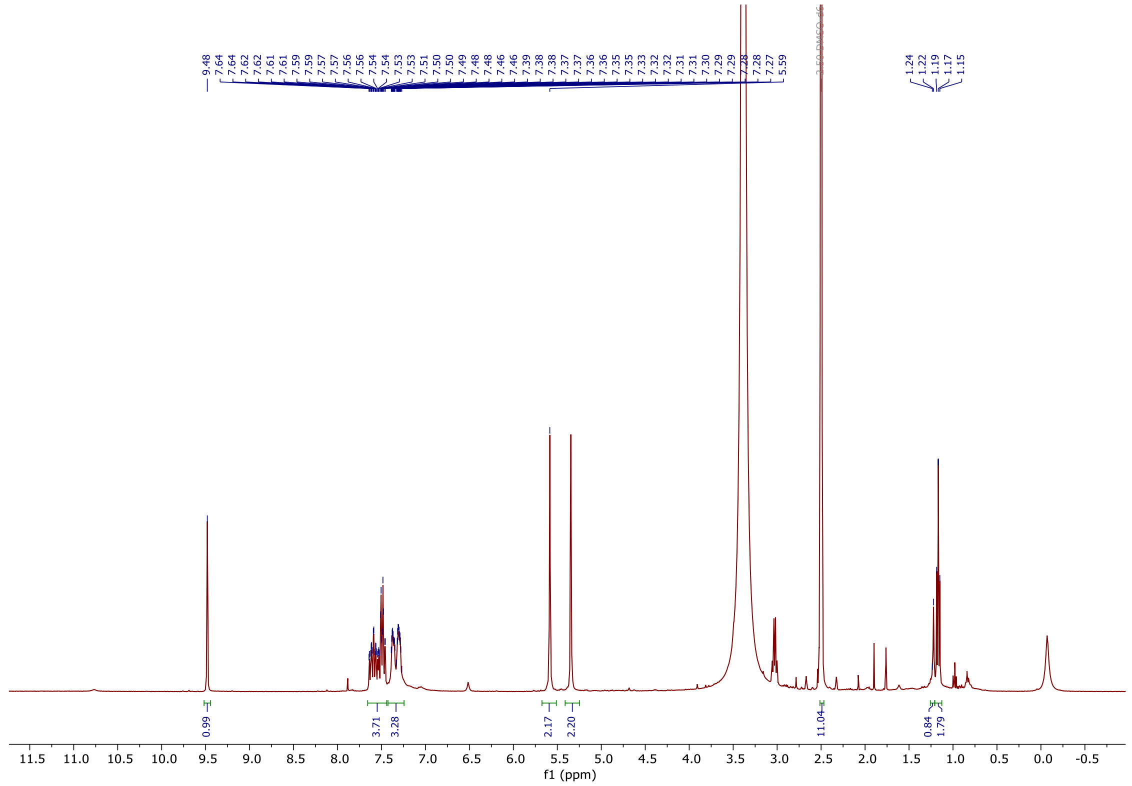

^{1}H- ^{1}H COSY NMR

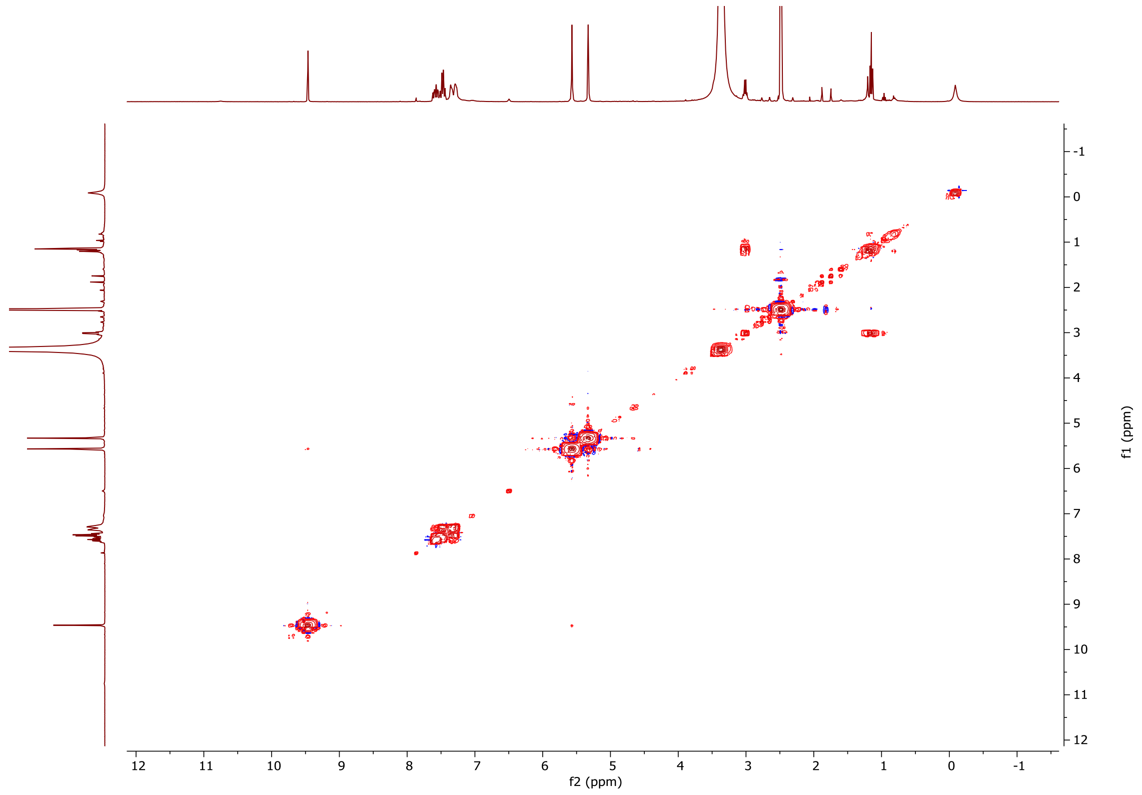

19F NMR

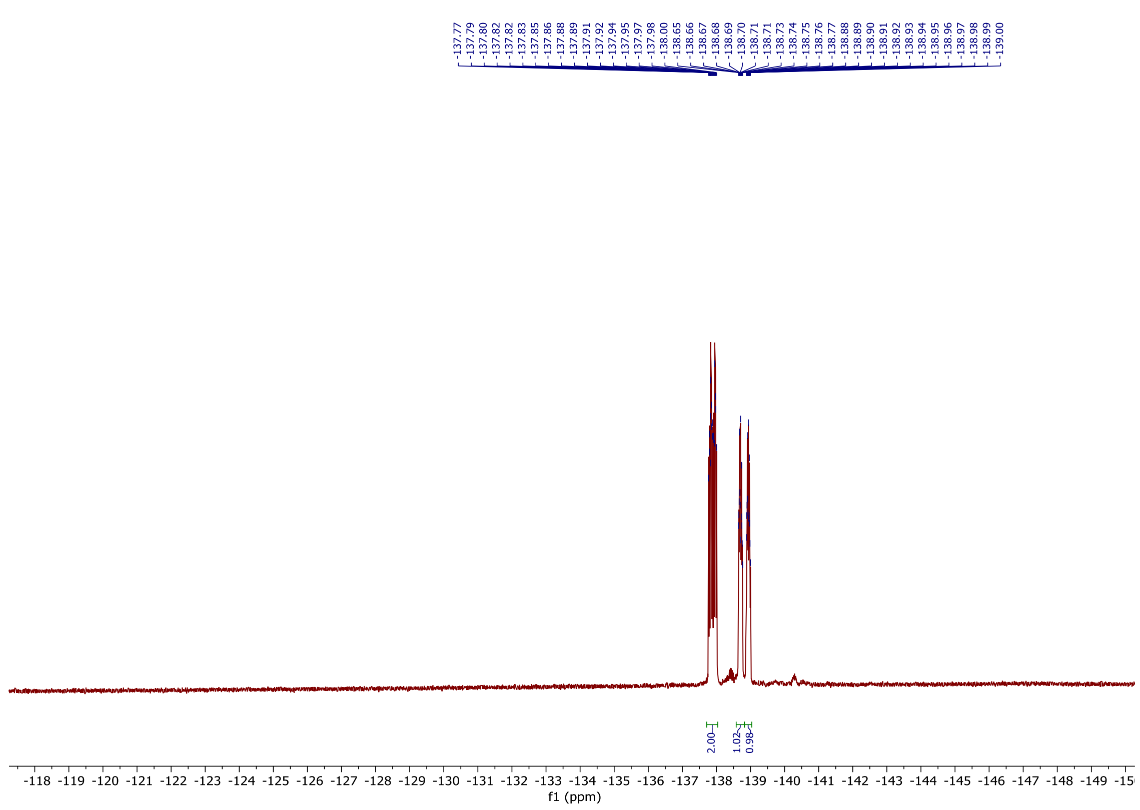

References

1. Kalayanov, G.; Jaksa, S.; Scarcia, T.; Kobe, J. Regioselective functionalization of guanine: Simple and practical synthesis of 7-and 9-alkylated guanines starting from guanosine. *Synthesis-Stuttgart* **2004**, (12), 2026-2034, Article. DOI: 10.1055/s-2004-829174.
2. Kasprzyk, R.; Starek, B. J.; Ciechanowicz, S.; Kubacka, D.; Kowalska, J.; Jemielity, J. Fluorescent Turn-On Probes for the Development of Binding and Hydrolytic Activity Assays for mRNA Cap-Recognizing Proteins. *Chemistry-a European Journal* **2019**, *25* (27), 6728-6740. DOI: 10.1002/chem.201900051.
3. Baranowski, M. R.; Nowicka, A.; Rydzik, A. M.; Warminski, M.; Kasprzyk, R.; Wojtczak, B. A.; Wojcik, J.; Claridge, T. D. W.; Kowalska, J.; Jemielity, J. Synthesis of Fluorophosphate Nucleotide Analogues and Their Characterization as Tools for 19F NMR Studies. *Journal of Organic Chemistry* **2015**, *80* (8), 3982-3997, Article. DOI: 10.1021/acs.joc.5b00337.
4. Kowalska, J.; Lewdorowicz, M.; Zuberek, J.; Grudzien-Nogalska, E.; Bojarska, E.; Stepinski, J.; Rhoads, R. E.; Darzynkiewicz, E.; Davis, R. E.; Jemielity, J. Synthesis and characterization of mRNA cap analogs containing phosphorothioate substitutions that bind tightly to eIF4E and are resistant to the decapping pyrophosphatase DcpS. *Rna* **2008**, *14* (6), 1119-1131, Article. DOI: 10.1261/rna.990208.
5. Wojtczak, B. A.; Sikorski, P. J.; Fac-Dabrowska, K.; Nowicka, A.; Warminski, M.; Kubacka, D.; Nowak, E.; Nowotny, M.; Kowalska, J.; Jemielity, J. 5 '-Phosphorothiolate Dinucleotide Cap Analogues: Reagents for Messenger RNA Modification and Potent Small-Molecular Inhibitors of Decapping Enzymes. *Journal of the American Chemical Society* **2018**, *140* (18), 5987-5999, Article. DOI: 10.1021/jacs.8b02597.
6. Wanat, P.; Walczak, S.; Wojtczak, B. A.; Nowakowska, M.; Jemielity, J.; Kowalska, J. Ethynyl, 2-Propynyl, and 3-Butynyl C-Phosphonate Analogues of Nucleoside Di- and Triphosphates: Synthesis and Reactivity in CuAAC. *Organic Letters* **2015**, *17* (12), 3062-3065, Article. DOI: 10.1021/acs.orglett.5b01346.
7. Walczak, S.; Nowicka, A.; Kubacka, D.; Fac, K.; Wanat, P.; Mroczek, S.; Kowalska, J.; Jemielity, J. A novel route for preparing 5 ' cap mimics and capped RNAs: phosphate-modified cap analogues obtained via click chemistry. *Chemical Science* **2017**, *8* (1), 260-267, Article. DOI: 10.1039/c6sc02437h.
8. Walczak, S.; Sikorski, P. J.; Kasprzyk, R.; Kowalska, J.; Jemielity, J. Exploring the potential of phosphotriazole 5 ' mRNA cap analogues as efficient translation initiators. *Organic & Biomolecular Chemistry* **2018**, *16* (36), 6741-6748, Article. DOI: 10.1039/c8ob01720d.

9. Kozarski, M.; Kubacka, D.; Wojtczak, B. A.; Kasprzyk, R.; Baranowski, M. R.; Kowalska, J. 7-Methylguanosine monophosphate analogues with 5 '-(1,2,3-triazoyl) moiety: Synthesis and evaluation as the inhibitors of cNIIIB nucleotidase. *Bioorganic & Medicinal Chemistry* **2018**, *26* (1), 191-199. DOI: 10.1016/j.bmc.2017.11.032.
10. Kopcial, M.; Wojtczak, B. A.; Kasprzyk, R.; Kowalska, J.; Jemielity, J. N1-Propargylguanosine Modified mRNA Cap Analogs: Synthesis, Reactivity, and Applications to the Study of Cap-Binding Proteins. *Molecules* **2019**, *24* (10), 17, Article. DOI: 10.3390/molecules24101899.
11. Rydzik, A. M.; Lukaszewicz, M.; Zuberek, J.; Kowalska, J.; Darzynkiewicz, Z. M.; Darzynkiewicz, E.; Jemielity, J. Synthetic dinucleotide mRNA cap analogs with tetraphosphate 5 ',5 ' bridge containing methylenebis(phosphonate) modification. *Organic & Biomolecular Chemistry* **2009**, *7* (22), 4763-4776, Article. DOI: 10.1039/b911347a.
12. Jemielity, J.; Lukaszewicz, M.; Kowalska, J.; Czarnecki, J.; Zuberek, J.; Darzynkiewicz, E. Synthesis of biotin labelled cap analogue - incorporable into mRNA transcripts and promoting cap-dependent translation. *Organic & Biomolecular Chemistry* **2**
13. Warminski, M.; Kowalska, J.; Buck, J.; Zuberek, J.; Lukaszewicz, M.; Nicola, C.; Kuhn, A. N.; Sahin, U.; Darzynkiewicz, E.; Jemielity, J. The synthesis of isopropylidene mRNA cap analogs modified with phosphorothioate moiety and their evaluation as promoters of mRNA translation. *Bioorganic & Medicinal Chemistry Letters* **2013**, *23* (13), 3753-3758, Article. DOI: 10.1016/j.bmcl.2013.05.001.
14. Warminski, M.; Warminska, Z.; Kowalska, J.; Jemielity, J. mRNA Cap Modification through Carbamate Chemistry: Synthesis of Amino- and Carboxy-Functionalised Cap Analogues Suitable for Labelling and Bioconjugation. *European Journal of Organic Chemistry* **2015**, *2015* (28), 6153-6169, Article. DOI: 10.1002/ejoc.201500672.
15. Bednarek, S.; Madan, V.; Sikorski, P. J.; Bartenschlager, R.; Kowalska, J.; Jemielity, J. mRNAs biotinylated within the 5 ' cap and protected against decapping: new tools to capture RNA - protein complexes. *Philosophical Transactions of the Royal Society B-Biological Sciences* **2018**, *373* (1762), 12, Article. DOI: 10.1098/rstb.2018.0167.
16. Lee, G. H.; Lim, H. K.; Hah, S. S. Preparation of 5 '-Azido-5 '-Deoxyguanosine and Its Efficiency for Click Chemistry. *Bulletin of the Korean Chemical Society* **2011**, *32* (10), 3767-3769, Article. DOI: 10.5012/bkcs.2011.32.10.3767.
17. Sun, Q.; Liu, S.; Sun, J.; Gong, S. S.; Xiao, Q.; Shen, L. One-pot synthesis of symmetrical P-1,P-2-dinucleoside-5 '-diphosphates from nucleoside-5 '-H-phosphonates: mechanistic insights into reaction path. *Tetrahedron Letters* **2013**, *54* (29), 3842-3845, Article. DOI: 10.1016/j.tetlet.2013.05.040.
